# Synthesis of pyrrole-fused dibenzoxazepine/dibenzothiazepine/triazolobenzodiazepine derivatives via isocyanide-based multicomponent reactions

**DOI:** 10.3762/bjoc.20.241

**Published:** 2024-11-11

**Authors:** Marzieh Norouzi, Mohammad Taghi Nazeri, Ahmad Shaabani, Behrouz Notash

**Affiliations:** 1 Department of Organic Chemistry, Shahid Beheshti University, Daneshjou Boulevard, Tehran, 1983969411, Iran; 2 Department of Inorganic Chemistry, Shahid Beheshti University, Daneshjou Boulevard, Tehran, 1983969411, Iran

**Keywords:** cyclic imines, dibenzothiazepine, dibenzoxazepine, isocyanides, multicomponent reactions, pyrrole, triazolobenzodiazepine

## Abstract

An efficient and facile synthesis of pyrrole-fused dibenzoxazepine/dibenzothiazepine/triazolobenzodiazepine derivatives was developed through the isocyanide-based multicomponent reaction of isocyanides, *gem*-diactivated olefins, and cyclic imines such as dibenzoxazepine, dibenzothiazepine, and triazolobenzodiazepine under solvent- and catalyst-free conditions. Purposefully, this approach produced various bioactive scaffolds using environmentally friendly, mild, and simple conditions. Due to their bioactive moieties, these compounds with exclusive fluorescence properties may attract great attention in biomedical applications, clinical diagnostics, and conjugate materials.

## Introduction

**Figure 1:**
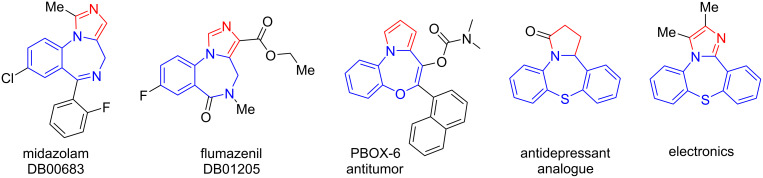
Representation of distinguished structures of benzodiazepine/benzoxazepine/benzothiazepine with pharmaceutical to electronic applications.

Pyrroles and their derivatives are important *N*-heterocyclic compounds with antibiotic, antiviral, and anticancer properties that are found in many drugs and natural products [1–6]. Pyrroles' biological properties manifest when they are fused to other heterocycles [7–12]. Among them, seven-membered heterocycles of the benzodiazepine, benzoxazepine, and benzothiazepine derivatives are especially important. These consitute the central core of many natural and biological compounds and commercial drugs, including diazepam, clonazepam, lorazepam, telenzepine, chlordiazepoxide, loxapine, and amoxapine [13–21]. Pyrrole-fused benzodiazepines, benzoxazepines, or benzothiazepines exhibit unique biological and pharmacological properties [22–23]. For example, midazolam is a hypnotic-sedative drug with anxiolytic, muscle relaxant, and anticonvulsant properties [24], flumazenil is made to induce general anesthesia for diagnostic and therapeutic procedures [25] or PBOX-6 is a drug for the treatment of depression [26]. Benzothiazepines are known as antidepressants and are molecules with interesting electronic properties (Figure 1) [27]. Accordingly, the synthesis of new derivatives of pyrrole-fused benzodiazepine/benzoxazepine/benzothiazepine is very important.

**Scheme 1:**
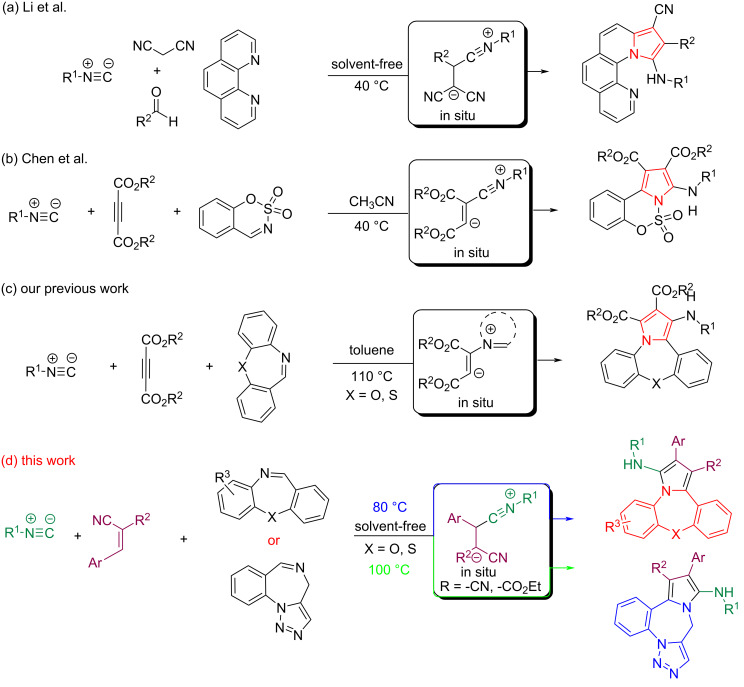
Methods for the construction of pyrrole-fused heterocycles through I-MCR reactions.

Due to the wide applications of pyrrole-fused heterocycles, very diverse approaches have been developed for their synthesis [28–32]. In recent years, multicomponent reactions (MCRs) have emerged as one of the most efficient and powerful methods to achieve this goal [7,33]. Among them, isocyanide-based multicomponent reactions (I-MCRs) are one of the well-known strategies in this field due to their operational simplicity, one-pot, convergent properties and atom economy, high efficiency, and high levels of chemical selectivity [34–36]. In addition to the application of isocyanides in a variety of MCRs, one of the unique reactions involves the formation of zwitterions from isocyanides upon reaction with acetylene and active olefin compounds such as alkyl acetylenedicarboxylates and *gem*-diactivated olefins. Due to having nucleophilic and electrophilic sites simultaneously in their structure, these zwitterions are able to participate in various cyclization processes, especially for the synthesis of pyrroles [37–40]. For example, Li et al. developed a one-pot four-component reaction (4-CR) of malononitrile, aldehydes, and isocyanides with 1,10-phenanthroline as cyclic imine under solvent-free conditions for the synthesis of pyrrole-fused phenanthroline. This reaction proceeds via in situ formation of zwitterion **I** through reaction of the aldehyde and malononitrile followed by 1,3-dipolar cycloaddition (Scheme 1a) [41]. Chen and co-workers reported a one-pot three-component reaction (3-CR) of sulfamate‐derived cyclic imine, isocyanide, and acetylenedicarboxylate. In this reaction too, the pyrrole-fused sulfamate is synthesized through intermediacy of the in situ-formed zwitterion **II** and [1 + 2 + 2] annulation reaction (Scheme 1b) [42–43]. Another I-MCR for forming pyrroles is the in situ formation reaction of the zwitterion **III**, known as Huisgens 1,4-dipole. The latter is formed by the reaction of imines with acetylenedicarboxylates and can be trapped by an isocyanide through a [4 + 1] cyclization reaction to synthesize pyrroles. In our recent studies, we prepared pyrrole-fused dibenzoxazepines via an Ugi reaction. Here, the reaction of benzoxazepine imine and acetylenedicarboxylate leads to the Huisgens 1,4-dipole zwitterion in situ, which is trapped by an isocyanide through the cyclization process (Scheme 1c) [44–45].

Here, we report an efficient and facile approach for the synthesis of pyrrole-fused dibenzoxazepine, dibenzothiazepine, and triazolobenzodiazepine derivatives via I-MCRs of *gem*-diactivated olefins, isocyanides, and cyclic imines (dibenzoxazepines, benzothiazepine, and triazolobenzodiazepine) under solvent- and catalyst-free conditions (Scheme 1d).

## Results and Discussion

### Synthesis

**Scheme 2:**
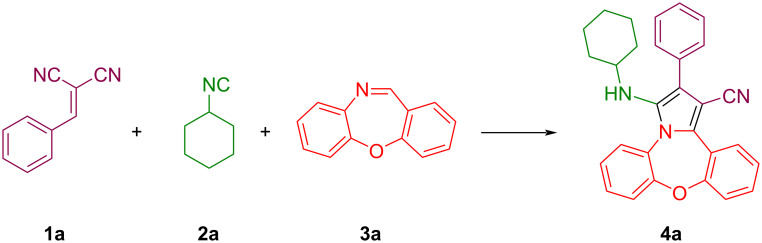
The model reaction of dibenzoxazepine, *gem*-diactivated olefin (2-benzylidenemalononitrile), and cyclohexyl isocyanide.

Dibenzoxazepine as imine component, cyclohexyl isocyanide, and the *gem*-diactivated olefin (2-benzylidenemalononitrile) were selected as the starting materials to screen the reaction conditions (Scheme 2, Table 1). First, we investigated the reaction in dichloromethane at room temperature and at 40 °C (Table 1, entries 1 and 2) and we found that the reaction progressed slightly at 40 °C. This promising result prompet us to examine the reaction in multiple anhydrous solvents such as CH_3_CN, toluene, EtOH, THF, EtOAc, and DMF at different temperatures (Table 1, entries 3–9). The result obtained from the study of solvents showed that pyrrole-fused dibenzoxazepine **4a** was obtained with a yield of 56% in ethanol as solvent at a temperature of 78 °C (Table 1, entry 4). To achieve a higher yield of product **4a**, the reaction was also attempted under solvent-free conditions at different temperatures (Table 1, entries 10–13). Interestingly, the highest yield of 70% of the desired product was achieved by conducting the reaction at 100 °C without using any solvent (Table 1, entry 12).

**Table 1:** Optimization of the reaction conditions.^a^

Entry	Solvent	Temperature (°C)	Time (h)	Yield^b^ (%)

1	CH_2_Cl_2_	25	24	0
2	CH_2_Cl_2_	40	24	23
3	CH_3_CN	82	24	36
4	EtOH	78	24	56
5	toluene	110	24	33
6	THF	66	24	41
7	H_2_O	100	24	trace
8	EtOAc	77	24	22
9	DMF	140	24	38
10	solvent-free	25	24	0
11	solvent-free	80	2	61
12	solvent-free	100	2	70
13	solvent-free	120	2	70

^a^Reaction conditions: **1a** (0.55 mmol), **2a** (0.55 mmol), and **3a** (0.50 mmol) were stirred in 2 mL of solvent in an oil bath at different temperatures and times. ^b^Isolated yields.

**Scheme 3:**
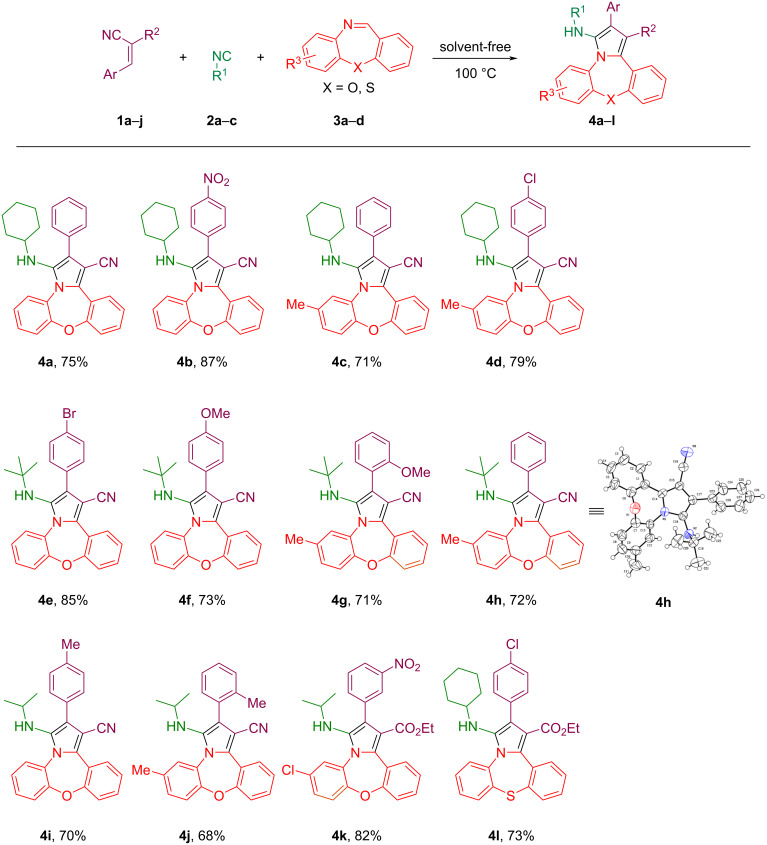
Substrate scope. Conditions: Reactions were carried out using **1** (0.55 mmol), **2** (0.55 mmol), and **3** (0.50 mmol) under solvent-free conditions, stirring in an oil bath at 100 °C for 2 h (monitored by TLC).

**Scheme 4:**
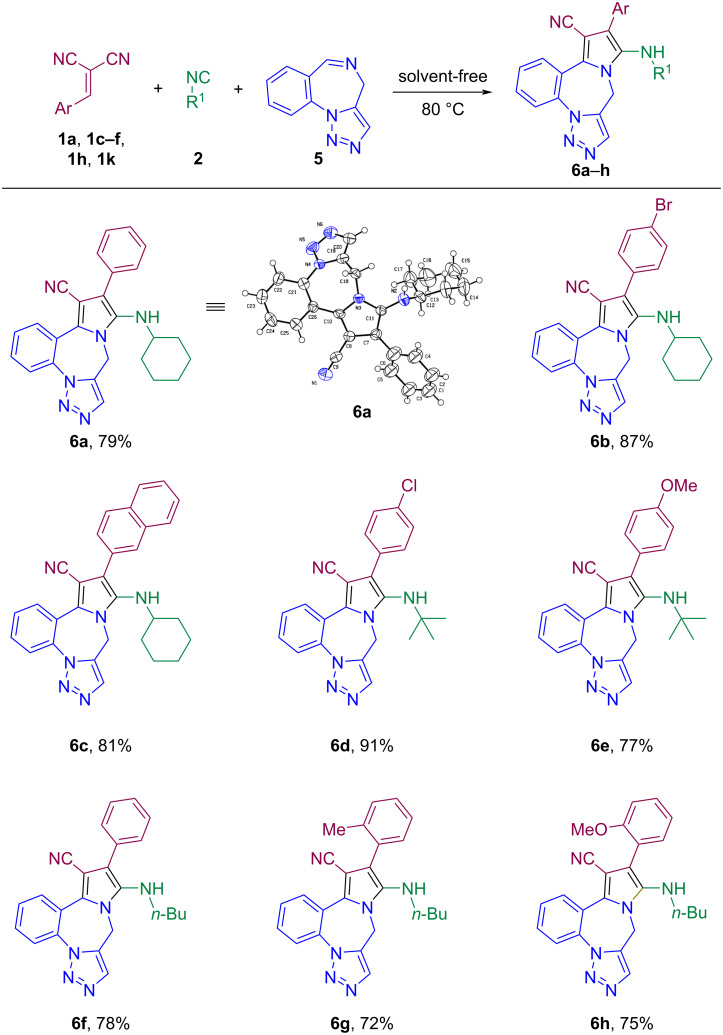
Substrate scope.^.^Conditions: reactions were carried out using **1** (0.55 mmol), **2** (0.55 mmol), and **5** (0.50 mmol) under solvent-free conditions, stirring in an oil bath at 80 °C for 2 h (monitored by TLC).

After having identified the optimal conditions, we next studied the scope of this reaction with substituted benzoxazepines, *gem*-diactivated olefins, and isocyanide derivatives (Scheme 3). As illustrated in Scheme 3, both electron-donating (-Me, -OMe) and electron-withdrawing (-NO_2_, Cl, and Br) groups were well tolerated under the optimal reaction conditions giving the products **4** in yields ranging from 68% to 87% (Scheme 3, **4a**–**l**). In this reaction, the presence of electron-withdrawing substituents in the aromatic rings of the *gem*-diactivated olefins led to slightly better yields of the products **4** when compared to the substrates having electron-donating substituents (Scheme 3). The cause of this phenomenon is probably related to the electron-widthdawing effect of these substitution groups in olefin, which affects the nucleophilic attack of the isocyanides. When a carboxylate substituent was present instead of the carbonitrile in the *gem*-diactivated olefins, the desired products were also obtained in good yields (Scheme 3, **4k** and **4l**). On the other hand, the presence of electron-donating and electron-withdrawing substitutions in the dibenzoxazepine core were also investigated and it was observed, that electron-withdrawing substituent led to an increase and the electron-donating substituents led to a decrease in the reaction efficiency (Scheme 3, **4a**–**k**). In addition, benzothiazepine was used in this protocol and the corresponding pyrrole-fused benzothiazepine was obtained with a yield of 73% (Scheme 3, **4l**). Also, various isocyanides were suitable for this reaction. By replacing cyclohexyl isocyanide with *tert*-butyl- and isopropyl isocyanide, the corresponding products were obtained with similar yields.

To investigate the reactivity of other cyclic imines in this protocol, we performed the reaction of triazolobenzodiazepine with *gem*-diactivated olefins and isocyanides (Scheme 4). As expected, under almost the same conditions as described in Scheme 3 (only at 80 °C), a new type of heterocyclic compounds, pyrrole-fused triazolobenzodiazepines, was obtained in high yield. As summarized in Scheme 4, a variety of *gem*-diactivated olefins with electron-donating (-Me, -OMe), electron-withdrawing (-NO_2_), and halogen (-Cl and -Br) substitutions on the aromatic ring were well tolerated under the reaction conditions. Pyrrole-fused triazolobenzodiazepines **6** were obtained in yields ranging from 72% to 91% (Scheme 4, **6a–h**). Similar to the reaction of benzoxazepine, the presence of an electron-withdrawing group leads to an increase in the yield of pyrrole-fused triazolobenzodiazepines whereas electron-donating groups led to a decrease in its yield. Also, the substitution of naphthyl used in *gem*-diactivated olefins increased the efficiency of the desired product compared to the substitution of phenyl (Scheme 4, **6c**). Furthermore, *n*-butyl isocyanide was used to increase the variety of products and the *n*-butyl-substituted products **6f–h** were obtained with 72–78% yield .

**Figure 2:**
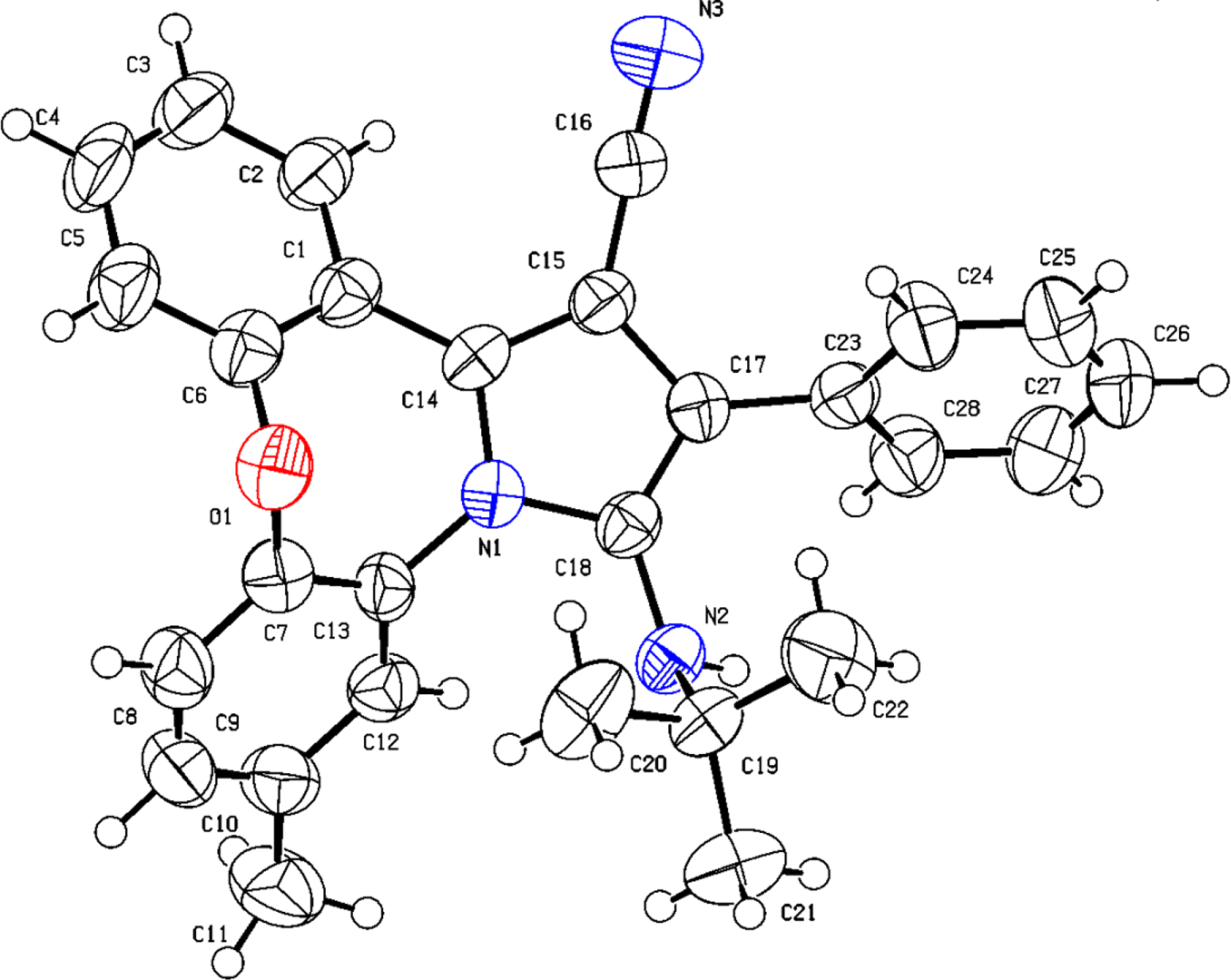
The crystal structure of **4h** (CCDC 2365305).

All the products were characterized by ^1^H NMR, ^13^C NMR, and infrared spectroscopy, and mass spectrometry. Taking **4h** as an example for the analysis of its structure, in its ^1^H NMR spectrum, one singlet signal appears at δ = 0.67 (9H) corresponding to the three methyl groups in the *tert*-butyl substituent. A singlet at δ = 2.39 (3H) is assigned to the protons of the CH_3_-group on the phenyl. The signal at δ = 3.42 is the NH group. All the protons of the aromatic rings are located from δ = 7.10 to 7.99. In its ^13^C NMR spectrum, all of the carbon signals appear at δ = 158.3, 152.5, 134.7, 134.2, 133.9, 133.2, 130.8, 130.4, 129.2, 129.1, 129.0 128.9, 128.4, 127.4, 125.8, 122.6, 121.1, 120.6, 117.1 (C_Ar_), 91.1 (CN), 56.8, 29.4, 20.9 (C_Aliphatic_), respectively. In its mass spectrum, the calculated value matched the found value *m*/*z* 419 calculated for [M]^+^ C_28_H_25_N_3_O. In the IR spectrum, the absorption peak index in 2213 is related to the CN group. For final confirmation, the derivative **4h** was studied by X-ray diffraction analysis, and the crystal structure is illustrated in Figure 2 (detailed information can be found in the Supporting Information File 1).

**Figure 3:**
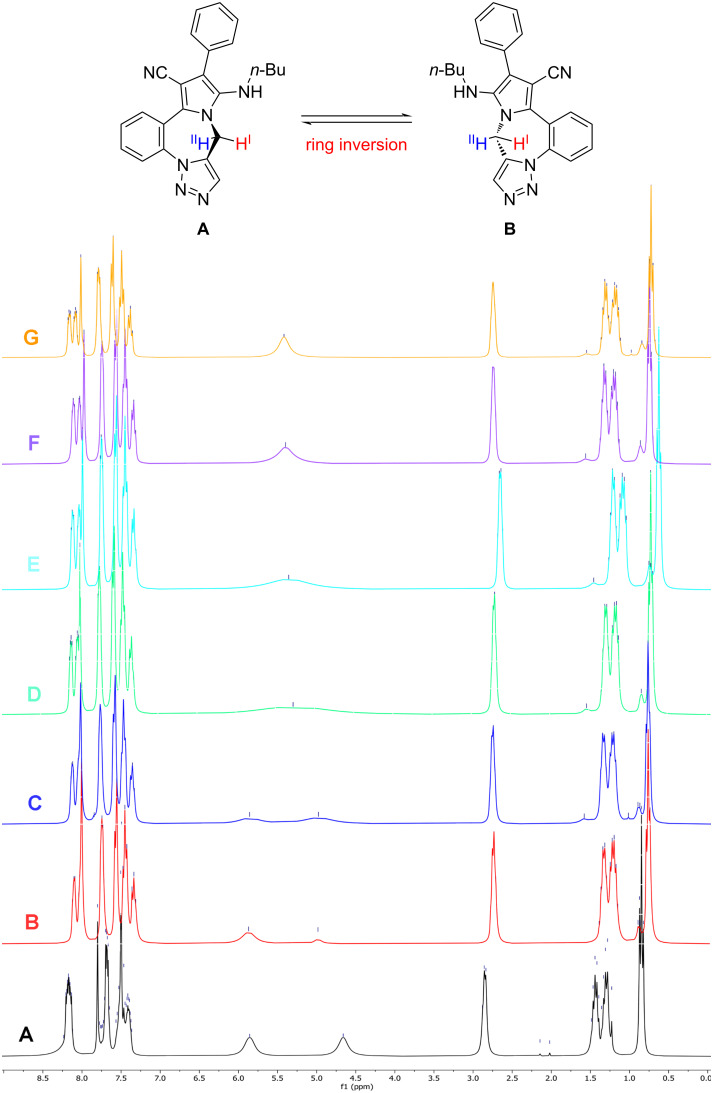
The DNMR (dynamic nuclear magnetic resonance) spectra of compound **6f** (DMSO-*d*_6_, 300 MHz) at 25–85 °C; spectrum A: 25 °C, spectrum B: 35 °C, spectrum C: 45 °C, spectrum D: 55 °C, spectrum E: 65 °C, spectrum F: 75 °C and spectrum E: 85 °C.

The ^1^H NMR spectrum of product **6f** obtained through the I-MCR was investigated and some unexpected chemical shifts were observed at room temperature (Figure 3A) [46]. Therefore, dynamic NMR measurements were performed for compound **6f** at various temperatures (25, 35, 45, 55, 65, 75, and 85 °C). As illustrated in Figure 3, all peaks in the spectrum correspond to the structure of **6f**. Spectrum A recoreded at 25 °C has two broad singulet signals at δ = 4.65 and δ = 5.84 ppm corresponding to hydrogen I and hydrogen II. Remarkably, at higher temperatures (85 °C), the rapid inversion of the seven-membered ring results in it being observed as a single structure on the ^1^H NMR time scale (see Figure 3, spectrum F) [19,47–48].

**Figure 4:**
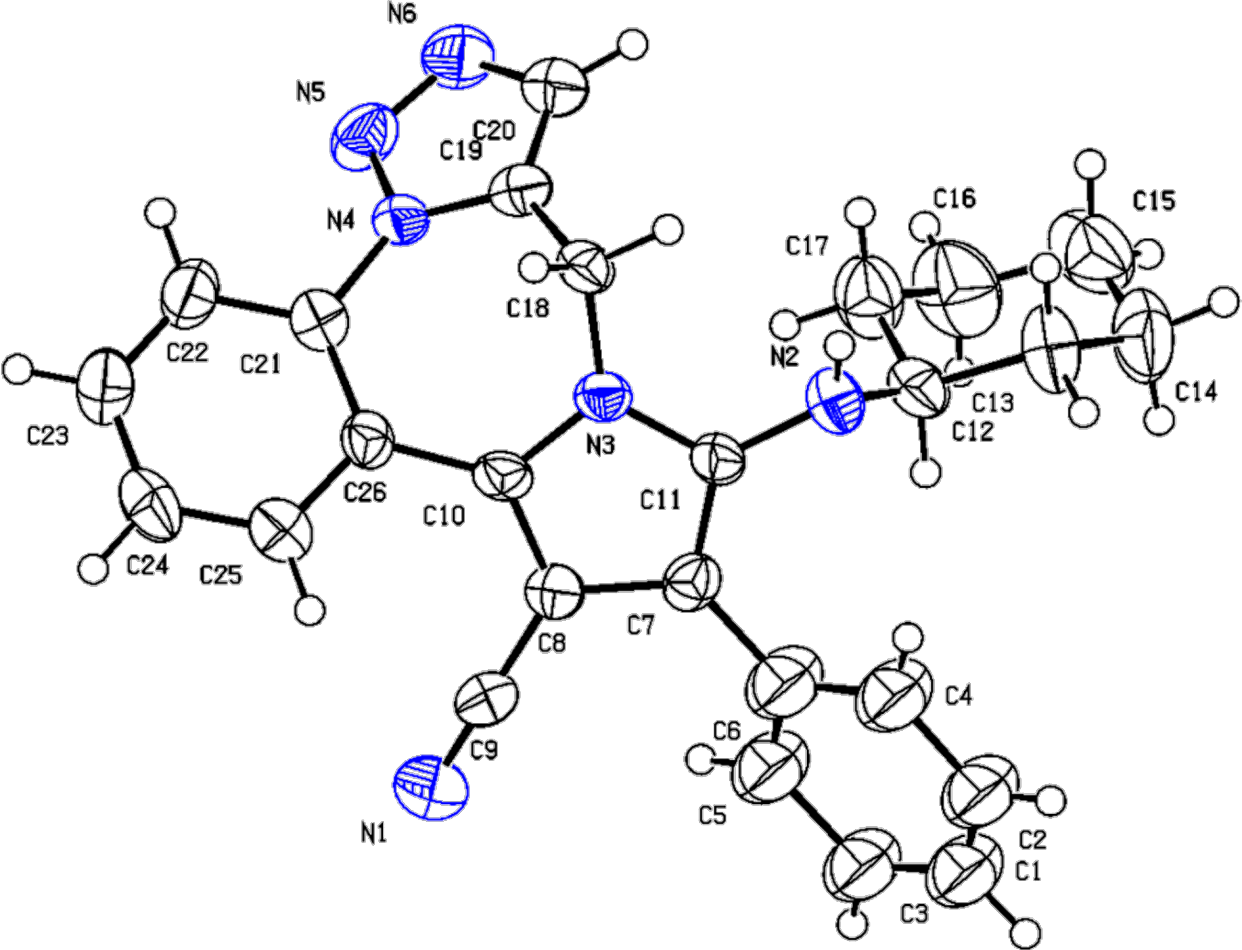
The crystal structure of **6a** (CCDC2365306).

Furthermore, ^13^C NMR analysis, mass spectrum, and IR are consistent with the structure (for details see Supporting Information File 1). Finally, a single crystal X-ray analysis of compound **6a** was performed, confirming the structure (Figure 4).

**Scheme 5:**
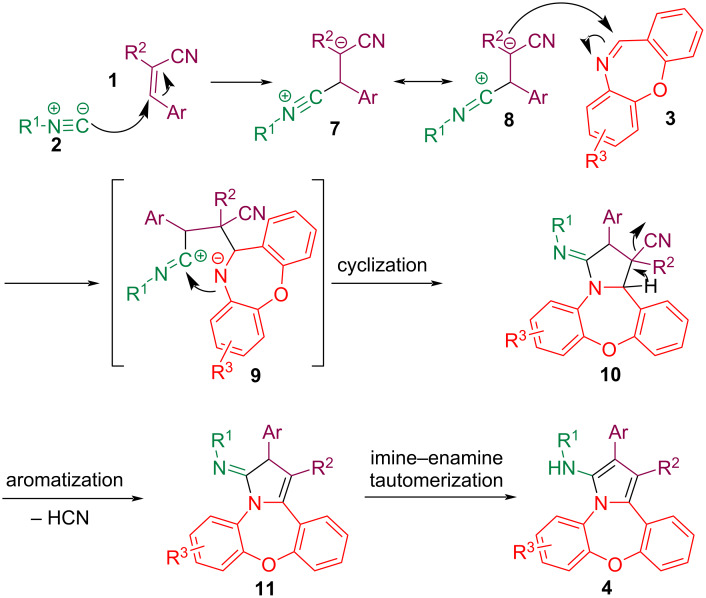
A suggested mechanism for compounds **4**.

The proposed mechanism for an isocyanide-based multicomponent domino reaction for synthesizing pyrrole-fused dibenzoxazepine is illustrated in Scheme 5. The reaction is initiated by the nucleophilic attack of the isocyanide **2** on the *gem*-diactivated olefin **1** to give the zwitterion intermediate **7**. The reaction proceeds with the nucleophilic attack of the zwitterion intermediate **8** on the cyclic imine **3** until intermediate **9** is formed. Then, with the cyclization process intermediate **10** is obtained. Finally, pyrrole-fused benzoxazepine is synthesized by successive processes involving the loss of HCN and the tautomeric enamine imine formation.

**Scheme 6:**
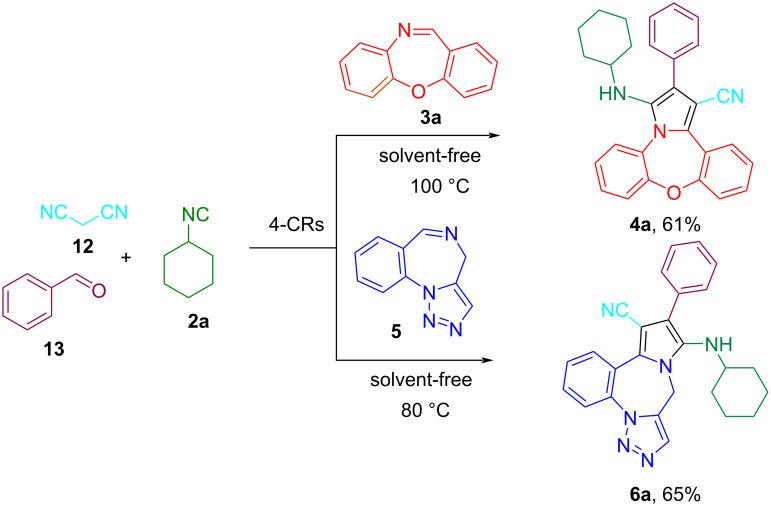
Synthesis of pyrrole-fused dibenzoxazepine/triazolobenzodiazepine through a 4-CR.

The successful synthesis of pyrrole-fused benzoxazepine/triazolobenzodiazepine derivatives via a 3-CR prompted us to investigate the synthesis of these compounds as a 4-CR. Fortunately, the pyrrole-fused benzoxazepine/triazolobenzodiazepines **4a** and **6a** could also be obtianed through a one-pot 4-CR from benzaldehyde, malononitrile, cyclohexyl isocyanide, and benzoxazepine/triazolobenzodiazepine imine (Scheme 6). However, the yield of the products based on the four-component method was lower compared to those obtained via the three-component strategy.

**Scheme 7:**
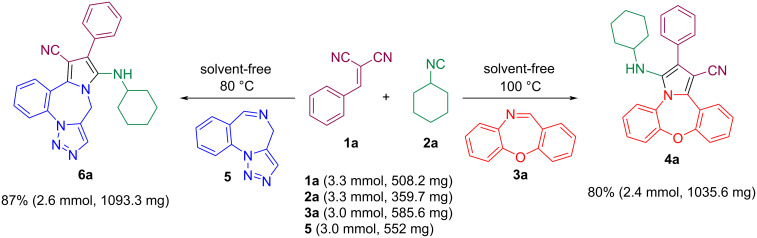
Gram-scale synthesis of pyrrole-fused dibenzoxazepine/triazolobenzodiazepine **4a** and **6a** via 3-CRs.

After the efficient and straightforward synthesis of the pyrrole-fused dibenzoxazepine/triazolobenzodiazepine derivatives on a submillimolar scale, two reactions were conducted on a gram scale to validate the protocol's efficacy (Scheme 7). Reacting each of the cyclic imines at a 3 mmol scale led to the pyrrole-fused dibenzoxazepine/triazolobenzodiazepine **4a** and **6a** in 80 and 87% yields, respectively. In general, a significant increase in the yield of the products was observed compared to the submillimole state.

### Physical properties

**Figure 5:**
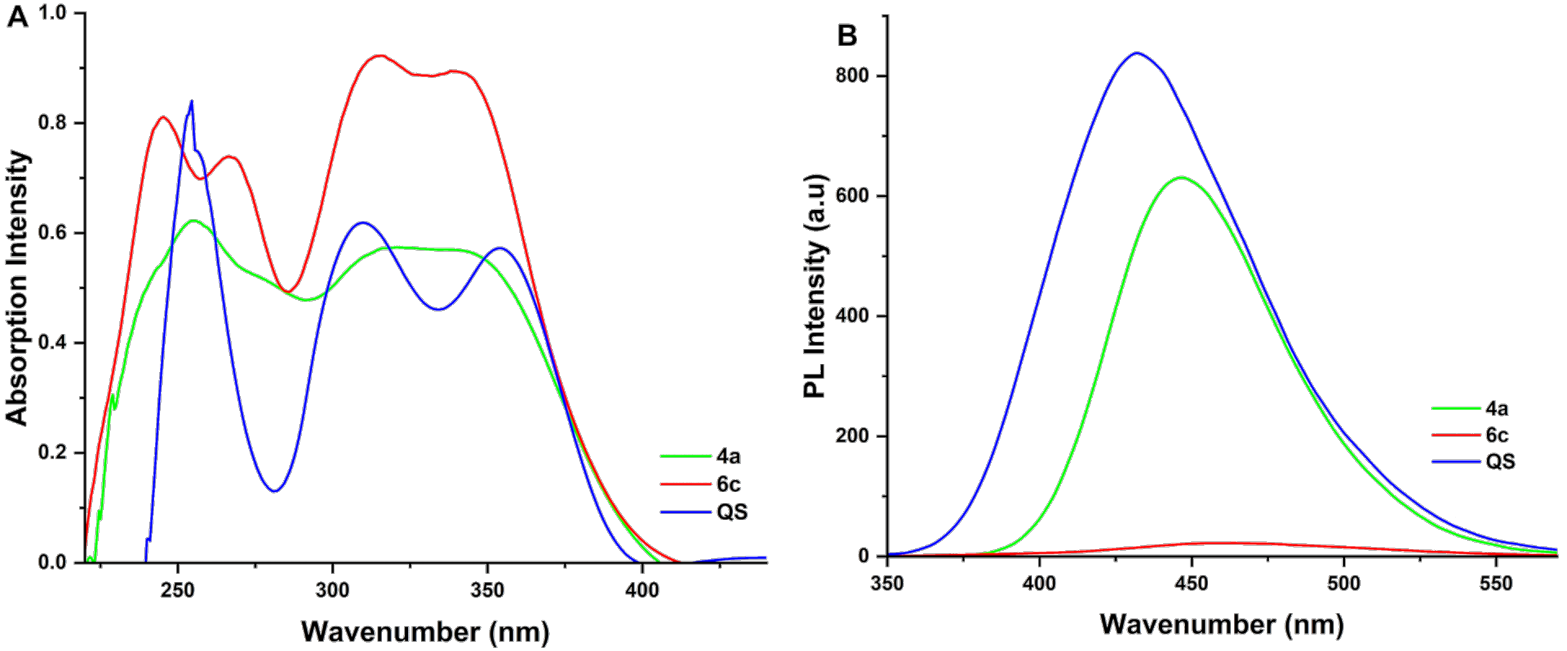
UV–vis absorption for compounds **4a**, **6c** and QS (quinine sulfate) (a); emission for **4a**, **6c** and QS (b); *c* = 75 ppm in ethanol and *T* = 298 K.

The compounds such as benzoxazepines, benzothiazepines, benzodiazepines, and pyrrole have shown promising applications in optoelectronics, biophotonics, and cell imaging due to their suitable fluorophores [49–51]. The UV–vis absorption and emission spectra of products **4** and **6** were studied in ethanol at 298 K. The absorption range (λ_abs_) was 400–225 nm, and the emission range (λ_em_) was 560–400 nm. These findings are shown in Figure S3 in Supporting Information File 1. An interesting trend for the absorption of the synthesized derivatives **4** and **6** was observed when electron-donating substituents were present in the phenyl ring, which led to an increase in the intensity of the absorption and emission wavelengths (Supporting Information File 1, Figure S3). As is evident in numerous reports, quantum yield is one of the most important photophysical parameters for describing luminescent molecules and materials since high quantum efficiency is important for a wide range of applications, including displays, lasers, bio-imaging, solar cells, and accurate measurement of the quantum yield is therefore important [52–54]. Therefore, the quantum yield was determined using the standard of quinine sulfate for all the products (**4a–l** and **6a–h**). Products **4a** and **6c**, which had the highest intensity of absorption and emission from derivatives of benzoxazepines and triazolobenzodiazepines, respectively, their quantum yield was calculated and **4a** was obtained with 48.35% and **6c** with 1.04% (Figure 5A and 5B) [55]. According to the results obtained from the quantum yield, we can claim that these synthesized derivatives pyrrole-fused dibenzoxazepine/dibenzothiazepine/triazolobenzodiazepine derivatives could serve as potential candidates for optoelectronic conjugate materials [56].

## Conclusion

In summary, we have designed a simple and novel procedure for synthesizing pyrrole-fused dibenzoxazepine/dibenzothiazepine/triazolobenzodiazepine derivatives in high yields. This one-pot 3-CR includes isocyanides, *gem*-diactivated olefins, and cyclic imines (dibenzoxazepines, dibenzothiazepine, and triazolobenzodiazepine) under catalyst- and solvent-free conditions. Furthermore, the other advantages of this reaction include the manufacturing premium pharmaceutical scaffolds, a wide range of substrates, short reaction times, and simple operation. According to quantum efficiency calculations, all pyrrole-fused dibenzoxazepine/dibenzothiazepine/triazolobenzodiazepine derivatives with excellent emission could serve as potential candidates for optoelectronic conjugate materials.

## Experimental

### General information

All commercially available reagents and chemicals were bought from Merck & Co. and utilized without extra purification. The melting points of all synthesized compounds were measured utilizing an Electrothermal 9200 apparatus. ^1^H and ^13^C NMR and spectra in CDCl_3_ and DMSO-*d*_6_ solvents were recorded on a Bruker Avance spectrometer at 300.13 MHz and 75.47 MHz, respectively. IR spectra were created on a Thermo Nicolet Nexus 470 FT-IR spectrometer in cm^−1^. Mass spectra with an HP (Agile Technologies) 5975C Mass Selective Detector were used to confirm the mass of the synthesized products. The PL spectra of the products were obtained using an LS45 spectrometer from PerkinElmer. Elemental analyses were conducted using an Vario El CHN mode system from Elementar GmbH. The PL and the UV–vis spectra were obtained using a spectrofluorometer (LS45, PerkinElmer) and a Specord S 600 (Analytik Jena), respectively.

### Preventive education for synthesizing triazolobenzodiazepine **6a–h**

Azides are highly reactive, toxic, explosive, and shock-sensitive chemicals that can be used under certain conditions. Special safety procedures must be followed during preparation, storage, handling, and disposal. TMSN_3_ is an organic azide that is very sensitive to external factors such as light, heat, friction, and pressure and should be stored in amber plastic containers without light and at a temperature below zero degree Celsius. Exposure to azide occurs through skin absorption, inhalation, or ingestion through the respiratory tract. Which leads to skin and eye irritation, blurred vision, dizziness, weakness/fatigue, hypotension, seizures, and respiratory failure. The following instructions are required to work with TMSN_3_. It is necessary to have a silver shield apron, breathing mask, safety glasses, lab coat, and gloves with high chemical resistance. The reactions should be carried out in a hood with a strong suction and with a protective explosion shield, which in this test should be as low as possible. Since exposure to water and strong acids leads to the formation of hydrazoic acid, which is very toxic, volatile, and explosive, the reaction containers must be completely dry and clean. When consuming TMSN_3_, it should be done cold (below zero degree Celsius) in a container insulated from light. The reaction must be completely closed under the hood and the lid of the reaction container, and the product purification process must be carried out under the mentioned personal protective equipment. The reaction on a larger scale is carried out under special safety conditions [57–59].

**3-(Cyclohexylamino)-2-phenyldibenzo[*b,f*]pyrrolo[1,2-*d*][1,4]oxazepine-1-carbonitrile (4a); ethyl 2-(4-chlorophenyl)-3-(cyclohexylamino)dibenzo[*b,f*]pyrrolo[1,2-*d*][1,4]thiazepine-1-carboxylate (4l) and 11-(cyclohexylamino)-12-phenyl-9*H*-benzo[*f*]pyrrolo[1,2-*d*][1,2,3]triazolo[1,5-*a*][1,4]diazepine-13-carbonitrile (6a); typical procedure:** Cyclic imines of dibenzo[*b*,*f*][1,4]oxazepine **3a–c**, dibenzo[*b*,*f*][1,4]thiazepine **3d**, and 4*H*-benzo[*f*][1,2,3]triazolo[1,5-*a*][1,4]diazepine **5** were prepared according to the procedure in the previous report [44–45] and 0.50 mmol (**3a**, 98 mg; **3d**, 105 mg; **5**, 92 mg) of each was added separately along with 2-benzylidenemalononitrile (**1a**, 0.55 mmol, 85 mg), cyclohexyl isocyanide (**2a**, 0.55 mmol, 60 mg). The reaction mixture was stirred at temperatures of 100 or 80 °C for 2 h using a magnetic stirrer. After making sure that the reactions were completed (monitored by TLC), the reaction mixture was cooled to ambient temperature. Then, the crude mixture was purified using a silica gel chromatography column and washed with *n*-hexane and ethyl acetate solvent mixtures (1:3 for **3a** and **3d**; 2:1 for compounds **5**).

## Supporting Information

File 1 General synthetic procedures and characterization and copies of ^1^H NMR, ^13^C NMR, FTIR and mass spectra.

## Data Availability

Data generated and analyzed during this study is available from the corresponding author upon reasonable request.
