# Supplementary material for: Synthesis of pyrrole-fused dibenzoxazepine/dibenzothiazepine/triazolobenzodiazepine derivatives via isocyanide-based multicomponent reactions
Source: Beilstein J Org Chem. 2024 Nov 11;20:2870–82. doi: 10.3762/bjoc.20.241 (PMC11571948; doi:10.3762/bjoc.20.241)
Supplement: File 1 — General synthetic procedures and characterization and copies of 1H NMR, 13C NMR, FTIR and mass spectra. [file Beilstein_J_Org_Chem-20-2870-s001.pdf]

BEILSTEIN JOURNAL OF ORGANIC CHEMISTRY

Supporting Information

for

Synthesis of pyrrole-fused dibenzoxazepine/ dibenzothiazepine/triazolobenzodiazepine derivatives via isocyanide-based multicomponent reactions

Marzieh Norouzi, Mohammad Taghi Nazeri, Ahmad Shaabani and Behrouz Notash

Beilstein J. Org. Chem. **2024,** *20,* 2870–2882. doi:10.3762/bjoc.20.241

General synthetic procedures and characterization and copies of ^{1}H NMR, ^{13}C NMR, FTIR and mass spectra

License and Terms: This is a supporting information file under the terms of the Creative Commons Attribution License (https://creativecommons.org/licenses/by/4.0). Please note that the reuse, redistribution and reproduction in particular requires that the author(s) and source are credited and that individual graphics may be subject to special legal provisions.
The license is subject to the *Beilstein Journal of Organic Chemistry* terms and conditions: (https://www.beilstein-journals.org/bjoc/terms)

Table of contents

Characterization data S1
X-ray crystallographic information S14
Preparation method of a single crystal of compound **4h** S14
Preparation Method of a Single Crystal of Compound **6a** S15
Photophysical study S17
Fluorescence quantum yield in solution S19
^{1}H NMR, ^{13}C NMR and mass spectra of the compounds S21

Characterization data

Dibenzo[*b*,*f*][1,4]oxazepine (3a)

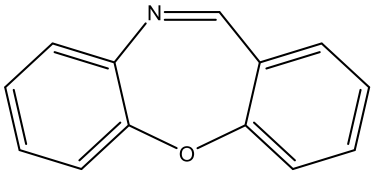

Yellow crystals; yield: 683 mg (70%); mp 69-71 °C. ^{1}H NMR (300 MHz, $CDCl_3$) δ 8.55 (s, 1H, H_{imine}), 7.48 (td, J = 7.0, 2.0 Hz, 1H, H_{Ar}), 7.38 (td, J = 7.0, 2.0 Hz, 2H, H_{Ar}), 7.29 – 7.25 (m, 1H, H_{Ar}), 7.24 – 7.19 (m, 2H, H_{Ar}), 7.19 – 7.12 (m, 2H, H_{Ar}). ^{13}C NMR (75 MHz, $CDCl_3$) δ 160.7 (C=N), 160.4, 152.7, 140.4, 133.4, 130.2, 129.2, 128.9, 127.3, 125.7, 125.1, 121.4, 120.8 (C_{Ar}).

8-Methyldibenzo[*b*,*f*][1,4]oxazepine (3b)

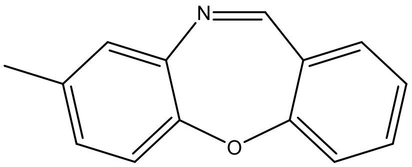

Orange crystals; yield: 753 mg (72%); mp 42-46 °C. ^{1}H NMR (300 MHz, $CDCl_3$) δ 8.53 (s, 1H, H_{imine}), 7.45 (t, J = 7.9 Hz, 1H, H_{Ar}), 7.34 (d, J = 7.7 Hz, 1H, H_{Ar}), 7.24 – 7.11 (m, 3H, H_{Ar}), 7.03 (s, 2H, H_{Ar}), 2.33 (s, 3H, CH_3). ^{13}C NMR (75 MHz, $CDCl_3$) δ 160.6 (C=N), 160.5, 150.5, 140.0, 135.4, 133.3, 130.1, 129.5, 129.4, 127.3, 125.0, 121.0, 120.6 (C_{Ar}), 20.7 (CH_3).

8-Chlorodibenzo[*b,f*][1,4]oxazepine (3c)

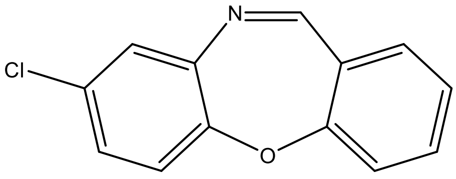

Orange crystals; yield: 858 mg (75%); mp 70-74 °C. ^{1}H NMR (300 MHz, DMSO-d_6) δ 8.62 (s, 1H, H_{imine}), 7.64 – 7.48 (m, 2H, H_{Ar}), 7.41 – 7.26 (m, 3H, H_{Ar}), 7.27 – 7.17 (m, 2H, H_{Ar}). ^{13}C NMR (75 MHz, DMSO-d_6) δ 162.5 (C=N), 159.4, 151.0, 141.4, 134.2, 130.8, 129.6, 128.5, 127.9, 126.7, 125.8, 123.0, 120.5 (C_{Ar}).

Dibenzo[*b,f*][1,4]thiazepine (3d)

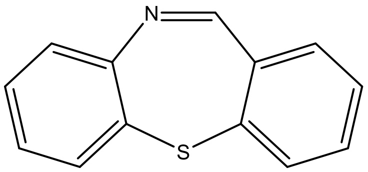

Yellow solid; yield: 739 mg (70%); mp 106-107 °C. ^{1}H NMR (300 MHz, $CDCl_3$) δ 8.92 (s, 1H, H_{imine}), 7.48 – 7.43 (m, 3H, H_{Ar}), 7.42 – 7.36 (m, 3H, H_{Ar}), 7.36 – 7.33 (m, 2H, H_{Ar}). ^{13}C NMR (75 MHz, $CDCl_3$) δ 163.0 (C=N), 159.1, 138.2, 136.5, 134.9, 133.9, 133.6, 130.5, 130.1, 129.8, 126.8, 126.1, 125.0 (C_{Ar}).

4*H*-Benzo[*f*][1,2,3]triazolo[1,5-*a*][1,4]diazepine (5)

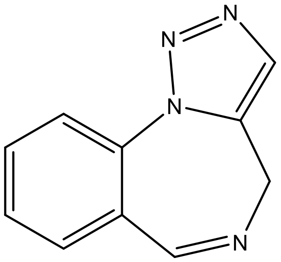

Brownish solid; yield: 1.12 g (61%). mp 147-149 °C. ^{1}H NMR (300 MHz, DMSO-d_6) δ 8.53 (s, 1H, H_{imine}), 8.12 (d, J = 8.0 Hz, 1H, H_{Ar}), 7.86 (s, 1H, H_{Ar}), 7.84 – 7.74 (m, 2H, H_{Ar}), 7.67 (t, J = 7.5 Hz, 1H, H_{Ar}), 4.73 (s, 2H, CH_2). ^{13}C NMR (75 MHz, DMSO-d_6) δ 162.9 (C=N), 136.8, 134.0, 132.5, 131.6, 131.5, 128.6, 124.8, 121.8 (C_{Ar}), 42.7 (CH_2).

3-(Cyclohexylamino)-2-phenyldibenzo[*b,f*]pyrrolo[1,2-*d*][1,4]oxazepine-1-carbonitrile (4a)

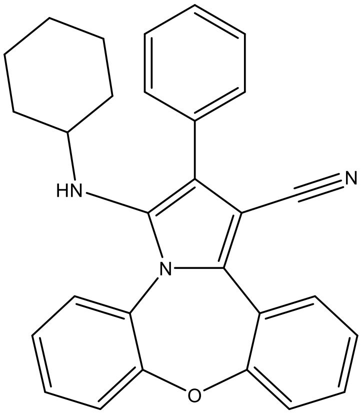

Yellowish orange powder; yield: 161 mg (75%); mp 87-89 °C; R_f (*n*-hexane/EtOAc 2:1) 0.70. Found: C, 80.70; H, 5.81; N, 9.70. $C_{29}H_{25}N_3O$ requires C, 80.72; H, 5.84; N, 9.74. (ATR) ν (cm^{-1}) 3357 (NH), 2932 (CH), 2854 (CH), 2217 (C≡N), 1497 (C=C) cm^{-1}. ^{1}H NMR (300 MHz, $CDCl_3$) δ 7.99 (t, J = 7.9 Hz, 2H, H_{Ar}), 7.69 – 7.46 (m,3H, H_{Ar}), 7.46 – 7.32 (m, 4H, H_{Ar}), 7.31 – 7.10 (m,3H, H_{Ar}), 3.59 (s, 1H, NH), 2.61 – 2.28 (m, 1H, $H_{cyclohexyl}$), 1.74 – 1.35 (m, 5H, $H_{cyclohexyl}$), 1.12 – 0.74 (m, 5H, $H_{cyclohexyl}$).^{13}C NMR (75 MHz, $CDCl_3$) δ 157.6, 153.7, 136.3, 132.8, 132.4, 130.4, 130.1, 129.0, 128.9, 128.8, 128.7, 127.3, 126.2,

126.0, 125.4, 124.8, 122.5, 122.1, 120.7, 117.2, 115.9 (C_{Ar}), 91.548 (CN), 56.2, 33.5, 33.3, 25.5, 24.8, 24.4 ($C_{Aliphatic}$). MS m/z (EI, 70 eV) 431(M^+, 58.40), 348 (31.71), 321 (10.03), 221 (9.54), 180 (9.56), 139 (8.09), 105 (19.94), 83 (32.24), 55 (100).

3-(Cyclohexylamino)-2-(4-nitrophenyl)dibenzo[*b,f*]pyrrolo[1,2-*d*][1,4]oxazepine-1-carbonitrile (4b)

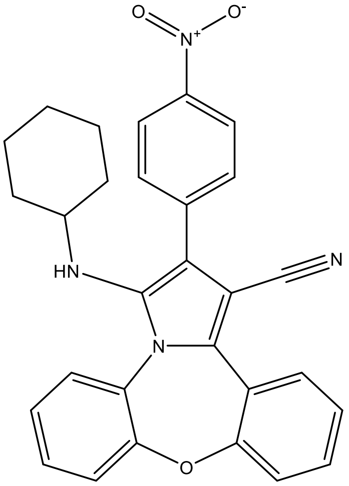

Orange solid; yield: 207 mg (87%); mp 242-245 °C; R_f (*n*-hexane/EtOAc 2:1) 0.56. Found: C, 73.04; H, 5.08; N, 11.73. $C_{29}H_{24}N_4O_3$ requires C, 73.09; H, 5.08; N, 11.76. (ATR) ν (cm^{-1}) 3387 (NH), 2919 (CH), 2852 (CH), 2216 (C≡N), 1462 (C=C) cm^{-1}. ^{1}H NMR (300 MHz, $CDCl_3$) δ 8.38 (d, *J* = 8.4 Hz, 1H, H_{Ar}), 7.96 (d, *J* = 7.7 Hz, 1H, H_{Ar}), 7.88 (d, *J* = 8.0 Hz, 1H, H_{Ar}), 7.78 (d, *J* = 8.4 Hz, 1H, H_{Ar}), 7.55 – 7.16 (m, 6H, H_{Ar}), 5.15 (s, 1H,NH) 2.51 (bs, 1H, $H_{cyclohexyl}$), 1.66 (bs, 2H, $H_{cyclohexyl}$), 1.34 – 1.29 (m, 4H, $H_{cyclohexyl}$), 0.93 – 0.85 (m, 4H, $H_{cyclohexyl}$). ^{13}C NMR (75 MHz, $CDCl_3$) δ 157.8, 154.1, 146.4, 139.6, 137.0, 134.0, 130.7, 129.7, 129.2, 129.1, 128.9, 126.2, 125.8, 125.6, 124.3, 122.4, 122.0, 120.8, 116.6, 113.1 (C_{Ar}), 91.0 (CN), 56.4, 33.7, 33.3, 25.3, 24.7, 24.4 ($C_{Aliphatic}$). MS m/z (EI, 70 eV) 476 (M^+, 58.02), 394 (35.65), 377 (13.60), 346 (29.69), 221 (13.95), 180 (16.05), 83 (34.85), 55 (100).

3-(Cyclohexylamino)-6-methyl-2-phenyldibenzo[*b,f*]pyrrolo[1,2-*d*][1,4]oxazepine-1-carbonitrile (4c)

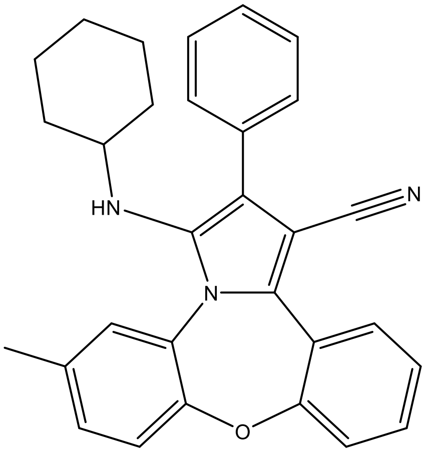

Reddish brown oil; yield: 158 mg (71%); mp 82-83 °C; R_f (*n*-hexane/EtOAc 2:1) 0.76. Found: C, 80.81; H, 6.07; N, 9.40. $C_{30}H_{27}N_3O$ requires C, 80.87; H, 6.11; N, 9.43. (ATR) ν (cm^{-1}) 3355 (NH), 2928 (CH), 2854 (CH), 2216 (C≡N), 1453 (C=C) cm^{-1}. ^{1}H NMR (300 MHz, $CDCl_3$) δ 7.95 (d, *J* = 7.3 Hz, 1H, H_{Ar}), 7.82 (s, 1H, H_{Ar}), 7.52 (s, 4H, H_{Ar}), 7.42 – 7.19 (m, 5H, H_{Ar}), 7.12 (d, *J* = 8.5 Hz, 1H, H_{Ar}), 3.61 (s, 1H, NH), 2.47 (bs, 1H. $H_{cyclohexyl}$), 2.36 (s, 3H, CH_3), 1.62 (bs, 4H, $H_{cyclohexyl}$), 1.41 (bs, 2H, $H_{cyclohexyl}$), 0.95 (bs, 4H, $H_{cyclohexyl}$). ^{13}C NMR (75 MHz, $CDCl_3$) δ 157.8, 151.6, 136.2, 135.2, 132.8, 132.4, 130.0, 129.9, 129.1, 129.0, 128.9, 127.2,

126.3, 125.9, 122.5, 121.6, 120.6, 117.2, 115.6 (C_{Ar}), 91.8 (CN), 53.8, 33.5, 33.3, 25.5, 24.8, 24.4, 20.9 ($C_{Aliphatic}$). MS m/z (EI, 70 eV) 445 (M^+, 79.78), 362 (35.22), 335 (8.93), 235 (7.19), 221 (8.52), 194 (8.43), 149 (8.03), 98 (12.74), 83 (34.03), 55 (100).

2-(4-Chlorophenyl)-3-(cyclohexylamino)-6-methyldibenzo[*b,f*]pyrrolo[1,2-*d*][1,4]oxazepine-1-carbonitrile (4d)

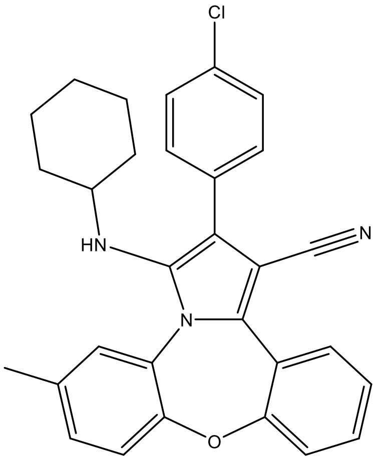

Light lime green powder; yield: 189 mg (79%); mp 190-192 °C; R$_f$ (*n*-hexane/EtOAc 2:1) 0.70. Found: C, 75.01; H, 5.45; N, 8.72. $C_{30}H_{26}ClN_3O$ requires C, 75.07; H, 5.46; N, 8.75. (ATR) ν (cm^{-1}) 3378 (NH), 2925 (CH), 2852 (CH), 2219 (C≡N), 1456 (C=C) cm^{-1}. ^{1}H NMR (300 MHz, $CDCl_3$) δ 7.94 (d, *J* = 7.7 Hz, 1H, H_{Ar}), 7.77 (s, 1H, H_{Ar}), 7.55 – 7.39 (m, 3H, H_{Ar}), 7.38 – 7.21 (m, 3H, H_{Ar}), 7.14 (d, *J* = 8.4 Hz, 1H, H_{Ar}), 3.53 (s, 1H, NH), 2.53 – 2.43 (m, 1H, $H_{cyclohexyl}$), 2.37 (s, 3H, CH_3), 1.53 – 1.36 (m, 3H, $H_{cyclohexyl}$), 1.34 – 1.20 (m, 2H, $H_{cyclohexyl}$), 1.10 – 0.88 (m, 5H, $H_{cyclohexyl}$). ^{13}C NMR (75 MHz, $CDCl_3$) δ 157.9, 151.7, 136.8, 135.3, 133.1, 133.1, 131.0, 130.2, 130.2, 129.65, 129.3, 129.2, 128.8, 126.2, 125.9, 122.4, 121.7, 120.6, 117.1, 114.3 (C_{Ar}), 91.2 (CN), 56.2, 33.7, 33.4, 25.5, 24.8, 24.4, 20.9($C_{Aliphatic}$). MS m/z (EI, 70 eV) 479 (M^+, 30.54), 481 (M^++1, 12.44), 397 (14.56), 361 (8.85), 235 (5.88), 221 (7.25), 194 (8.07), 98 (12.42), 83 (34.82), 55 (100).

2-(4-Bromophenyl)-3-(*tert*-butylamino)dibenzo[*b,f*]pyrrolo[1,2-*d*][1,4]oxazepine-1-carbonitrile (4e)

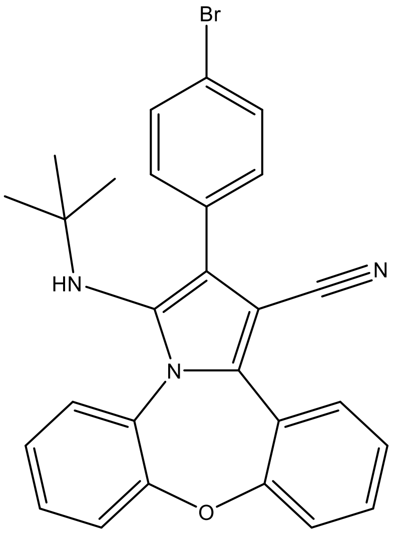

Yellowish orange powder; yield: 205 mg (85%); mp 200-202 °C; R$_f$ (*n*-hexane/EtOAc 2:1) 0.69. Found: C, 66.94; H, 4.56; N, 8.65. $C_{27}H_{22}BrN_3O$ requires C, 66.95; H, 4.58; N, 8.67. (ATR) ν (cm^{-1}) 3408 (NH), 2960 (CH), 2925 (CH), 2854 (CH), 2222 (C≡N), 1462 (C=C) cm^{-1}. ^{1}H NMR (300 MHz, $CDCl_3$) δ 7.94 (d, *J* = 7.7 Hz, 1H, H_{Ar}), 7.84 (d, *J* = 7.9 Hz, 1H, H_{Ar}), 7.64 (s, 1H, H_{Ar}), 7.61 (s, 1H, H_{Ar}), 7.43 (d, *J* = 8.3 Hz, 2H, H_{Ar}), 7.40 – 7.31 (m, 3H, H_{Ar}), 7.30 – 7.21 (m, 2H, H_{Ar}), 3.29 (s, 1H, NH), 0.66 (s, 9H, $H_{\textit{tert-}butyl}$). ^{13}C NMR (75 MHz, $CDCl_3$) δ 158.1, 154.6, 134.1, 134.1, 132.2, 132.1, 131.0, 130.8, 130.7, 129.0, 128.7, 128.0, 126.0,

125.0, 122.3, 121.7, 121.6, 120.7, 116.8 (C_{Ar}), 90.9 (CN), 56.9, 29.4 ($C_{Aliphatic}$). MS m/z (EI, 70 eV) 483 (M^+, 18.68), 485 (M^++1, 15,65), 359 (31.30), 231 (14.64), 97 (17.17), 84 (52.52), 69 (32.82), 55 (100).

3-(*tert*-Butylamino)-2-(4-methoxyphenyl)dibenzo[*b,f*]pyrrolo[1,2-*d*][1,4]oxazepine-1-carbonitrile (4f)

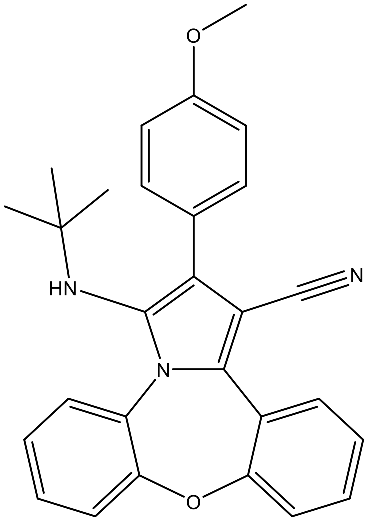

Light green solid; yield: 158 mg (73%); mp 183-185 °C; R_f (*n*-hexane/EtOAc 2:1) 0.60. Found: C, 77.19; H, 5.77; N, 9.64. $C_{28}H_{25}N_3O_2$ requires C, 77.22; H, 5.79; N, 9.65. (ATR) ν (cm^{-1}) 3390 (NH), 2966 (CH), 2928 (CH), 2857 (CH), 2219 (C≡N), 1462 (C=C) cm^{-1}. ^1H NMR (300 MHz, $CDCl_3$) δ 8.03 – 7.93 (dd, *J* = 9, 3 Hz, 1H, H_{Ar}), 7.93 – 7.83 (dd, *J* = 9, 3 Hz 1H, H_{Ar}), 7.50 – 7.41 (m, 2H, H_{Ar}), 7.41 – 7.34 (m, 2H, H_{Ar}), 7.33 – 7.23 (m, 3H, H_{Ar}), 7.06 (s, 1H, H_{Ar}), 7.03 (s, 1H, H_{Ar}), 3.88 (s, 3H, CH_3), 3.36 (s, 1H, NH), 0.66 (s, 9H, $H_{tert\text{-}butyl}$). ^{13}C NMR (75 MHz, $CDCl_3$) δ 158.9, 158.0, 154.5, 134.0, 133.6, 131.3, 130.4, 130.3, 129.0, 128.4, 128.1, 125.9, 125.4, 124.9, 122.6, 122.5, 121.6, 120.7, 117.2, 114.4 (C_{Ar}), 91.3 (CN), 56.7, 55.3, 29.4 ($C_{Aliphatic}$). MS m/z (EI, 70 eV) 435 (M^+, 31.48), 379 (67.62), 221 (11.39), 180 (9.68), 139 (8.50), 77 (7.95), 57 (100).

3-(*tert*-Butylamino)-2-(2-methoxyphenyl)-6-methyldibenzo[*b,f*]pyrrolo[1,2-*d*][1,4] oxazepine-1-carbonitrile (4g)

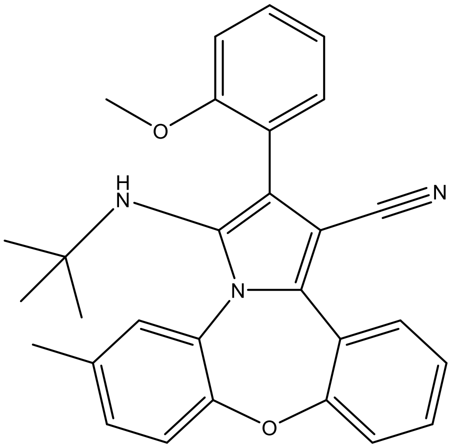

Yellow oil; yield: 159 mg (71%); mp 193-196 °C, R_f (*n*-hexane/EtOAc 2:1) 0.61. Found: C, 77.45; H, 6.01; N, 9.34. $C_{29}H_{27}N_3O_2$ requires C, 77.48; H, 6.05; N, 9.35. (ATR) ν (cm^{-1}) 3346 (NH), 2966 (CH), 2919 (CH), 2857 (CH), 2222 (C≡N), 1468 (C=C) cm^{-1}. ^1H NMR (300 MHz, $CDCl_3$) δ 7.97 (d, *J* = 7.7 Hz, 1H, H_{Ar}), 7.86 (s, 1H, H_{Ar}), 7.54 (d, *J* = 7.6 Hz, 1H, H_{Ar}), 7.42 – 7.30 (m, 3H, H_{Ar}), 7.29 – 7.22 (m, 2H, H_{Ar}), 7.17 – 7.04 (m, 3H, H_{Ar}), 4.01 (s, 3H, OCH_3), 3.46 (s, 1H, NH), 2.40 (s, 3H, CH_3), 0.66 (s, 9H, $H_{tert\text{-}butyl}$). ^{13}C NMR (75 MHz, $CDCl_3$) δ 158.1, 152.4, 135.8, 134.5, 134.2, 133.3, 131.9, 130.8, 130.2, 130.1, 129.1,

129.0, 128.8, 125.8, 125.0, 122.9, 122.1, 121.6, 120.9, 120.5, 117.3, 111.6 (C_{Ar}), 91.5 (CN), 56.2, 56.0, 29.5, 20.9 ($C_{Aliphatic}$). MS m/z (EI, 70 eV) 449 (M^+, 80.24), 393 (58.06), 235 (10.69), 221 (16.44), 194 (7.40), 180 (6.52), 77 (7.10), 57 (100).

3-(*tert*-Butylamino)-6-methyl-2-phenyldibenzo[*b,f*]pyrrolo[1,2-*d*][1,4]oxazepine-1-carbonitrile (4h)

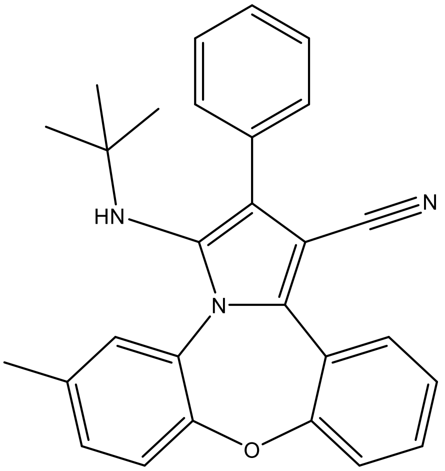

Orange oil (151 mg, 72%); mp 227-229 ˚C; R_f (*n*-hexane/EtOAc 2:1) 0.74. Found: C, 80.10; H, 5.98; N, 10.01. $C_{28}H_{25}N_3O$ requires C, 80.16; H, 6.01; N, 10.02. (ATR) ν (cm^{-1}) 3349 (NH), 2960 (CH), 2925 (CH), 2857 (CH), 2213 (C≡N), 1462 (C=C) cm^{-1}. ^1H NMR (300 MHz, $CDCl_3$) δ 7.97 (dd, *J* = 9, 3 Hz, 1H, H_{Ar}), 7.78 (s, 1H, H_{Ar}), 7.60 – 7.48 (m, 3H, H_{Ar}), 7.44 – 7.33 (m, 2H, H_{Ar}), 7.33 – 7.23 (m, 2H, H_{Ar}), 7.12 (dd, *J* = 9, 3 Hz,1H), 3.42 (s, 1H, NH), 2.39 (s, 3H, CH_3), 0.67 (s, 9H, $H_{tert\text{-}butyl}$). ^{13}C NMR (75 MHz, $CDCl_3$) δ 158.3, 152.5, 134.7, 134.2, 133.9, 133.2, 130.8, 130.4, 129.2, 129.1, 129.0 128.9, 128.4, 127.4, 125.8, 122.6, 121.1, 120.6, 117.1 (C_{Ar}), 91.1 (CN), 56.8, 29.4, 20.9 ($C_{Aliphatic}$). MS m/z (EI, 70 eV) 419 (M^+, 48.38), 363 (66.04), 335 (7.13), 235 (11.39), 221 (11.46), 194 (8.44), 165 (5.16), 105 (4.47), 77 (11.85), 57 (100).

3-(Isopropylamino)-2-(*p*-tolyl)dibenzo[*b,f*]pyrrolo[1,2-*d*][1,4]oxazepine-1-carbonitrile (4i)

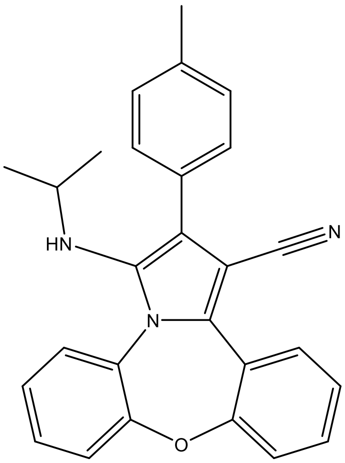

Yellowish white powder; yield: 141 mg (70%); mp 185-186 ˚C; R_f (*n*-hexane/EtOAc 2:1) 0.67. Found: C, 79.95; H, 5.71; N, 10.32. $C_{27}H_{23}N_3O$ requires C, 79.97; H, 5.72; N, 10.36. (ATR) ν (cm^{-1}) 3361 (NH), 2922 (CH), 2216 (C≡N), 1459 (C=C) cm^{-1}. ^1H NMR (300 MHz, $CDCl_3$) δ 8.09 – 7.93 (m, 2H, H_{Ar}), 7.48 – 7.38 (m, 2H, H_{Ar}), 7.38 – 7.21 (m, 7H, H_{Ar}), 3.48 – 3.33 (m, 1H, $H_{isopropyl}$), 2.82 (s, 1H, NH), 2.44 (s, 3H, CH_3), 0.88 (dd, *J* = 6.4, 2.0 Hz, 3H, $H_{isopropyl}$), 0.83 (dd, *J* = 6.4, 2.0 Hz, 3H, $H_{isopropyl}$). ^{13}C NMR (75 MHz, $CDCl_3$) δ 157.6, 153.7, 137.2, 136.3, 132.7, 130.5, 130.1, 129.8, 129.3, 128.8, 128.8, 128.6, 126.1, 126.0, 125.4, 122.5, 122.0, 120.7, 117.2, 116.1 (C_{Ar}), 91.6 (CN), 49.1, 22.9,

21.3 ($C_{Aliphatic}$). MS m/z (EI, 70 eV) 405 (M^+, 100), 362 (52.52), 346 (8.91), 221 (9.24), 180 (8.82), 140 (11.18), 115 (5.56), 91 (6.29), 77 (10.97), 51 (6.27).

3-(Isopropylamino)-6-methyl-2-(*o*-tolyl)dibenzo[*b,f*]pyrrolo[1,2-*d*][1,4]oxazepine-1-carbonitrile (4j)

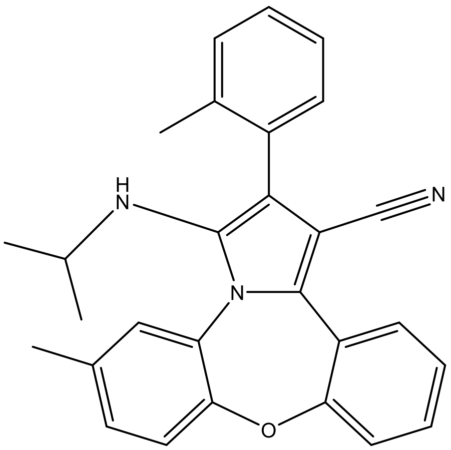

Reddish brown oil; yield: 142 mg (68%); mp 135-138 °C; R_f (*n*-hexane/EtOAc 2:1) 0.69. Found: C, 80.14; H, 5.99; N, 9.97. $C_{28}H_{25}N_3O$ requires C, 80.16; H, 6.01; N, 10.02. (ATR) *ν* (cm^{-1}) 3346 (NH), 2928 (CH), 2216 (C≡N), 1456 (C=C) cm^{-1}. ^1H NMR (300 MHz, $CDCl_3$) *δ* 7.95 (d, *J* = 7.8 Hz, 1H, H_{Ar}), 7.90 (s, 1H, NH), 7.80 (s, 1H, H_{Ar}), 7.51 – 7.22 (m, 8H, H_{Ar}), 7.13 (d, *J* = 8.6 Hz, 1H, H_{Ar}), 2.88 – 2.73 (m, 1H, $H_{isopropyl}$), 2.42 (s, 3H, CH_3), 2.38 (s, 3H, CH_3), 0.88 (d, *J* = 15.0 Hz, 6H, $H_{isopropyl}$). ^{13}C NMR (75 MHz, $CDCl_3$) *δ* 157.6, 151.5, 138.5, 137.6, 136.2, 135.3, 133.2, 132.5, 131.3, 130.7, 130.3, 130.0, 129.1, 128.7, 128.2, 125.9, 125.9, 122.7, 121.7, 120.6, 117.1, 114.1 (C_{Ar}), 92.4 (CN), 48.7, 20.9, 20.1, 14.2 ($C_{Aliphatic}$). MS m/z (EI, 70 eV) 419 (M^+, 100), 376 (30.40), 360 (7.23), 235 (11.43), 220 (16.64), 195 (15.71), 125 (10.44), 111 (16.80), 85 (30.39), 71 (44.34), 57 (82.88).

Ethyl 6-chloro-3-(isopropylamino)-2-(3-nitrophenyl)dibenzo[*b,f*]pyrrolo[1,2-*d*][1,4]oxazepine-1-carboxylate (4k)

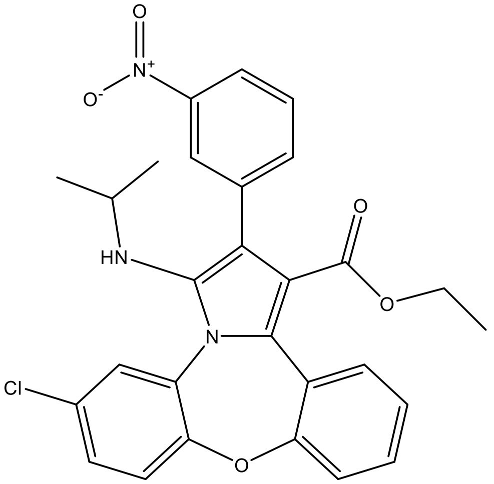

Orange oil ; yield: 212 mg (82%); mp 92-96 °C; R_f (*n*-hexane/EtOAc 2:1) 0.10. Found: C, 64.90; H, 4.66; N, 8.07. $C_{28}H_{24}ClN_3O_5$ requires C, 64.93; H, 4.67; N, 8.11. (ATR) *ν* (cm^{-1}) 3373 (NH), 2925 (CH), 1712 (C=O), 1447 (C=C) cm^{-1}. ^1H NMR (300 MHz, $CDCl_3$) *δ* 8.33 – 8.27 (m, 2H, H_{Ar}), 8.22 (s, 1H, H_{Ar}), 8.15 (s, 1H, H_{Ar}), 7.83 (s, 1H, NH), 7.71 (d, *J* = 5.8 Hz, 3H, H_{Ar}), 7.63 (d, *J* = 4.9 Hz, 3H, H_{Ar}), 7.28 (s, 1H, H_{Ar}), 4.12 (q, *J* = 7.2 Hz, 2H, CH_2), 3.80 – 3.69 (m, 1H, $H_{isopropyl}$), 1.33 (d, *J* = 6.0 Hz, 6H, $H_{isopropyl}$), 1.02 (t, *J* = 7.2 Hz, 3H, CH_3). ^{13}C NMR (75 MHz, $CDCl_3$) *δ* 164.3 (CO), 158.1, 158.1, 156.4, 156.0, 153.4, 148.5, 148.5, 137.4, 135.7, 134.8, 130.5, 130.1, 125.3, 124.7, 124.3,

123.5, 117.4, 116.8 (C_{Ar}), 58.3, 46.4, 23.1, 13.5 ($C_{Aliphatic}$). MS m/z (EI, 70 eV) 517 (M^+, 17.88), 519 (M^++1, 5.67), 472 (11.41), 416 (22.99), 374 (9.54), 154 (13.79), 127 (12.77), 101 (12.60), 84 (100), 59 (24.70).

Ethyl 2-(4-chlorophenyl)-3-(cyclohexylamino)dibenzo[*b*,*f*]pyrrolo[1,2-*d*][1,4]thiazepine-1-carboxylate (4l)

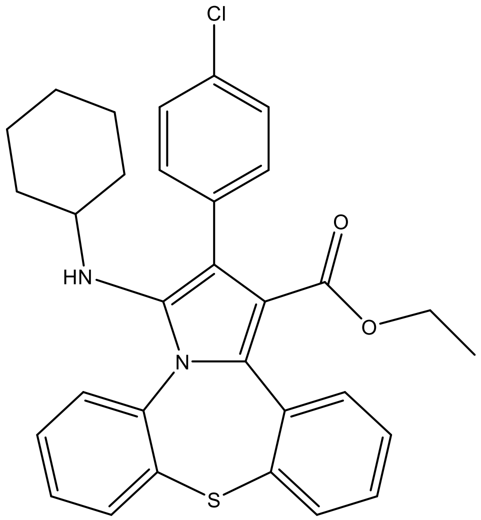

Green powder; yield: 193 mg (73%); mp 158-162 °C; R_f (*n*-hexane/EtOAc 2:1) 0.67. Found: C, 70.35; H, 5.50; N, 5.26; S, 6.01. $C_{31}H_{29}ClN_2O_2S$ requires C, 70.37; H, 5.52; N, 5.29; S, 6.06. (ATR) ν (cm^{-1}) 3364 (NH), 2925 (CH), 2854 (CH), 1709 (C=O), 1442 (C=C) cm^{-1}. ^{1}H NMR (300 MHz, CDCl3) δ 7.85 – 7.65 (m, 2H, H_{Ar}), 7.65 – 7.55 (m, 1H, H_{Ar}), 7.50 – 7.31 (m, 5H, H_{Ar}), 7.29 – 7.13 (m, 3H, H_{Ar}), 4.11 – 3.98 (m, 2H, OCH_2CH_3), 3.31 (bs, 1H, NH), 2.03 (bs, 1H, $H_{cyclohexyl}$), 1.52 (s, 1H, $H_{cyclohexyl}$), 1.43 – 1.29 (m, 4H, $H_{cyclohexyl}$), 0.97 (t, J = 7.1 Hz, 3H, OCH_2CH_3), 0.92 – 0.80 (m, 3H, $H_{cyclohexyl}$), 0.70 (d, J = 9.3 Hz, 1H, $H_{cyclohexyl}$), 0.58 (d, J = 10.8 Hz, 1H, $H_{cyclohexyl}$). ^{13}C NMR (75 MHz, $CDCl_3$) δ 165.7(CO), 138.3, 137.7, 136.7, 135.4, 133.5, 133.3, 133.2, 132.5, 132.3, 132.2, 132.2, 131.2, 128.8, 128.4, 128.3, 127.9, 127.7, 121.2, 112.9, 112.6 (C_{Ar}), 60.0, 55.9, 33.5, 33.2, 25.5, 24.8, 24.4, 13.7 ($C_{Aliphatic}$). MS m/z (EI, 70 eV) 528 (M^+, 13.96), 530 (M^++1, 6.30), 372 (4.75), 210 (7.82), 149 (7.88), 83 (35.85), 55 (100).

11-(Cyclohexylamino)-12-phenyl-9*H*-benzo[*f*]pyrrolo[1,2-*d*][1,2,3]triazolo[1,5-*a*][1,4]diazepine-13-carbonitrile (6a)

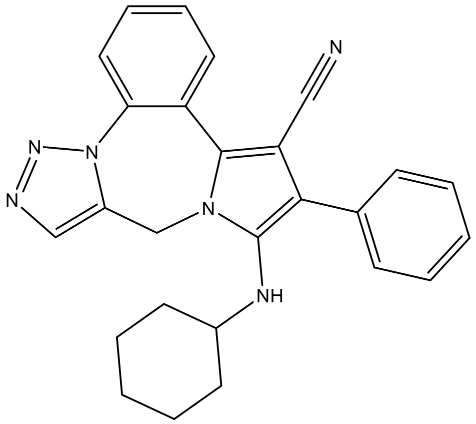

White powder; yield: 166 mg (79%); mp 126-129 °C; R_f (*n*-hexane/EtOAc 2:1) 0.2. Found: C, 74.22; H, 5.73; N, 19.97. $C_{26}H_{24}N_6$ requires C, 74.26; H, 5.75; N, 19.99. (ATR) *ν* (cm^{-1}) 3337 (NH), 29348 (CH), 2857 (CH), 2213 (C≡N), 1447 (C=C) cm^{-1}. ^{1}H NMR (300 MHz, $CDCl_3$) δ 8.22 – 8.06 (m, 2H, H_{Ar}), 7.76 (s, 1H, H_{Ar}), 7.71 – 7.61 (m, 2H, H_{Ar}), 7.50 – 7.42 (m, 3H, H_{Ar}), 7.36 (s, 1H, H_{Ar}), 7.29 (s, 1H, H_{Ar}), 5.92 (bs, 1H, CH_2), 4.63 (bs, 1H, CH_2), 3.49 (s, 1H, NH), 2.56 (bs, 1H, $H_{cyclohexyl}$), 1.76 – 1.58 (m, 4H, $H_{cyclohexyl}$), 1.08 (bs, 4H, $H_{cyclohexyl}$), 0.89 (bs, 2H, $H_{cyclohexyl}$). ^{13}C NMR (75 MHz, $CDCl_3$) δ 137.1, 134.4, 134.1, 132.6, 132.2, 131.4, 130.2, 130.2, 129.7, 128.9, 128.5, 124.4, 121.5, 120.6, 116.6, 115.3 (C_{Ar}), 91.8 (CN), 60.4, 53.8, 35.3, 31.8, 29.3, 21.1, 14.2 ($C_{Aliphatic}$). MS m/z (EI, 70 eV) 420 (M^+, 35.00), 309 (47.76), 295 (12.83), 232 (10.26), 155 (6.97), 128 (6.72), 102 (8.19), 84 (100), 55 (43.28).

12-(4-Bromophenyl)-11-(cyclohexylamino)-9*H*-benzo[*f*]pyrrolo[1,2-*d*][1,2,3]triazolo[1,5-*a*][1,4]diazepine-13-carbonitrile (6b)

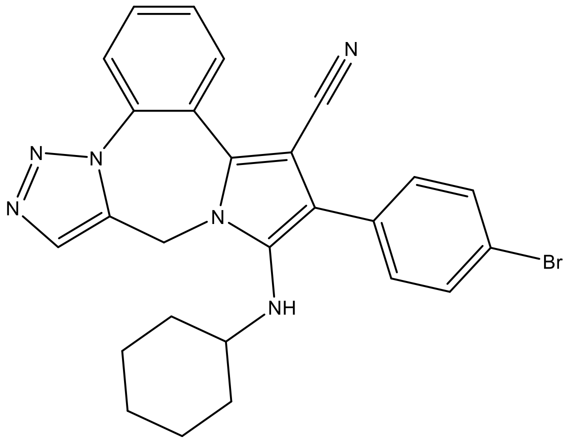

Yellowish white powder; yield: 217 mg (87%); mp 235-239 °C; R_f (*n*-hexane/EtOAc 2:1) 0.10. Found: C, 62.51; H, 4.62; N, 16.81. $C_{26}H_{23}BrN_6$ requires C, 62.53; H, 4.64; N, 16.83. (ATR) *ν* (cm^{-1}) 3346 (NH), 2923 (CH), 2851 (CH), 2217 (C≡N), 1478 (C=C) cm^{-1}. ^{1}H NMR (300 MHz, DMSO-*d*$_6$, $CDCl_3$) δ 8.07 – 7.94 (m, 2H, H_{Ar}), 7.77 (d, *J* = 10.2 Hz, 2H, H_{Ar}), 7.68 – 7.56 (m, 3H, H_{Ar}), 7.50 – 7.45 (m, 2H, H_{Ar}), 7.44 (s, 1H, NH), 5.95 (bs, 1H, CH_2), 4.63 (bs, 1H, CH_2), 2.11 (bs, 1H, $H_{cyclohexyl}$), 1.70 – 1.47 (m, 5H, $H_{cyclohexyl}$), 1.01 (bs, 5H, $H_{cyclohexyl}$). ^{13}C NMR (75 MHz, DMSO-*d*$_6$, $CDCl_3$) δ 135.2, 134.5, 132.7, 131.7, 131.7, 130.2, 129.6, 124.3, 121.5, 121.0, 118.4, 116.8, 111.3, 109.6 (C_{Ar}), 91.1 (CN), 57.7, 50.9, 35.2, 33.8, 29.6, 25.6, 24.8 ($C_{Aliphatic}$). MS m/z (EI, 70 eV) 498 (M^+,

2.33), 500 (M^++1, 2.22), 420 (60.05), 309 (100), 295 (27.54), 282 (21.32), 231 (24.80), 214 (9.30), 155 (6.59), 128 (10.75), 102 (16.04), 83 (18.52), 55 (81.26).

11-(Cyclohexylamino)-12-(naphthalen-2-yl)-9*H*-benzo[*f*]pyrrolo[1,2-*d*][1,2,3]triazolo[1,5-*a*][1,4]diazepine-13-carbonitrile (6c)

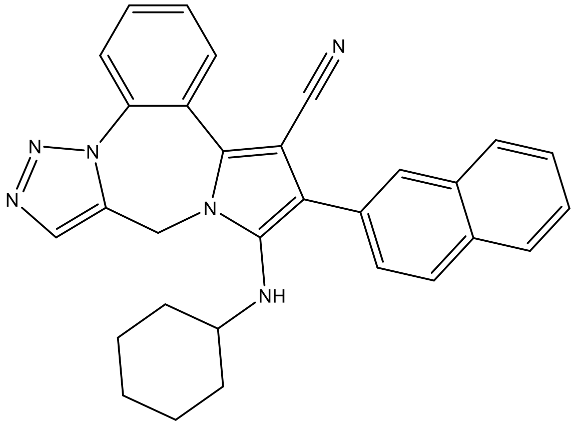

Reddish brown powder; yield: 190 mg (81%); mp 133-137 °C; R_f (*n*-hexane/EtOAc 2:1) 0.10. Found: C, 76.55; H, 5.56; N, 17.82. $C_{30}H_{26}N_6$ requires C, 76.57; H, 5.57; N, 17.86. (ATR) ν (cm^{-1}) 3343 (NH), 2922 (CH), 2852 (CH), 2213 (C≡N), 1450 (C=C) cm^{-1}. ^{1}H NMR (300 MHz, $CDCl_3$) δ 8.22 – 8.09 (m, 2H, H_{Ar}), 7.96 – 7.87 (m, 3H, H_{Ar}), 7.77 (s, 1H, H_{Ar}), 7.70 – 7.61 (m, 2H, H_{Ar}), 7.61 – 7.50 (m, 3H, H_{Ar}), 7.28 (s, 1H, H_{Ar}), 5.95 (bs, 1H, CH_2), 4.64 (bs, 1H, CH_2), 3.39 (bs, 1H, NH), 2.63 – 2.49 (m, 1H, $H_{cyclohexyl}$), 1.63 (bs, 4H, $H_{cyclohexyl}$), 1.26 – 1.18 (m, 2H, $H_{cyclohexyl}$), 1.05 – 0.90 (m, 4H, $H_{cyclohexyl}$). ^{13}C NMR (75 MHz, $CDCl_3$) δ 134.6, 134.1, 133.5, 132.7, 132.5, 131.5, 131.5, 130.3, 129.8, 129.7, 128.7, 128.0, 127.8, 127.4, 126.5, 126.4, 126.2, 124.4, 121.5, 120.7, 116.6 (C_{Ar}), 92.0 (CN), 58.7, 53.7, 33.8, 29.7, 29.3, 25.4, 24.8 ($C_{Aliphatic}$). MS m/z (EI, 70 eV) 470 (M^+, 15.92), 359 (46.50), 345 (11.26), 231 (16.52), 155 (11.64), 97 (13.14), 83 (41.32), 69 (24.64), 55 (100).

11-(*tert*-Butylamino)-12-(4-chlorophenyl)-9*H*-benzo[*f*]pyrrolo[1,2-*d*][1,2,3]triazolo[1,5-*a*][1,4]diazepine-13-carbonitrile (6d)

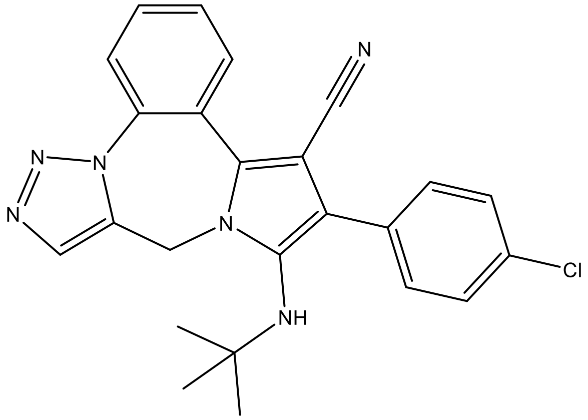

Reddish brown powder; yield: 195 mg (91%); mp 89-92 °C; R_f (*n*-hexane/EtOAc 2:1) 0.09. Found: C, 67.20; H, 4.92; N, 19.57. $C_{24}H_{21}ClN_6$ requires C, 67.21; H, 4.94; N, 19.59. (ATR) ν (cm^{-1}) 3343 (NH), 2969 (CH), 2922 (CH), 2854 (CH), 2216 (C≡N), 1463 (C=C) cm^{-1}. ^{1}H NMR (300 MHz, $CDCl_3$) δ 8.15 (d, J = 2.1 Hz, 2H, H_{Ar}), 7.77 (s, 1H, H_{Ar}), 7.68 (s, 2H, H_{Ar}), 7.50 – 7.29 (m, 4H, H_{Ar}), 6.11 (s, 1H, CH_2), 4.58 (s, 1H, CH_2), 3.23 (s, 1H, NH), 0.96 (s, 9H, $H_{tert\text{-}butyl}$). ^{13}C NMR (75

MHz, $CDCl_3$) δ 133.9, 133.6, 132.8, 132.6, 132.2, 131.7, 130.6, 130.5, 130.3, 129.7, 129.2, 124.2, 123.0, 121.3, 116.4 (C_{Ar}), 92.2 (CN), 55.9, 35.7, 30.1 ($C_{Aliphatic}$). MS m/z (EI, 70 eV) 428 (M^+, 12.11), 430 (M^++1, 5.68), 372 (15.35), 344 (13.76), 329 (13.91), 308 (18.63), 231 (6.16), 155 (6.22), 111 (7.55), 84 (100), 57 (91.31).

11-(*tert*-Butylamino)-12-(4-methoxyphenyl)-9*H*-benzo[*f*]pyrrolo[1,2-*d*][1,2,3]triazolo[1,5-*a*][1,4]diazepine-13-carbonitrile (6e)

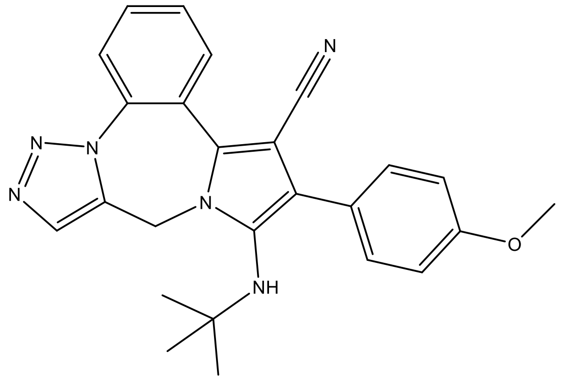

Reddish brown powder; yield: 163 mg (77%); mp 133-137 °C; R_f (*n*-hexane/EtOAc 2:1) 0.09. Found: C, 70.71; H, 5.66; N, 19.75. $C_{25}H_{24}N_6O$ requires C, 70.73; H, 5.70; N, 19.80. (ATR) ν (cm^{-1}) 3343 (NH), 2963 (CH), 2925 (CH), 2854 (CH), 2216 (C≡N), 1474 (C=C) cm^{-1}. ^1H NMR (300 MHz, $CDCl_3$) δ 8.20 – 8.08 (m, 2H, H_{Ar}), 7.76 (s, 1H, H_{Ar}), 7.69 – 7.61 (m, 2H, H_{Ar}), 7.33 (s, 1H, H_{Ar}), 7.28 (s, 1H, H_{Ar}), 6.99 (d, J = 8.4 Hz, 2H, H_{Ar}), 6.10 (d, J = 14.8 Hz, 1H, CH_2), 4.55 (s, 1H, CH_2), 3.85 (s, 3H, CH_3), 3.24 (s, 1H, NH), 0.93 (s, 9H, $H_{tert\text{-}butyl}$). ^{13}C NMR (75 MHz, $CDCl_3$) δ 159.0, 134.1, 132.8, 132.4, 131.9, 130.5, 130.3, 130.1, 129.6, 125.5, 124.2, 124.0, 121.5, 116.7, 114.4 (C_{Ar}), 92.4 (CN), 55.8, 55.3, 53.7, 30.1 ($C_{Aliphatic}$). MS m/z (EI, 70 eV) 424 (M^+, 30.27), 368 (31.47), 339 (30.85), 325 (38.03), 308 (11.08), 295 (16.51), 231 (29.17), 155 (15.99), 102 (14.23), 77 (13.25), 57 (100).

11-(Butylamino)-12-phenyl-9*H*-benzo[*f*]pyrrolo[1,2-*d*][1,2,3]triazolo[1,5-*a*][1,4]diazepine-13-carbonitrile (6f)

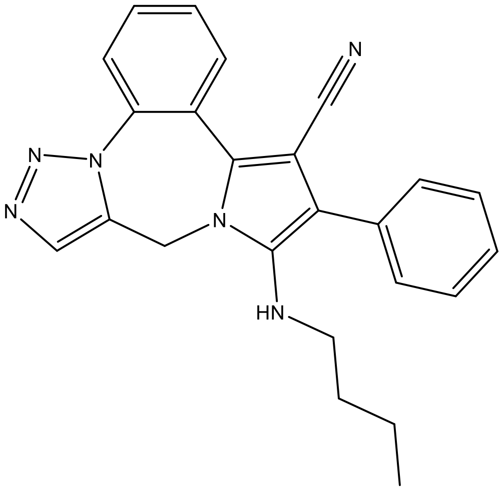

Yellowish white oil; yield: 153 mg (78%); mp 163-165 ˚C; R_f (*n*-hexane/EtOAc 2:1) 0.12. Found: C, 73.05; H, 5.58; N, 21.29. $C_{24}H_{22}N_6$ requires C, 73.07; H, 5.62; N, 21.30. (ATR) *ν* (cm^{-1}) 3352 (NH), 2963 (CH), 2934 (CH), 2857 (CH), 2213 (C≡N), 1483 (C=C) cm^{-1}. ^1H NMR (300 MHz, $CDCl_3$) *δ* 8.22 – 8.04 (m, 2H, H_{Ar}), 7.76 (s, 1H, H_{Ar}), 7.65 (dt, *J* = 6.0, 3.7 Hz, 2H, H_{Ar}), 7.57 – 7.43 (m, 4H, H_{Ar}), 7.40 – 7.33 (m, 1H, H_{Ar}), 7.28 (s, 1H, NH), 5.84 (s, 1H, CH_2), 4.65 (s, 1H, CH_2), 2.87 (t, , *J* = 6 Hz, 2H, $H_{n\text{-butyl}}$), 1.56 – 1.40 (m, 2H, $H_{n\text{-butyl}}$), 1.39 – 1.29 (m, 2H, $H_{n\text{-butyl}}$), 0.87 (t, *J* = 7.3 Hz, 3H, $H_{n\text{-butyl}}$). ^{13}C NMR (75 MHz, $CDCl_3$) *δ* 135.7, 134.1, 132.6, 131.9, 131.4, 130.2, 129.7, 129.0, 128.5, 127.6, 125.2, 124.4, 121.4, 119.6, 116.6 (C_{Ar}), 91.7 (CN), 50.9, 35.26, 32.3, 20.1, 13.8 ($C_{Aliphatic}$). MS m/z (EI, 70 eV) 394 (M^+, 56.99), 323 (27.31), 309 (100), 295 (15.30), 282 (27.45), 255 (12.45), 231 (47.67), 214 (15.66), 167 (16.39), 155 (25.29), 140 (14.96), 128 (26.46), 102 (42.42), 77 (64.40), 57 (85.39).

11-(Butylamino)-12-(o-tolyl)-9*H*-benzo[*f*]pyrrolo[1,2-*d*][1,2,3]triazolo[1,5-*a*][1,4]diazepine-13-carbonitrile (6g)

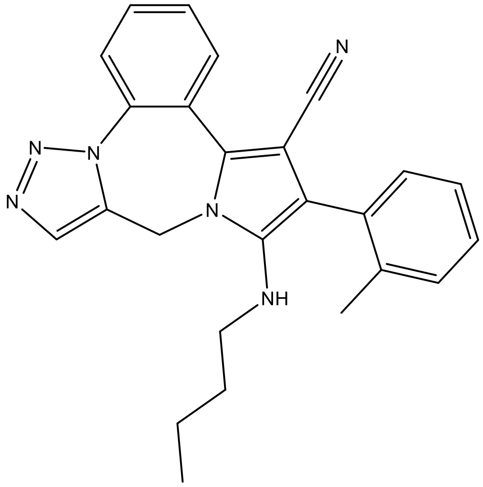

White powder; yield: 146 mg (72%); mp 144-149 ˚C; R_f (*n*-hexane/EtOAc 2:1) 0.12. Found: C, 73.46; H, 5.87; N, 20.55. $C_{25}H_{24}N_6$ requires C, 73.51; H, 5.92; N, 20.57. (ATR) *ν* (cm^{-1}) 3367 (NH), 2963 (CH), 2925 (CH), 2857 (CH), 2219 (C≡N), 1480 (C=C) cm^{-1}. ^1H NMR (300 MHz, $CDCl_3$) *δ* 8.21 – 8.07 (m, 2H, H_{Ar}), 7.78 (s, 1H, H_{Ar}), 7.69 – 7.61 (m, 2H, H_{Ar}), 7.37 – 7.22 (m, 4H, H_{Ar}), 5.84 (bs, 1H, CH_2), 4.67 (bs, 1H, CH_2), 2.95 (s, 1H, NH), 2.74 (bs, 2H, $H_{n\text{-butyl}}$), 1.41 – 1.32 (m, 2H, $H_{n\text{-butyl}}$), 1.27 (s, 3H, CH_3), 1.24 – 1.16 (m, 2H, $H_{n\text{-butyl}}$), 0.82 (t, *J* = 7.4 Hz, 3H, $H_{n\text{-butyl}}$). ^{13}C NMR (75 MHz, $CDCl_3$) *δ* 135.6, 134.2, 132.6, 131.4, 131.4, 131.0, 130.5, 130.1, 129.7, 128.4, 126.0, 124.4, 121.5, 119.4,

116.5 (C_{Ar}), 92.9 (CN), 53.8, 50.5, 35.3, 32.1, 19.9, 13.8 ($C_{Aliphatic}$). MS m/z (EI, 70 eV) 408 (M^+, 80.87), 365 (15.48), 337 (24.26), 323 (100), 308 (35.84), 294 (16.29), 281 (16.40), 231 (62.65), 155 (37.11), 140 (27.27), 129 (25.10), 115 (29.27), 102 (34.37), 77 (27.30), 57 (35.42).

11-(Butylamino)-12-(2-methoxyphenyl)-9*H*-benzo[*f*]pyrrolo[1,2-*d*][1,2,3]triazolo[1,5-*a*][1,4]diazepine-13-carbonitrile (6h)

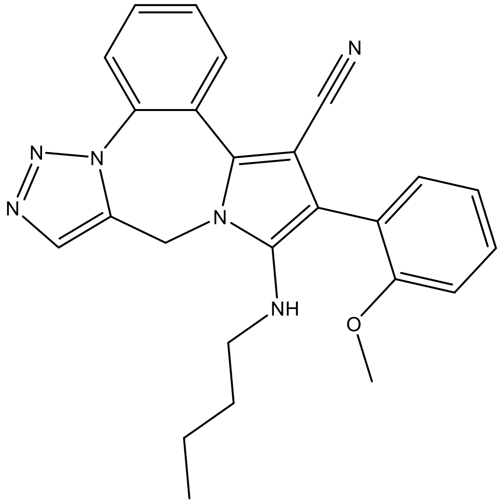

Brown powder; yield: 159 mg (75%); mp 202-206 °C; R_f (*n*-hexane/EtOAc 2:1) 0.09. Found: C, 70.70; H, 5.67; N, 19.76. $C_{25}H_{24}N_6O$ requires C, 70.73; H, 5.70; N, 19.80. (ATR) *v* (cm^{-1}) 3321 (NH), 2965 (CH), 2926 (CH), 2859 (CH), 2212 (C≡N), 1447 (C=C) cm^{-1}. ^1H NMR (300 MHz, $CDCl_3$) δ 8.19 – 8.07 (m, 2H, H_{Ar}), 7.77 (s, 1H, H_{Ar}), 7.66 – 7.62 (m, 1H, H_{Ar}), 7.44 – 7.34 (m, 2H, H_{Ar}), 7.28 (s, 1H, H_{Ar}), 7.14 – 7.01 (m, 2H, H_{Ar}), 5.86 (bs, 1H, CH_2), 4.64 (bs, 1H, CH_2), 3.91 (s, 3H, CH_3), 3.49 (s, 1H, NH), 2.72 (bs, 2H, $H_{n\text{-butyl}}$), 1.26 – 1.14 (m, 4H, $H_{n\text{-butyl}}$), 0.76 (t, J = 7.1 Hz, 3H, $H_{n\text{-butyl}}$). ^{13}C NMR (75 MHz, $CDCl_3$) δ 155.9, 136.8, 134.2, 132.5, 131.4, 129.7, 129.2, 127.6, 127.0, 124.4, 121.5, 121.5, 120.7, 120.2, 116.7, 115.7, 111.4 (C_{Ar}), 92.2 (CN), 55.9, 50.4, 35.2, 32.2, 20.0, 13.7 ($C_{Aliphatic}$). MS m/z (EI, 70 eV) 424 (M^+, 14.36), 372 (11.48), 329 (10.05), 307 (13.83), 231 (10.88), 155 (13.40), 135 (21.48), 101 (27.70), 84 (51.92), 57 (100).

X-ray crystallographic information

Preparation method of a single crystal of compound 4h

To obtain single crystals of **4h**, 50 mg of the compound were dissolved in 0.2 mL of hot ethyl acetate in a 10 mL vial. Subsequently, 0.8 mL of *n*-hexane were added to the vial. The vial was sealed with a lid, and single crystal particles were first observed after three days. Five days later, the single crystal particles had substantially grown and were ready to determine the structure using crystallography. ORTEP diagram for **4h**; summary of data: The Cambridge Crystallographic Data Centre (CCDC) 2365305; unit cell parameters: a = 12.830 (3) A alpha = 90 deg, b = 12.634 (3) A beta = 98.10 (3) deg, c = 14.283 (3) A gamma = 90 deg.

	Calculated	Reported
Volume	2292.1 (9)	2292.1 (9)
Space group	p 21/n	p 21/n
Hall group	-p 2yn	-p 2yn
Moiety formula	$C_{28} H_{25} N_3O$	$C_{28} H_{25} N_3O$
Sum formula	$C_{28} H_{25} N_3O$ [+ solvent]	$C_{28} H_{25} N_3O$ [+ solvent]
Mr	419.51	419.51
Dx,g cm^{-3}	1.216	1.216
Z	4	4
Mu (mm-1)	0.075	0.075
F000	888.0	888.0
F000’	888.32	
h,k,lmax	15,15,16	15,15,16
Nref	4027	4024
Tmin,Tmax	0.982, 0.989	
Tmin’	0.963	
Correction method = Not given, Data completeness = 0.999, Theta(max) = 25.000, R(reflections) = 0.0723 (2024), wR2(reflections) = 0.1490 (2024), S = 0.943, Npar = 297		

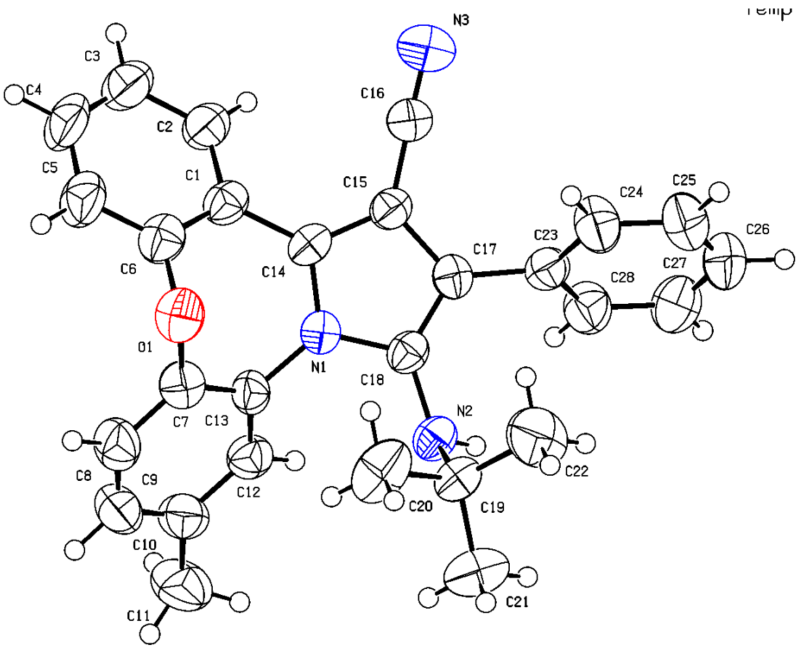

Figure S1. X-ray crystal structure of compound **4h**.

Preparation method of a single crystal of compound 6a

To obtain single crystals of compound **6a**, 60 mg of the compound were dissolved in 0.4 mL of hot chloroform solvent in a 10 mL vial. Subsequently, 1 mL of *n*-hexane was added to the vial. The vial was sealed with a lid, and single crystal particles were first observed after ten days. Fifteen days later, the single crystal particles had substantially grown and were ready to determine the structure using crystallography.

ORTEP diagram for **6a**; summary of data: The Cambridge Crystallographic Data Centre (CCDC) 2365306; unit cell parameters: a = 32.586(6) A alpha = 90 deg, b = 11.376(2) A beta = 123.10(3) deg, c = 18.366(4) A gamma = 90 deg.

	Calculated	Reported
Volume	5703 (3)	5703 (3)
Space group	C 2/c	C 2/c
Hall group	-C 2yc	-C 2yc
Moiety formula	C_{26} H_{24} N_6	C_{26} H_{24} N_6
Sum formula	C_{27} H_{25} $C_{13}N_6$ [+ solvent]	C_{27} H_{25} $C_{13}N_6$ [+ solvent]
Mr	539.88	539.88
Dx,g cm^{-3}	1.258	1.257
Z	8	8
Mu (mm-1)	0.348	0.348
F000	2240.00	2240.00
F000’	2244.14	
h,k,lmax	38,13,21	38,13,21
Nref	5021	5013
Tmin,Tmax	0.882, 0.933	
Tmin’	0.840	
Correction method = Not given, Data completeness = 0.998, Theta(max) = 25.000, R(reflections) = 0.0876 (2257), wR2(reflections) = 0.2617 (5013), S = 0.887, Npar = 298		

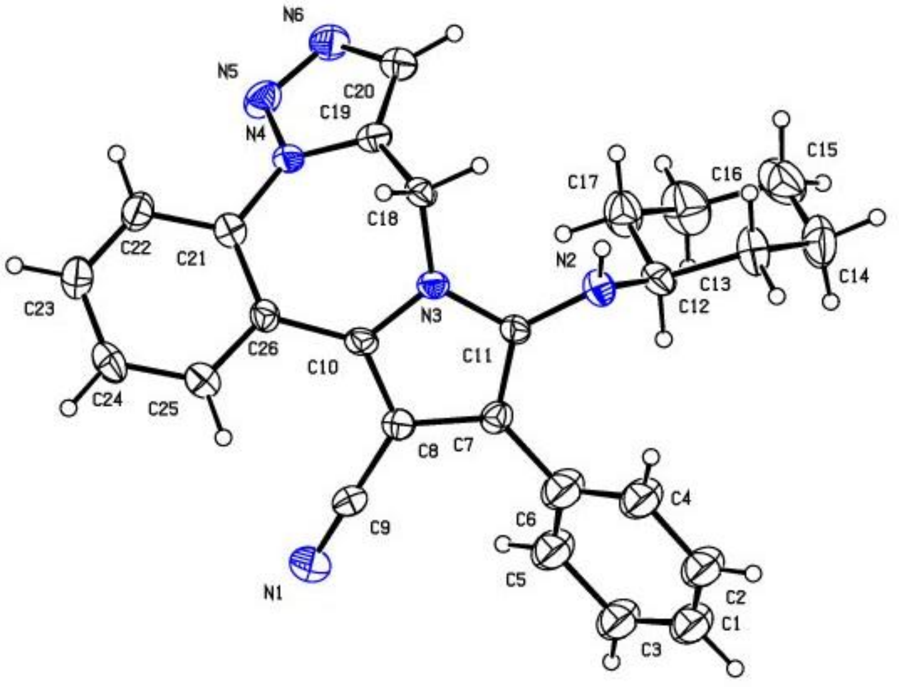

Figure S2. X-ray crystal structure of compound **6a**.

Photophysical study

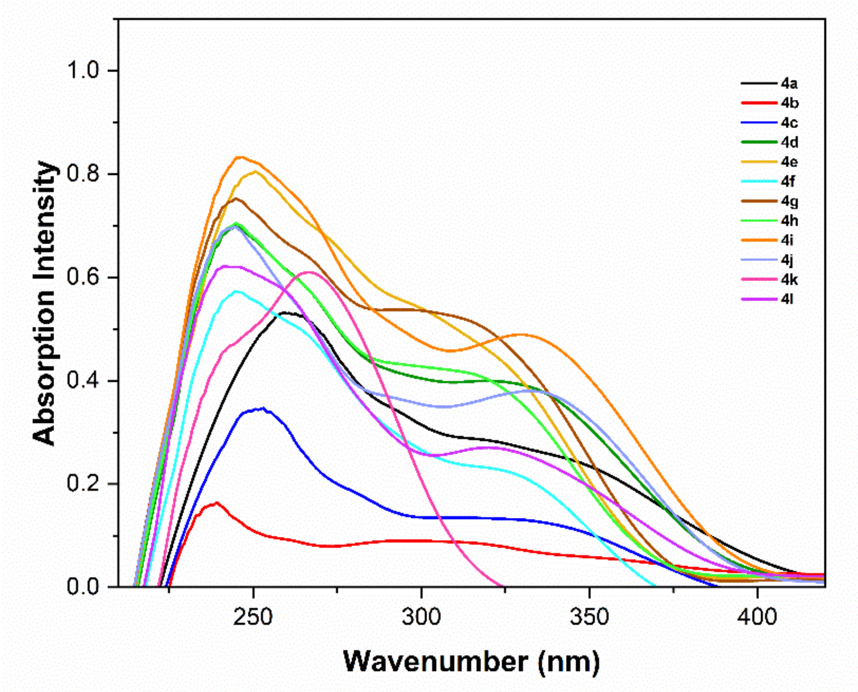

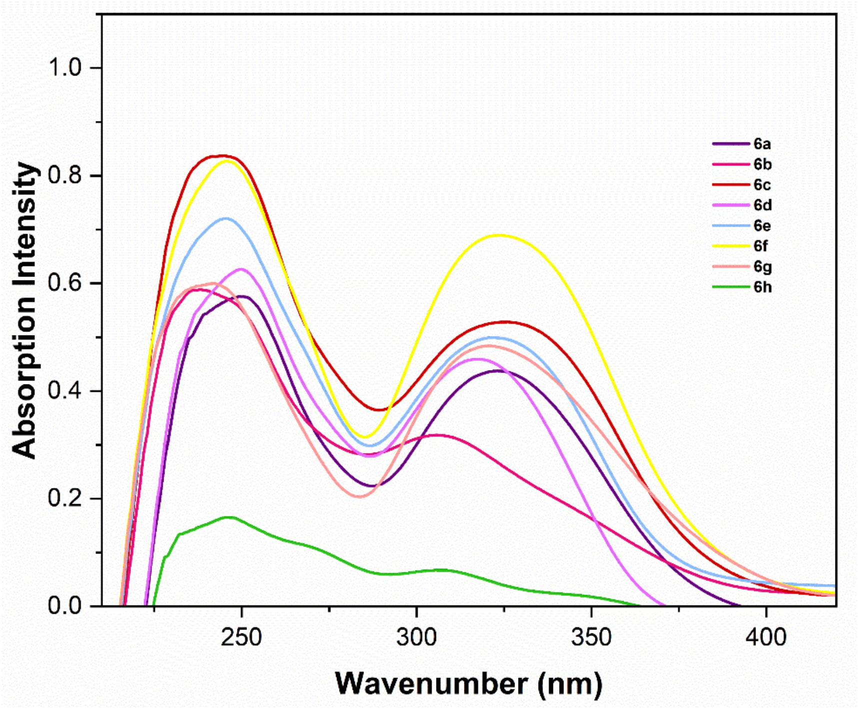

Figure S3. UV–vis absorption pyrrole-fused derivatives **4a–l** and **6a–h**; c = 75 ppm in EtOH and T = 298 K

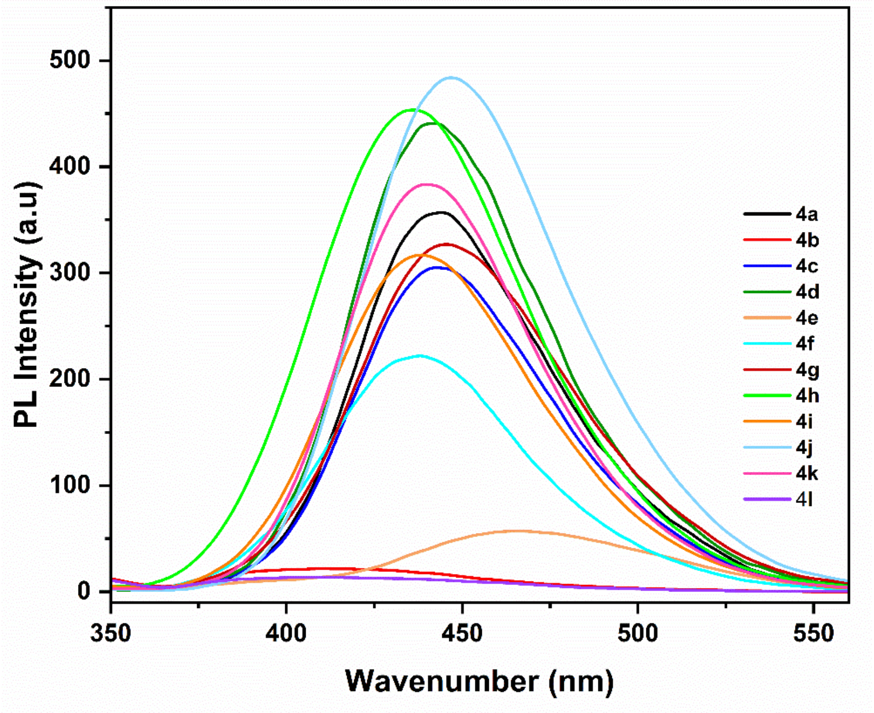

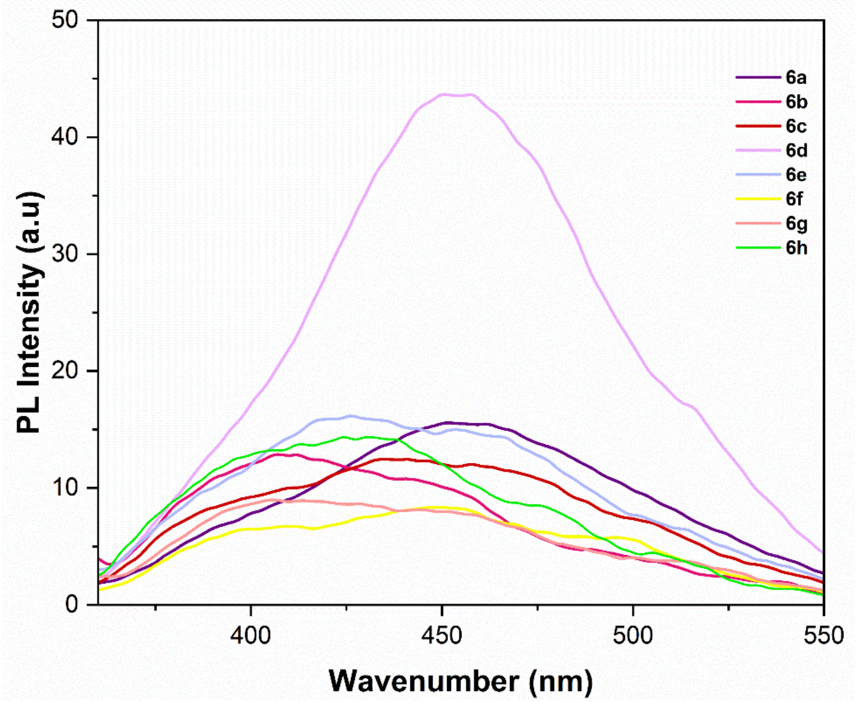

Figure S4. Emission pyrrole-fused derivatives **4a–l** and **6a–h**; c = 75 ppm in EtOH and T = 298 K;

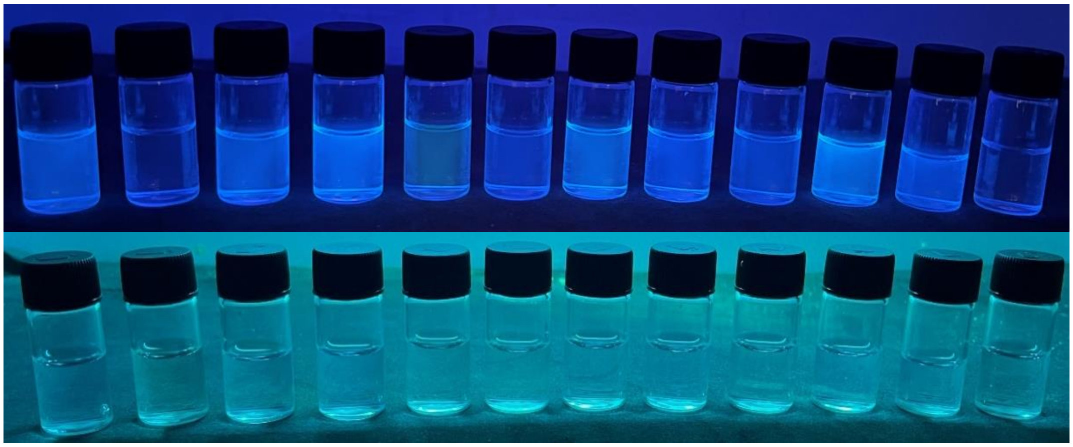

Figure S5. The digital photograph under sunlight and UV light 265 nm for pyrrole-fused dibenzoxazepine/thoazepine derivatives **4a–l**.

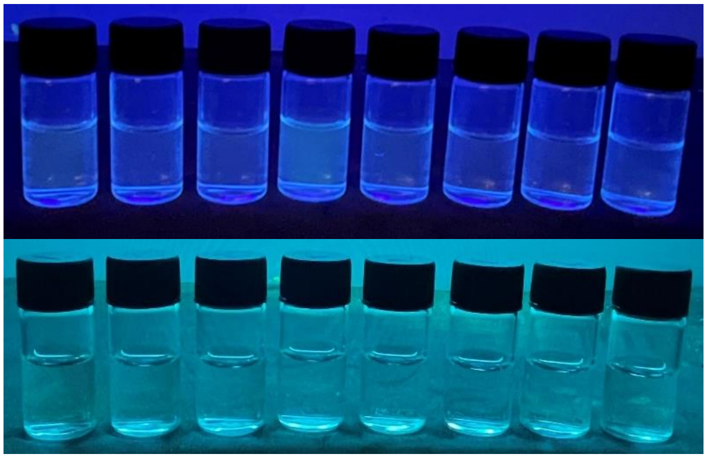

Figure S6. The digital photograph under sunlight and UV light 265 nm for pyrrole-fused triazolobenzodiazepine derivatives **6a–h**.

Fluorescence quantum yield in solution

Fluorescence quantum yield was determined using the optical method for products **4a–l** and **6a–h** in ethanol solution with a concentration of 75 ppm. Quinine sulfate solutions (Φ_f = 0.546 in 1 N H_2SO_4) were used as standards at an excitation wavelength of 320–345 nm. The quantum yield is calculated using equation 1.

$$\Phi_f = \Phi_r (A_r F_s / A_s F_r)(\eta s^2 / \eta r^2).........(1)$$

Where, A_s and A_r are the absorbances of the sample and reference solutions, respectively, at the same excitation wavelength, F_s and F_r are the corresponding relative integrated fluorescence intensities and η is the refractive index of the solvents.[1] The quantum yields of products **4a–l** and **6a–h** were calculated according to equation 1 and are collected in Table S1.

Table S1. Quantum yields of products **4a–l** and **6a–h**

Substrate	Absorption λ_{max} (nm)	Emission λ_{max} (nm)	Φ_f[a] (%)
**4a**	320	446	48.35
**4b**	317	413	14.10
**4c**	314	447	42.10
**4d**	315	442	34.58
**4e**	305	434	20.54
**4f**	325	445	42.14
**4g**	311	434	39.39
**4h**	315	442	44.03
**4i**	331	446	29.48
**4j**	340	443	47.79
**4k**	266	403	3.32
**4l**	322	465	8.74
**6a**	325	457	0.90
**6b**	306	408	0.83
**6c**	319	461	1.04
**6d**	317	454	0.74
**6e**	322	424	0.98
**6f**	324	449	0.64
**6g**	320	407	0.85
**6h**	308	428	0.91

[a]Fluorescence quantum yield relative to quinine sulfate as a standard

^{1}H NMR, ^{13}C NMR and mass spectra of the compounds

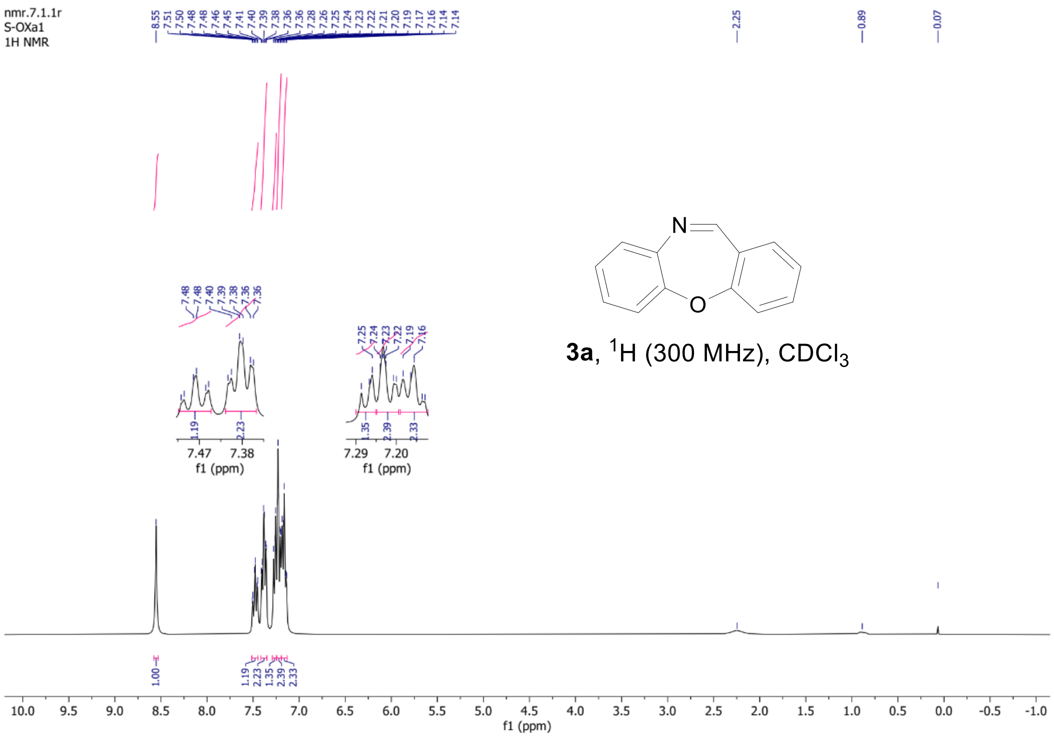

3a, ^{1}H (300 MHz), $CDCl_3$

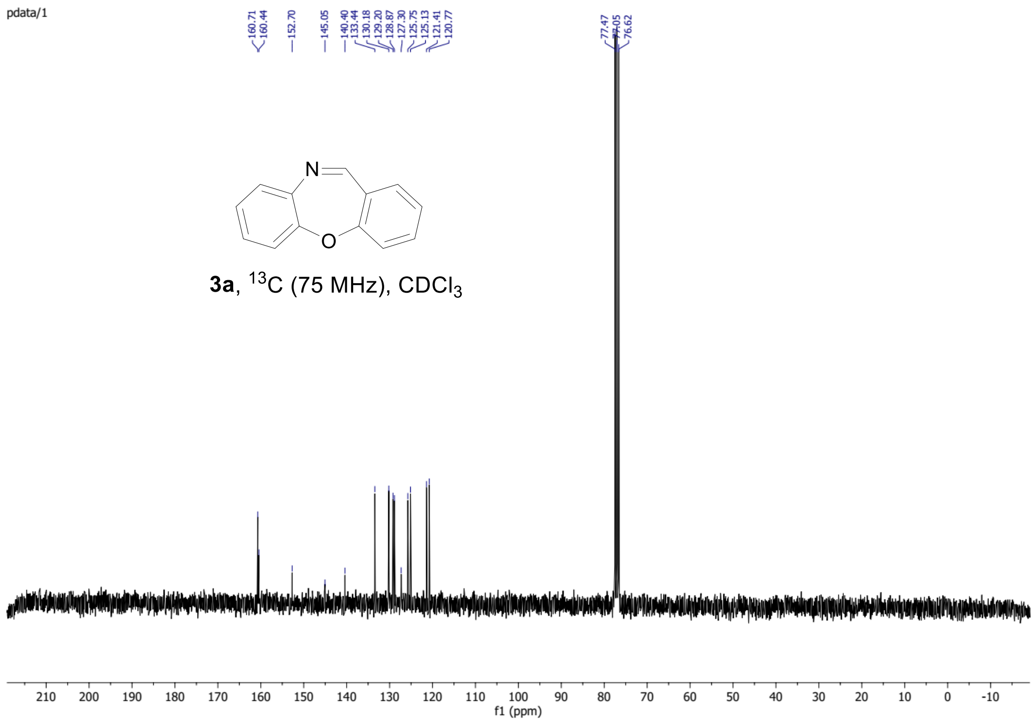

3a, ^{13}C (75 MHz), $CDCl_3$

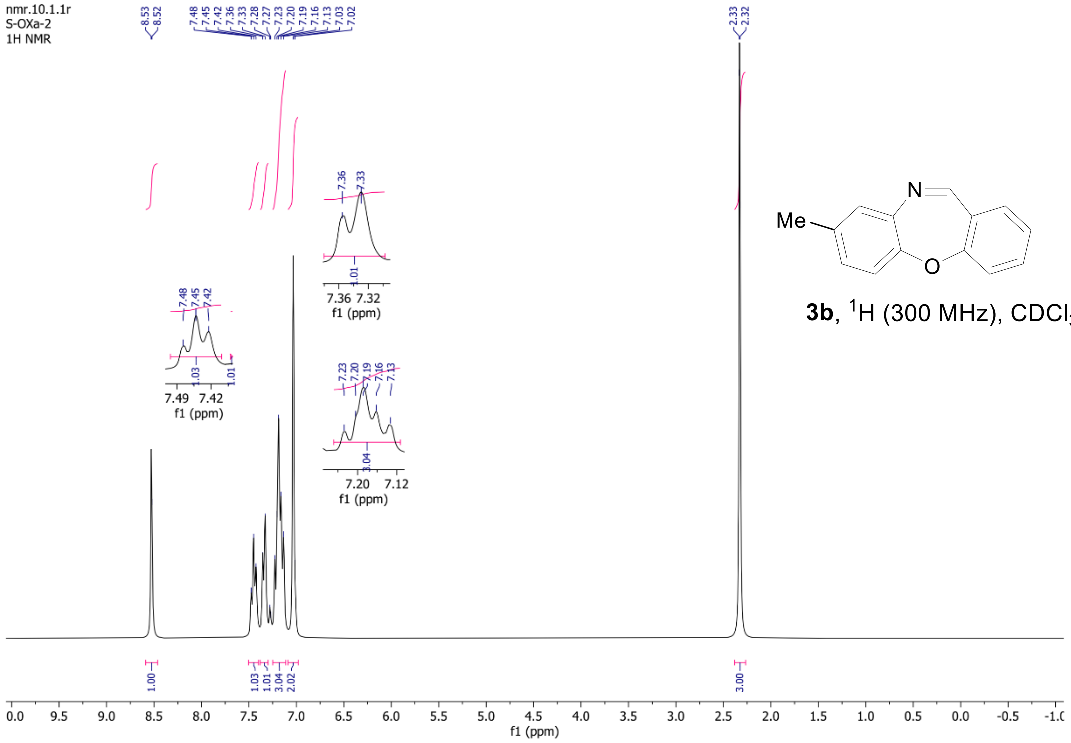

3b, ^{1}H (300 MHz), $CDCl_3$

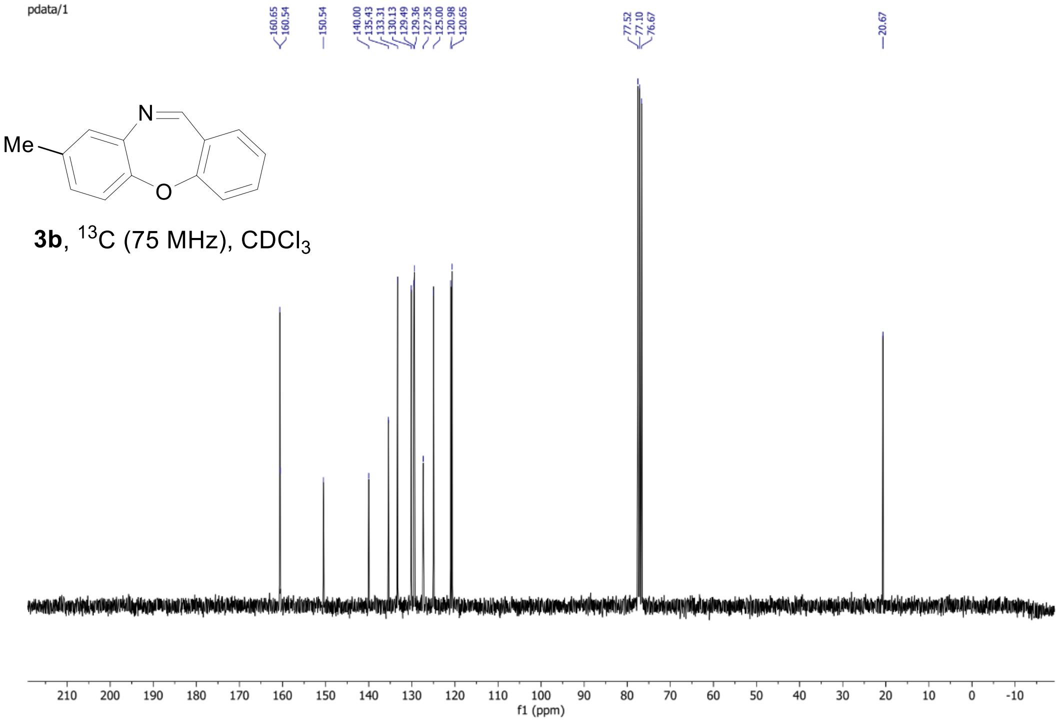

3b, ^{13}C (75 MHz), $CDCl_3$

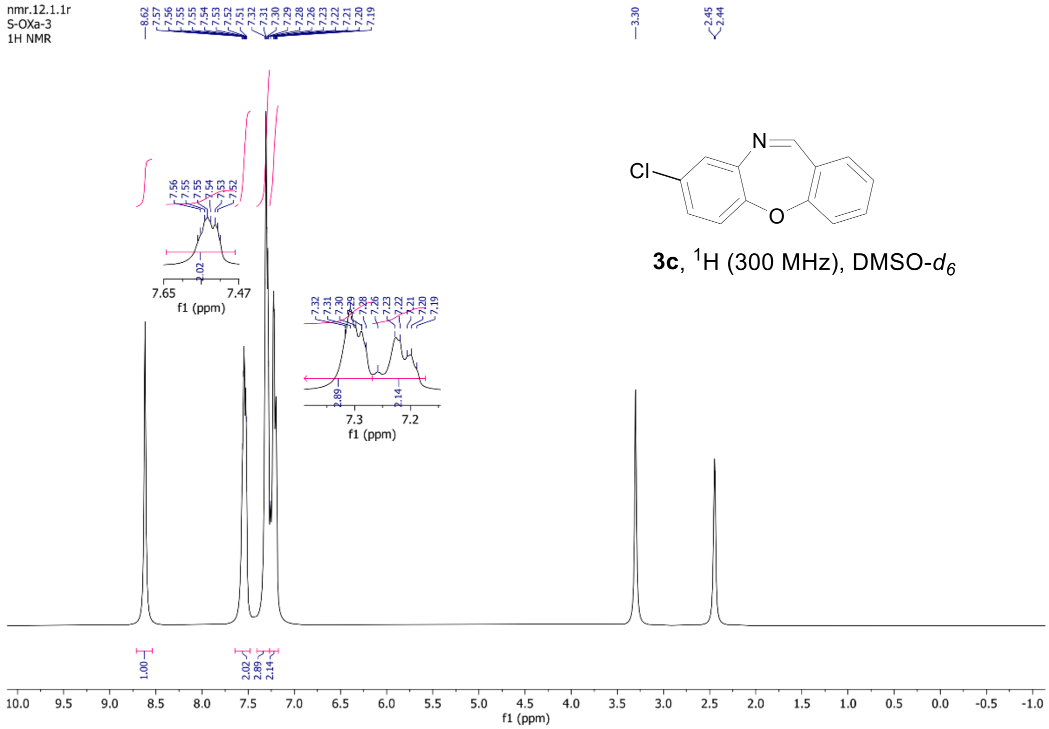
nmr.12.1.1r
S-OXa-3
1H NMR
3c, 1H (300 MHz), DMSO-d6
f1 (ppm)

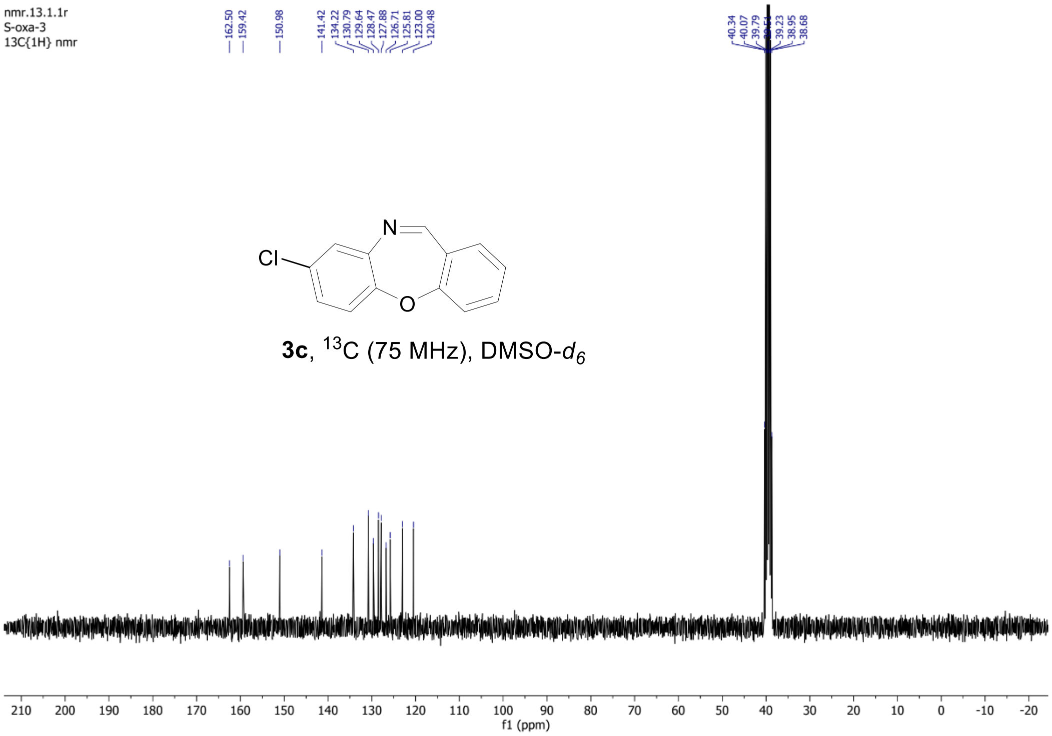
nmr.13.1.1r
S-oxa-3
13C{1H} nmr
3c, 13C (75 MHz), DMSO-d6
f1 (ppm)

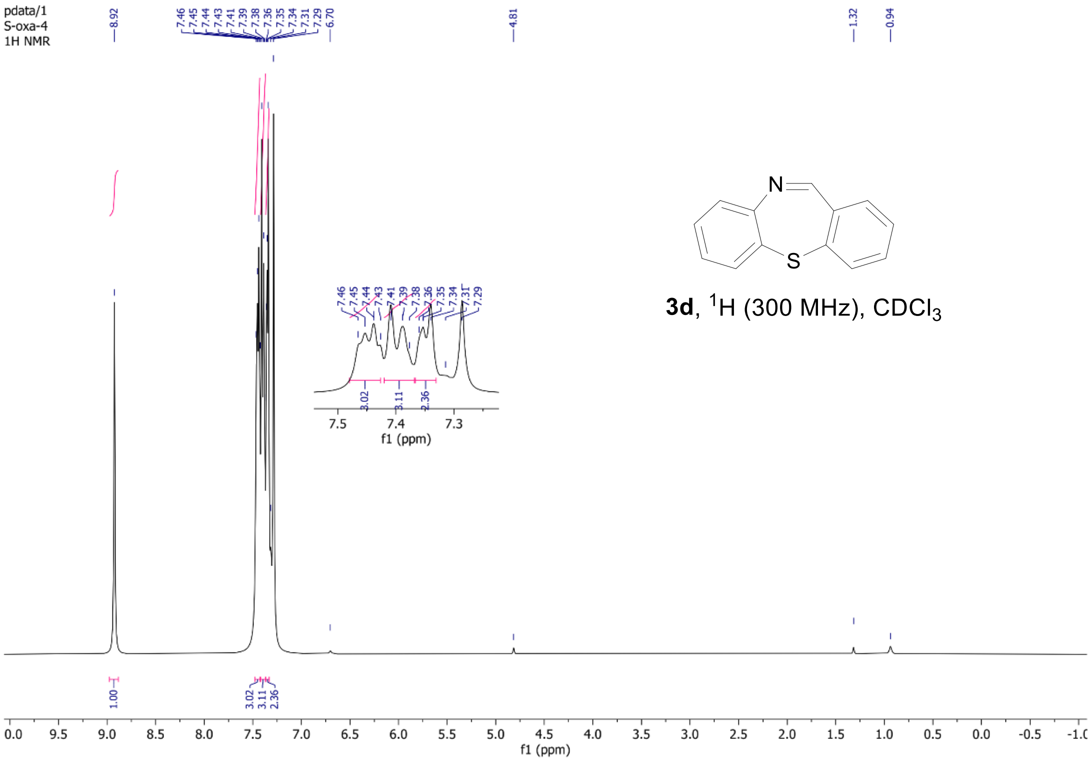
pdata/1
S-oxa-4
1H NMR
3d, ¹H (300 MHz), CDCl₃
f1 (ppm)

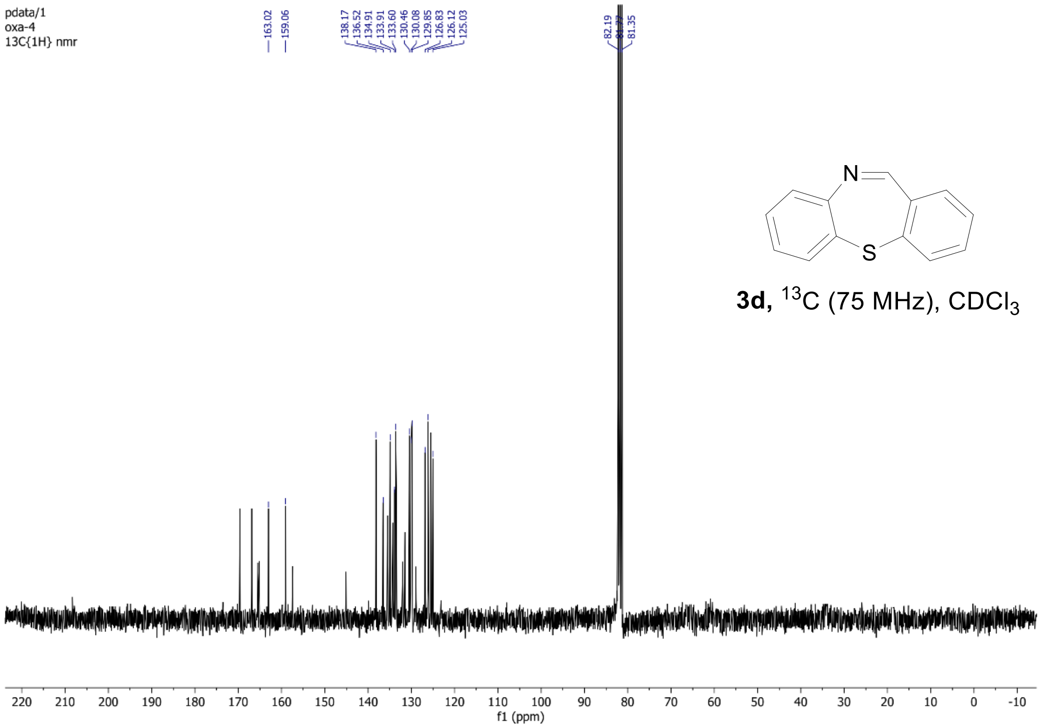
pdata/1
oxa-4
13C{1H} nmr
3d, ¹³C (75 MHz), CDCl₃
f1 (ppm)

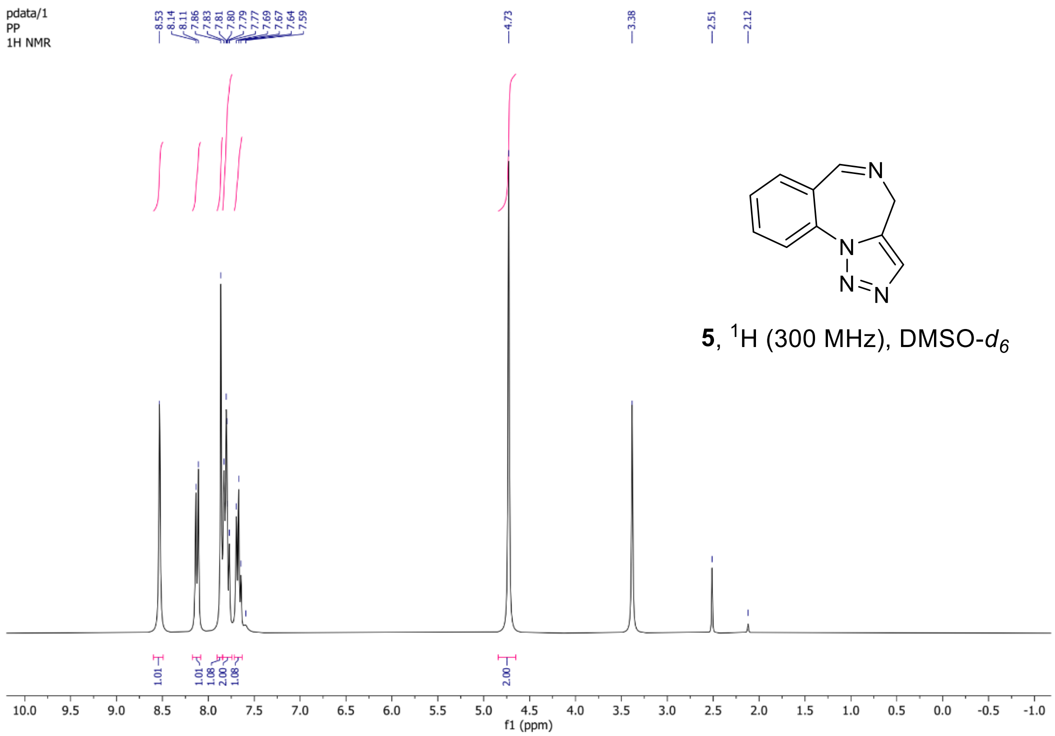
pdata/1
PP
1H NMR
8.53
8.14
8.11
7.86
7.83
7.81
7.80
7.79
7.77
7.69
7.67
7.64
7.59
4.73
3.38
2.51
2.12
1.01
1.01
1.08
2.00
1.08
2.00
N
N
N
N
5, ¹H (300 MHz), DMSO-d6
f1 (ppm)

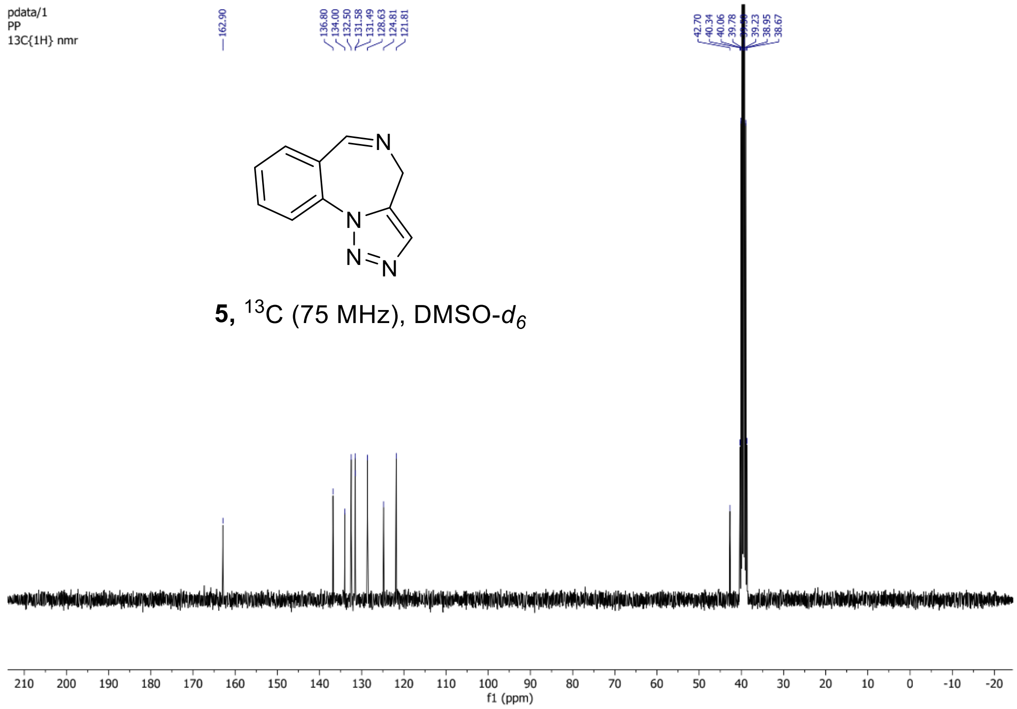
pdata/1
PP
13C{1H} nmr
162.90
136.80
134.00
132.50
131.58
131.49
128.63
124.81
121.81
42.70
40.34
40.06
39.78
39.23
38.95
38.67
N
N
N
N
5, ¹³C (75 MHz), DMSO-d6
f1 (ppm)

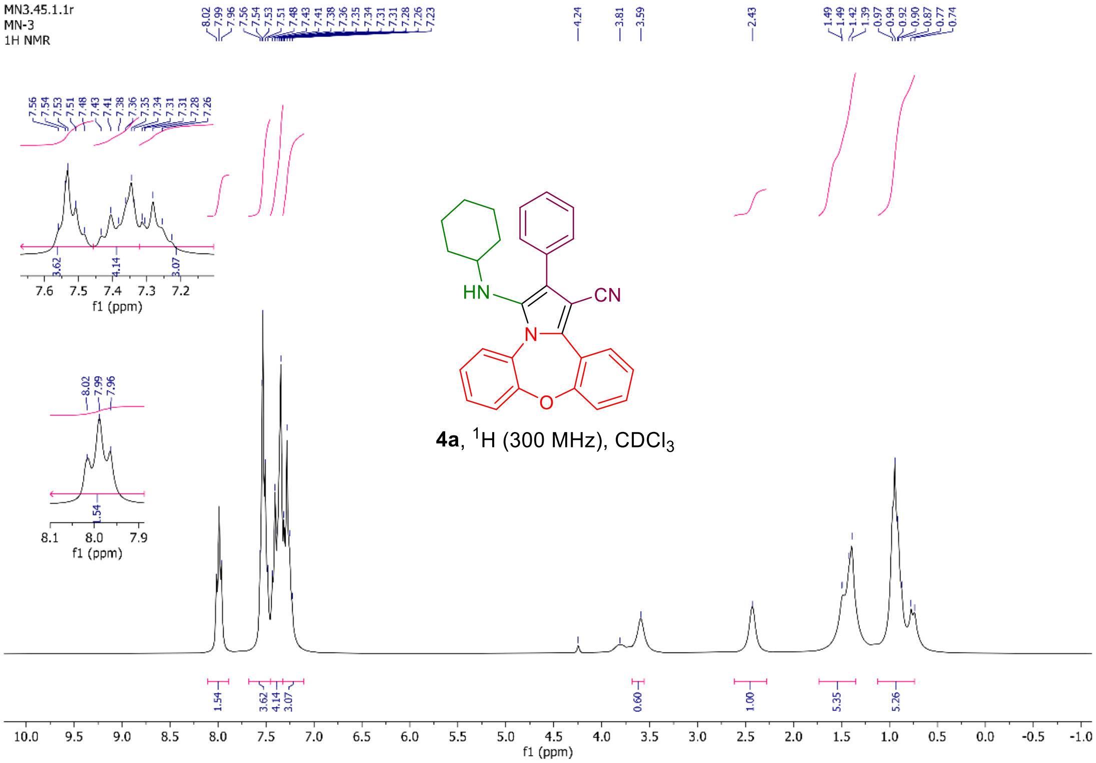

4a, ^{1}H (300 MHz), $CDCl_3$

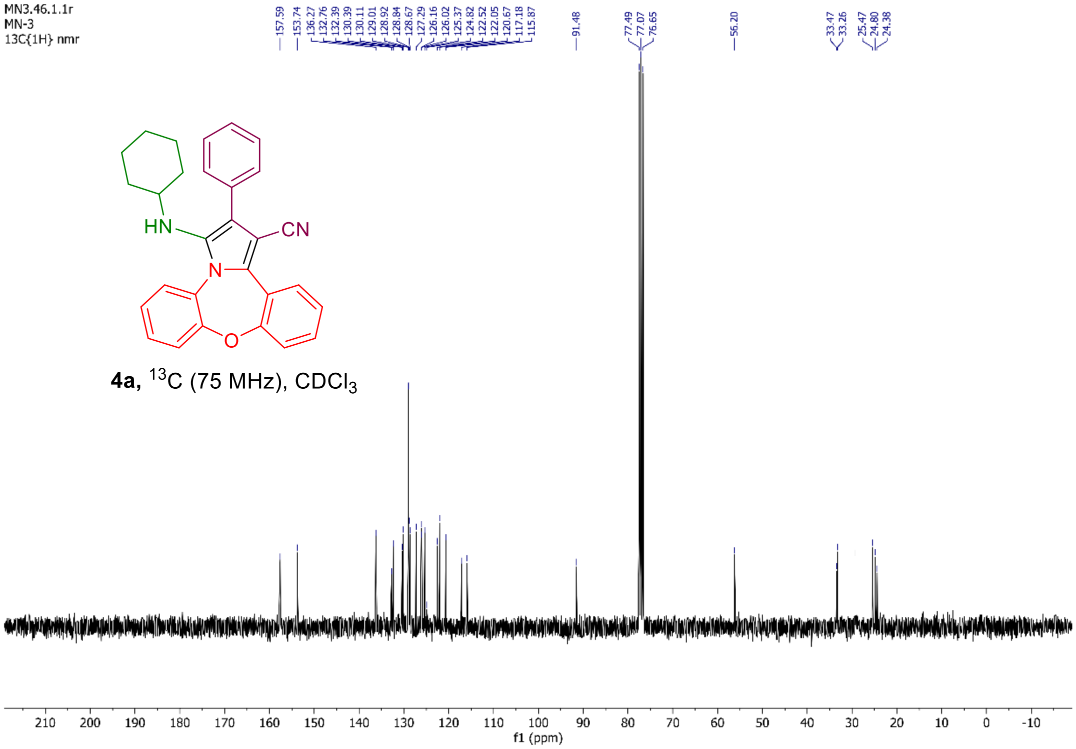

4a, ^{13}C (75 MHz), $CDCl_3$

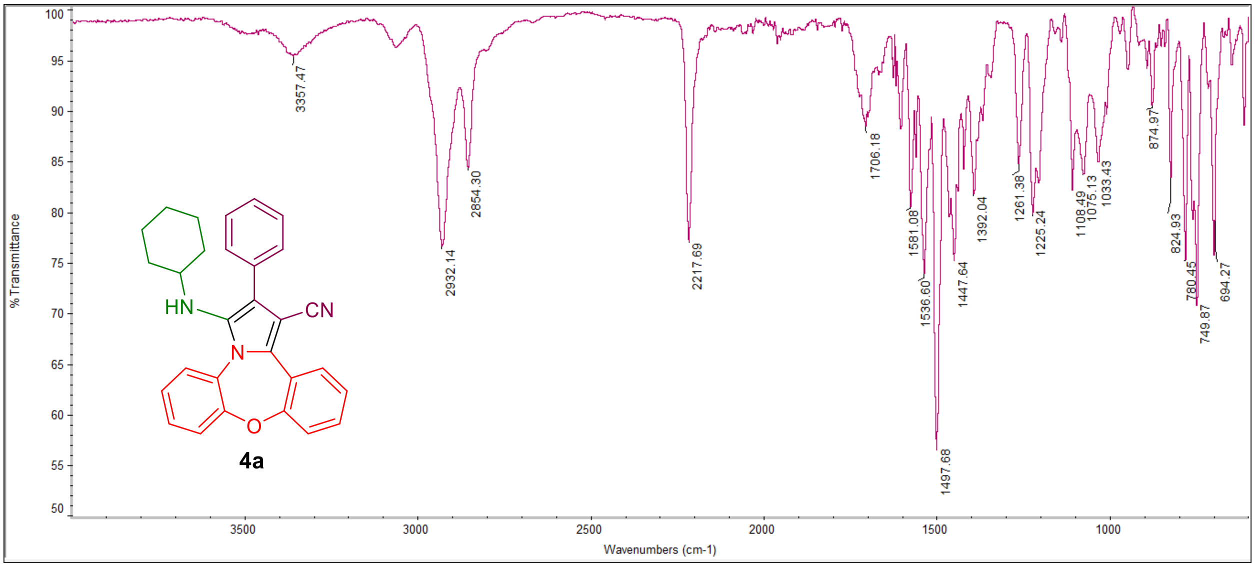
% Transmittance
Wavenumbers (cm-1)
3357.47
2932.14
2854.30
2217.69
1706.18
1581.08
1536.60
1497.68
1447.64
1392.04
1261.38
1225.24
1108.49
1075.13
1033.43
874.97
824.93
780.45
749.87
694.27
HN
CN
N
O
4a

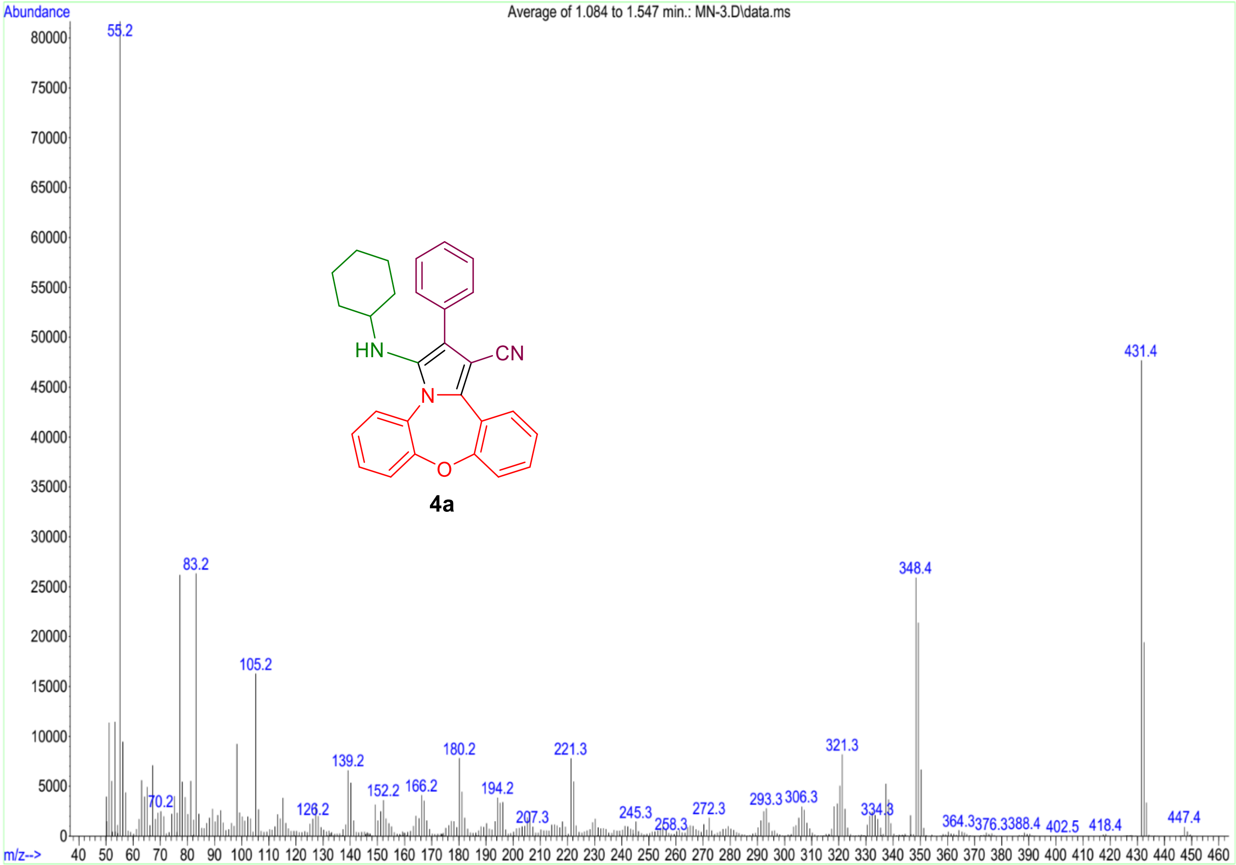
Abundance
Average of 1.084 to 1.547 min.: MN-3.D\data.ms
m/z-->
55.2
83.2
105.2
139.2
180.2
221.3
321.3
348.4
431.4
447.4
HN
CN
N
O
4a

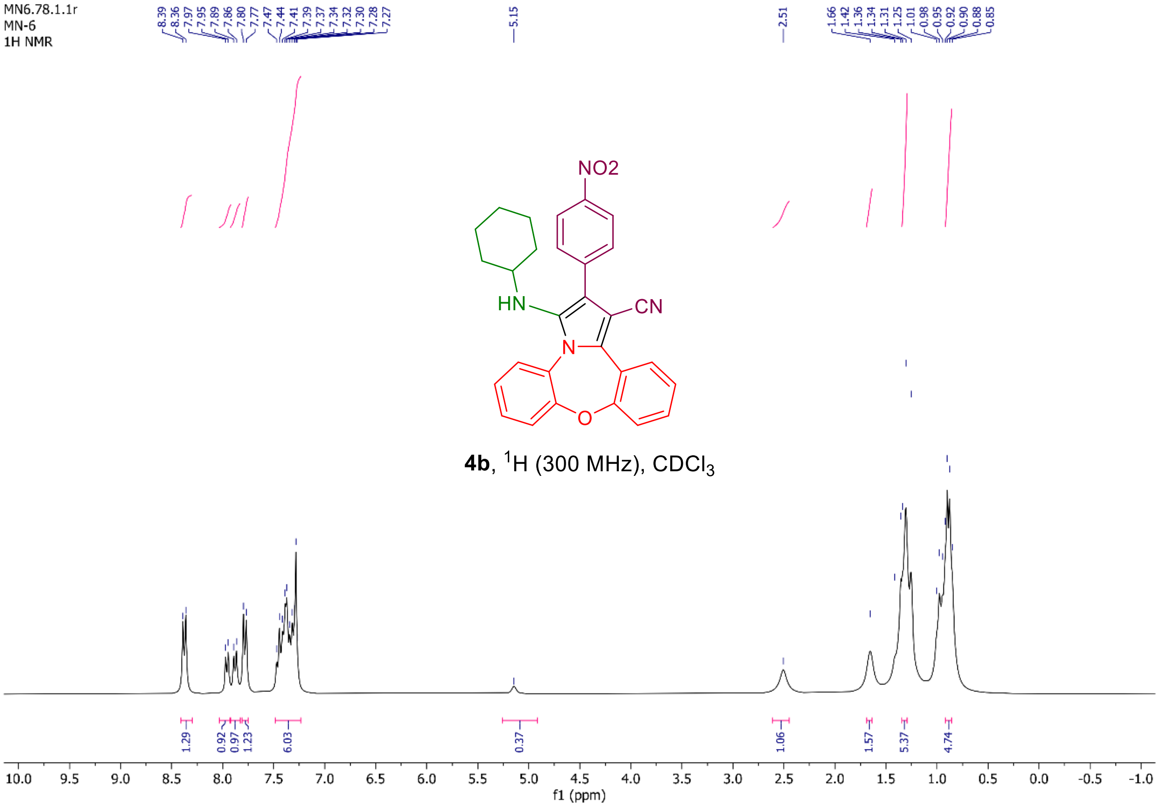

4b, ^{1}H (300 MHz), $CDCl_3$

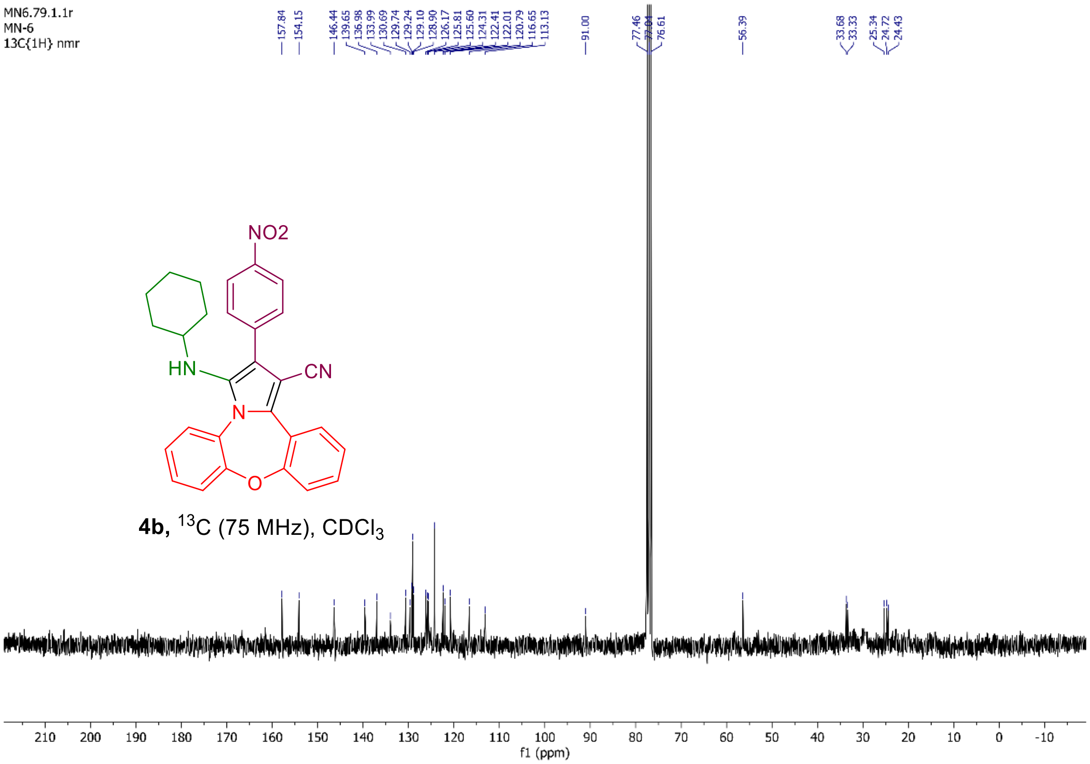

4b, ^{13}C (75 MHz), $CDCl_3$

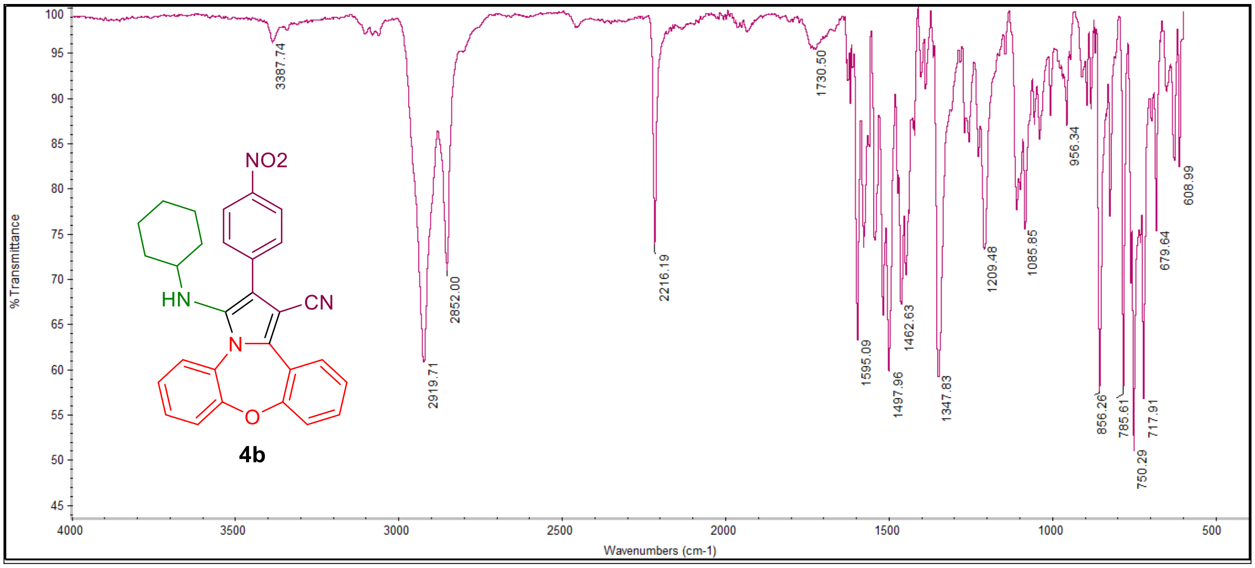

% Transmittance
Wavenumbers (cm-1)
3387.74
2919.71
2852.00
2216.19
1730.50
1595.09
1497.96
1462.63
1347.83
1209.48
1085.85
956.34
856.26
785.61
750.29
717.91
679.64
608.99
NO2
HN
CN
N
O
4b

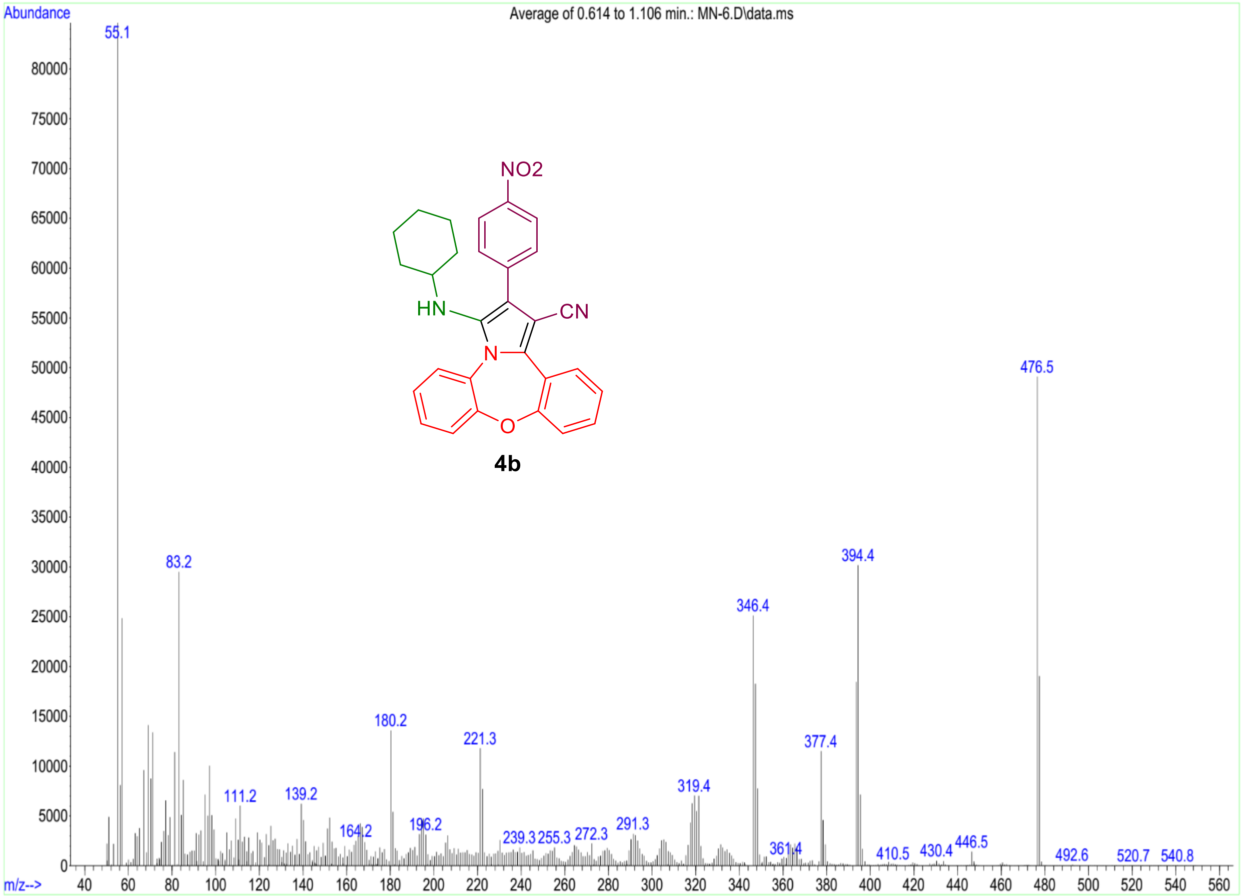

Abundance
Average of 0.614 to 1.106 min.: MN-6.D\data.ms
m/z-->
55.1
83.2
111.2
139.2
164.2
180.2
196.2
221.3
239.3
255.3
272.3
291.3
319.4
346.4
361.4
377.4
394.4
410.5
430.4
446.5
476.5
492.6
520.7
540.8
NO2
HN
CN
N
O
4b

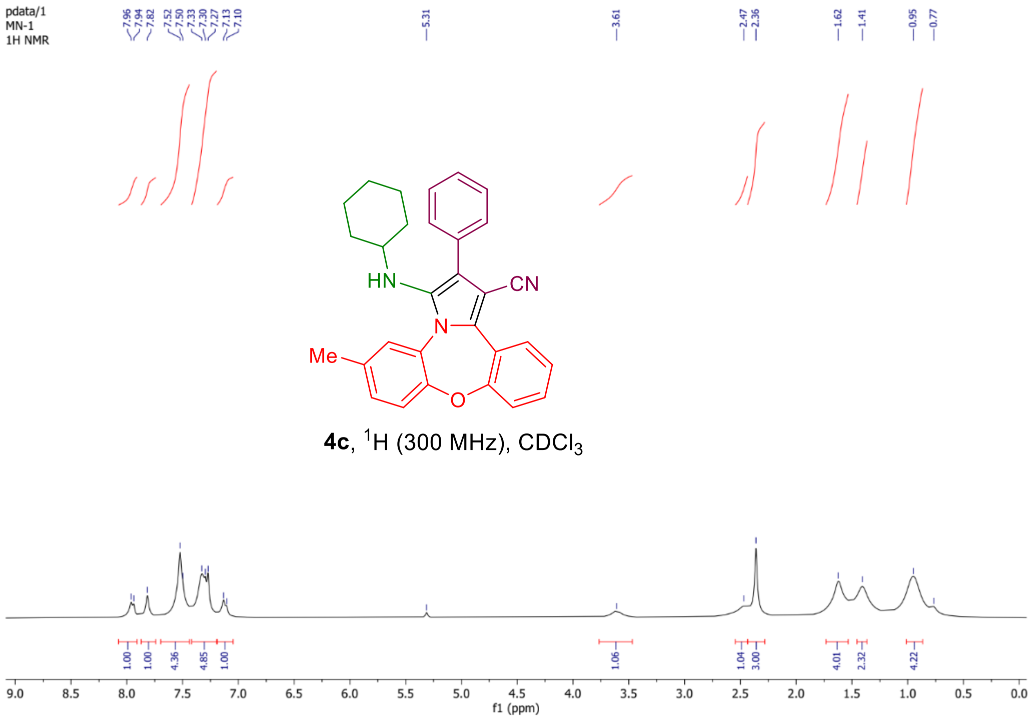

4c, ^{1}H (300 MHz), $CDCl_3$

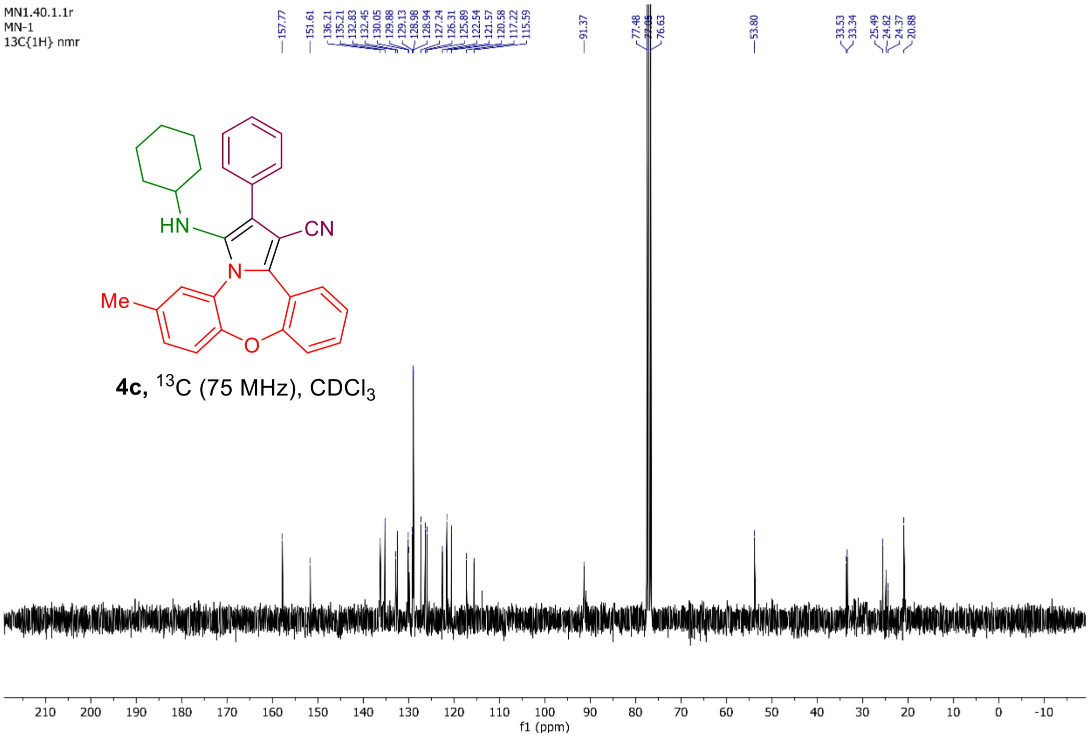

4c, ^{13}C (75 MHz), $CDCl_3$

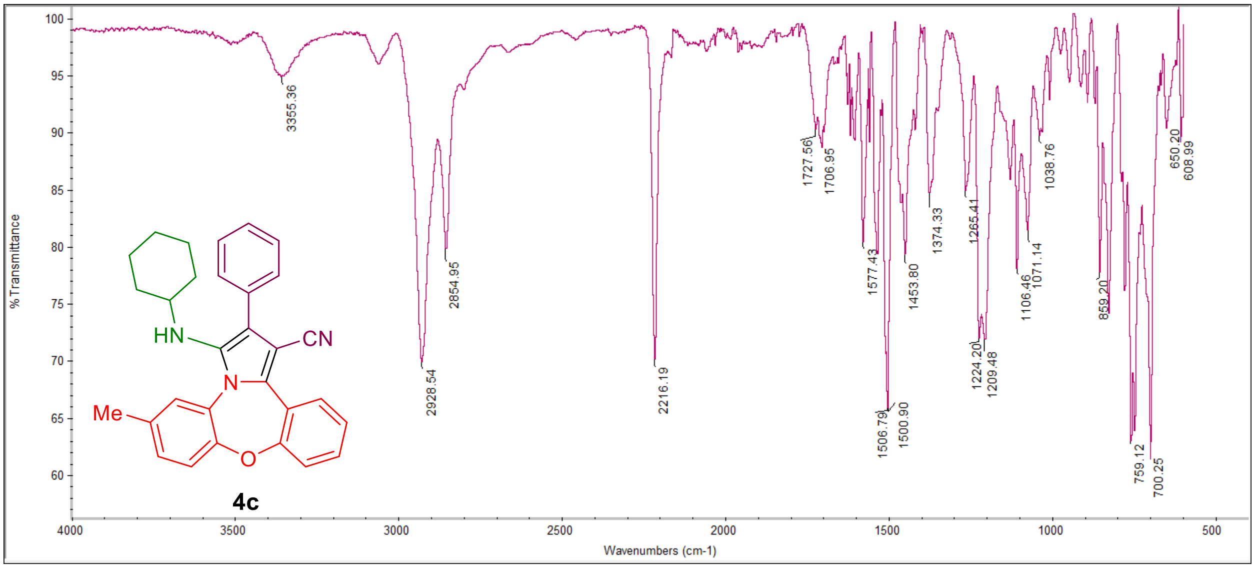

% Transmittance
Wavenumbers (cm-1)
3355.36
2928.54
2854.95
2216.19
1727.56
1706.95
1577.43
1506.79
1500.90
1453.80
1374.33
1265.41
1224.20
1209.48
1106.46
1071.14
1038.76
859.20
759.12
700.25
650.20
608.99
HN
CN
N
Me
O
4c

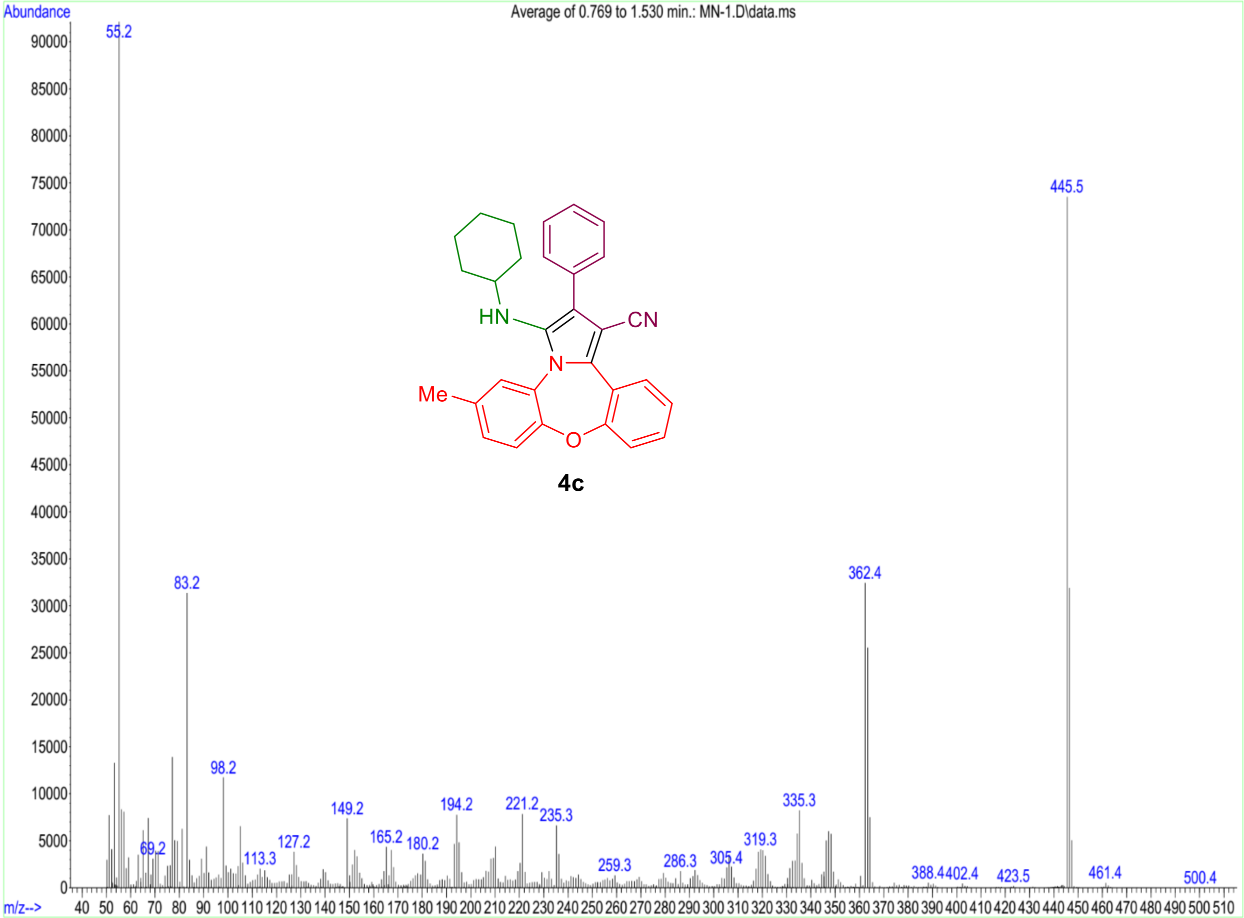

Abundance
Average of 0.769 to 1.530 min.: MN-1.D\data.ms
m/z-->
55.2
83.2
69.2
98.2
113.3
127.2
149.2
165.2
180.2
194.2
221.2
235.3
259.3
286.3
305.4
319.3
335.3
362.4
388.4
402.4
423.5
445.5
461.4
500.4
HN
CN
N
Me
O
4c

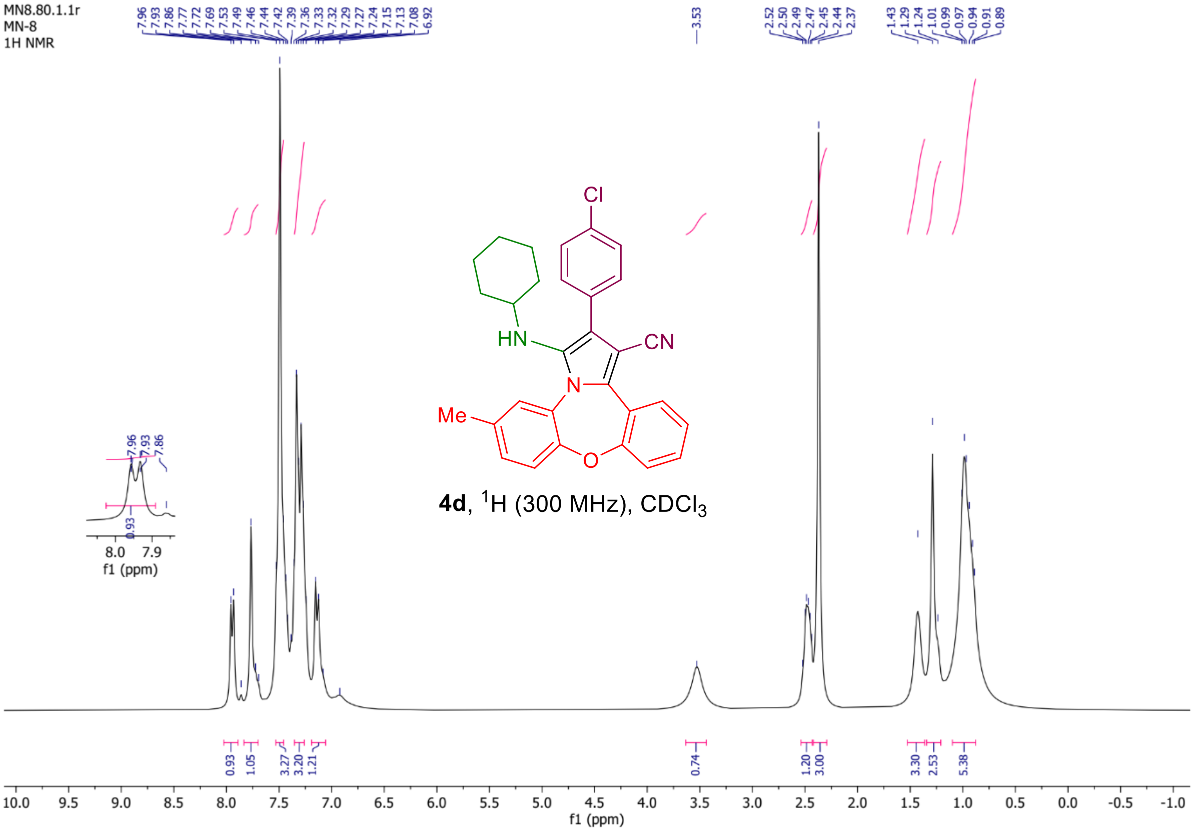

4d, ^{1}H (300 MHz), $CDCl_3$

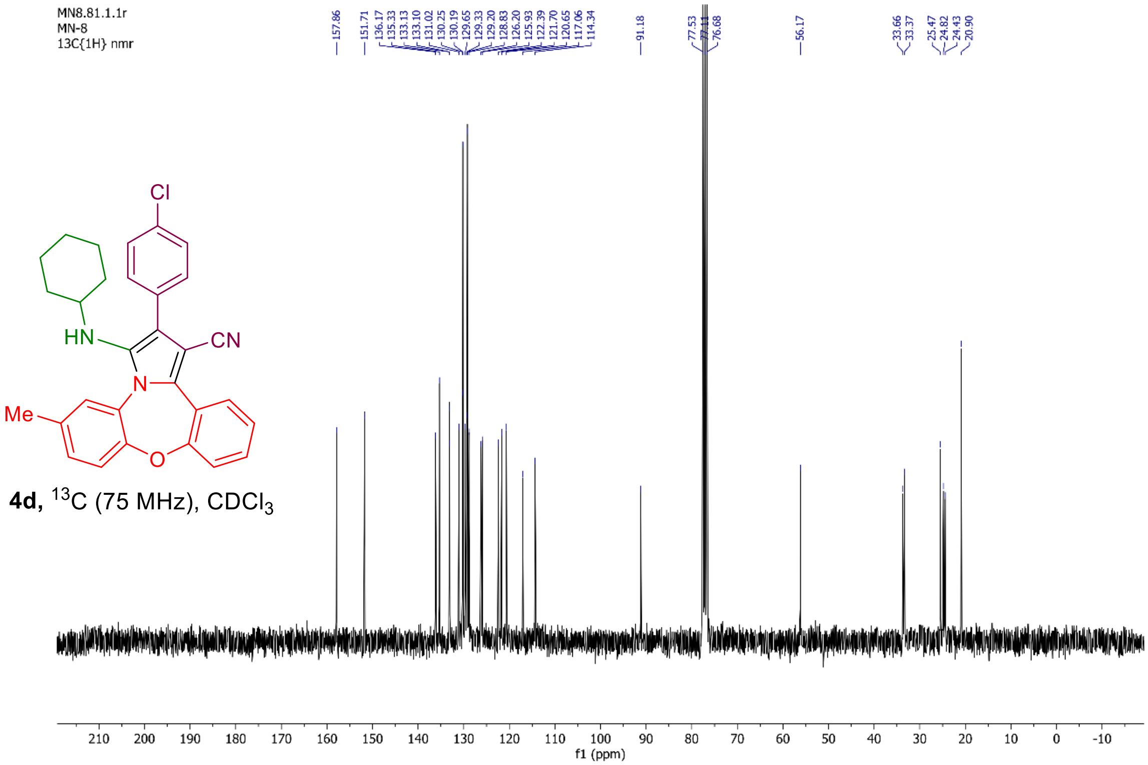

4d, ^{13}C (75 MHz), $CDCl_3$

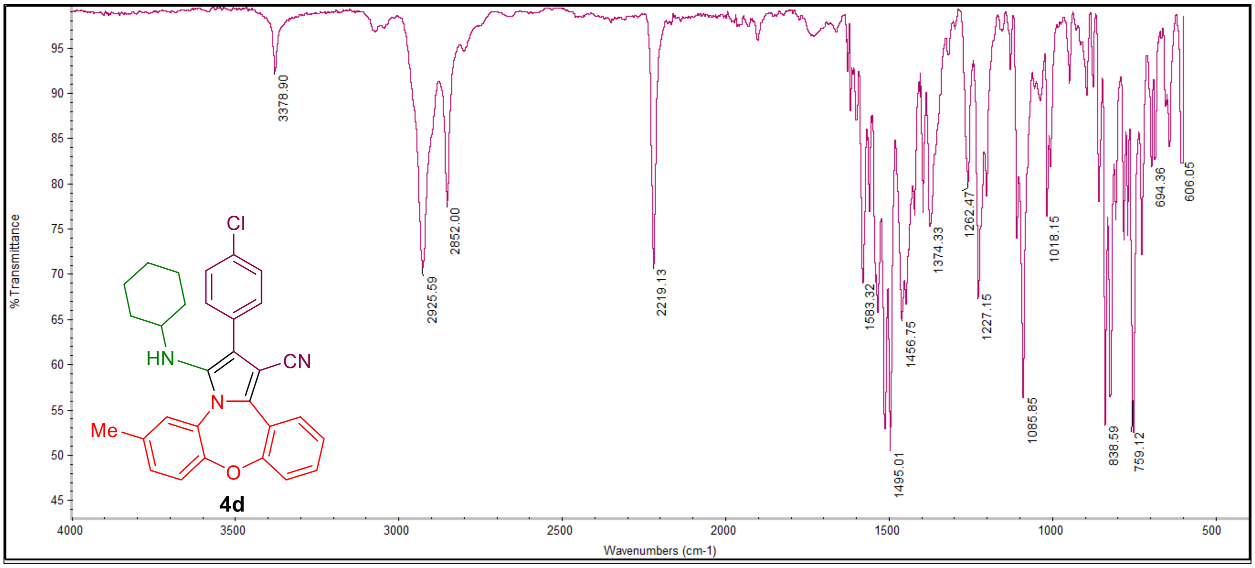
% Transmittance
Wavenumbers (cm-1)
3378.90
2925.59
2852.00
2219.13
1583.32
1495.01
1456.75
1374.33
1262.47
1227.15
1085.85
1018.15
838.59
759.12
694.36
606.05
Cl
HN
CN
N
Me
O
4d

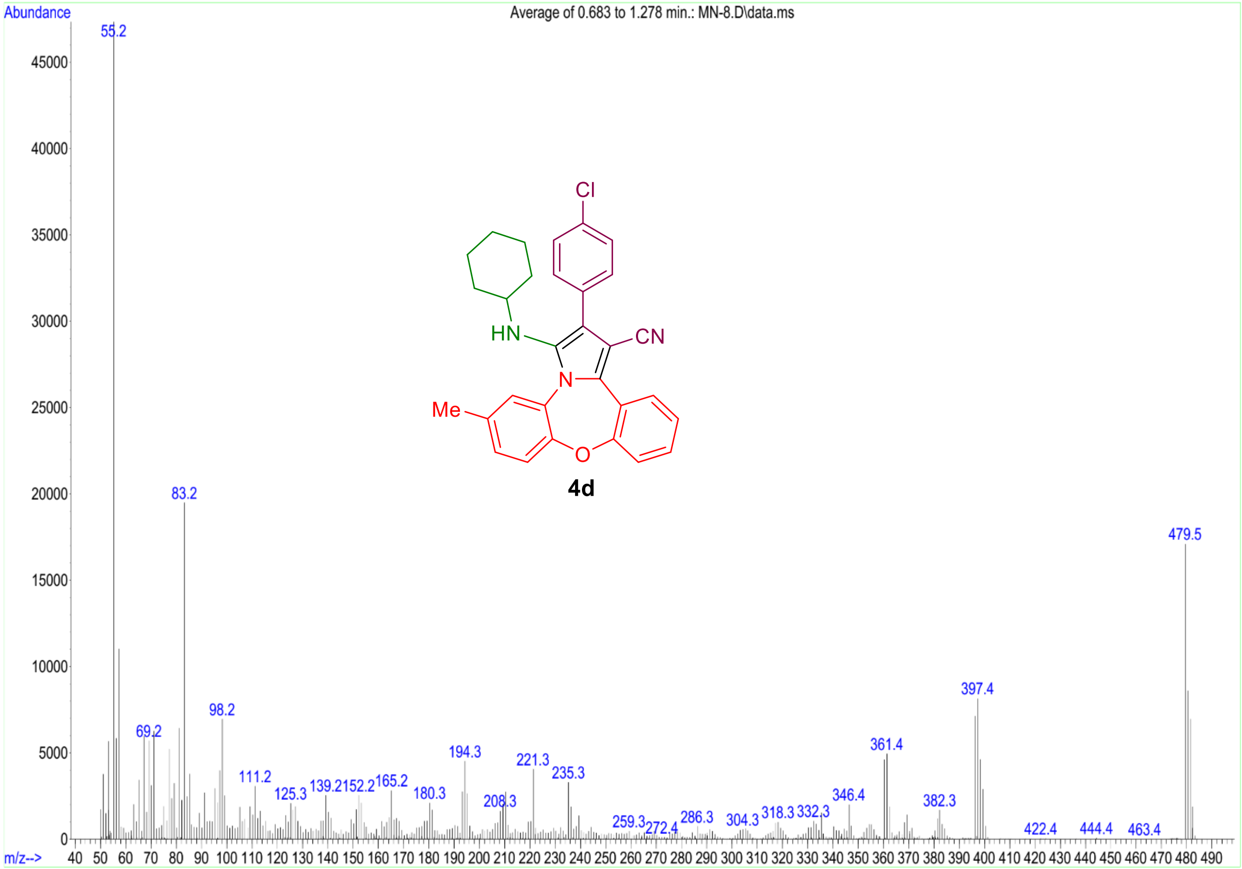
Abundance
Average of 0.683 to 1.278 min.: MN-8.D\data.ms
m/z-->
55.2
83.2
69.2
98.2
111.2
125.3
139.2
152.2
165.2
180.3
194.3
208.3
221.3
235.3
259.3
272.4
286.3
304.3
318.3
332.3
346.4
361.4
382.3
397.4
422.4
444.4
463.4
479.5
Cl
HN
CN
N
Me
O
4d

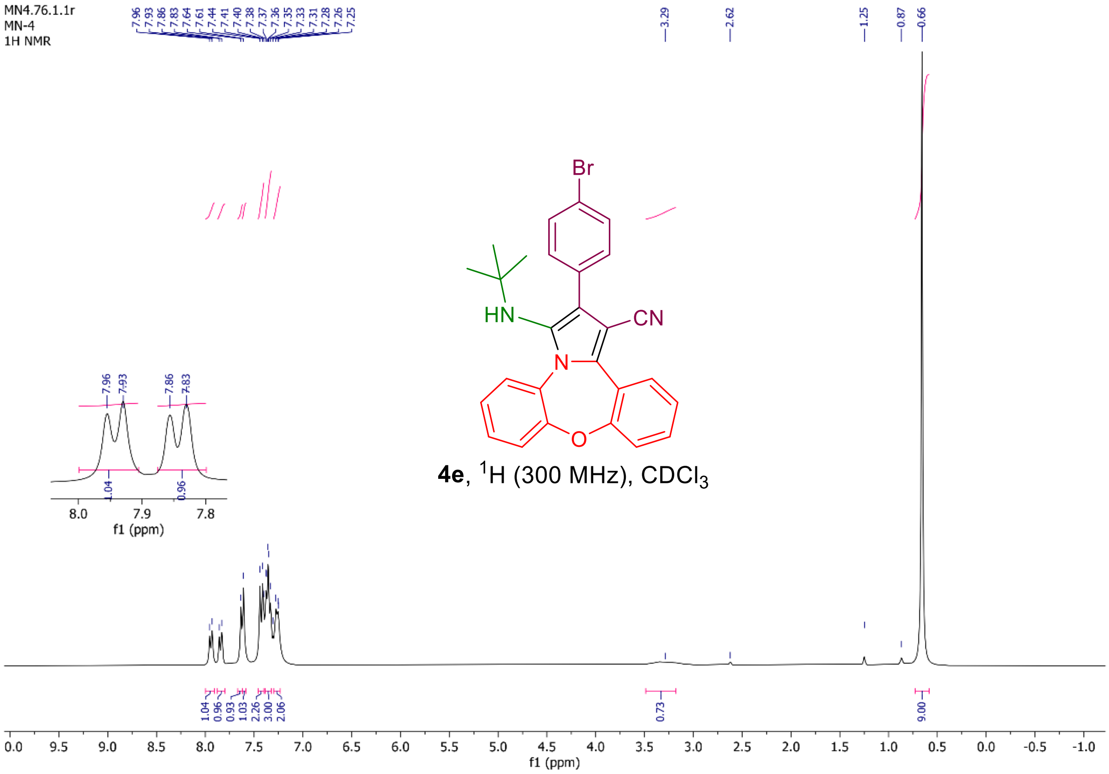
MN4.76.1.1r
MN-4
1H NMR
4e, ¹H (300 MHz), CDCl₃
f1 (ppm)

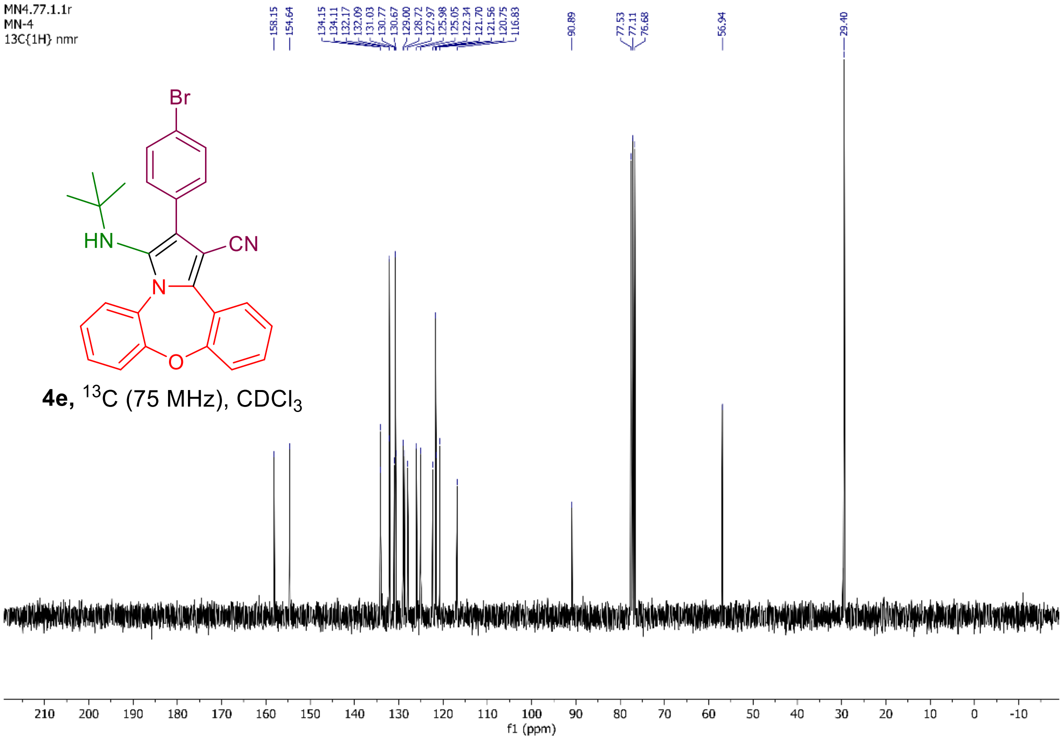
MN4.77.1.1r
MN-4
13C{1H} nmr
4e, ¹³C (75 MHz), CDCl₃
f1 (ppm)

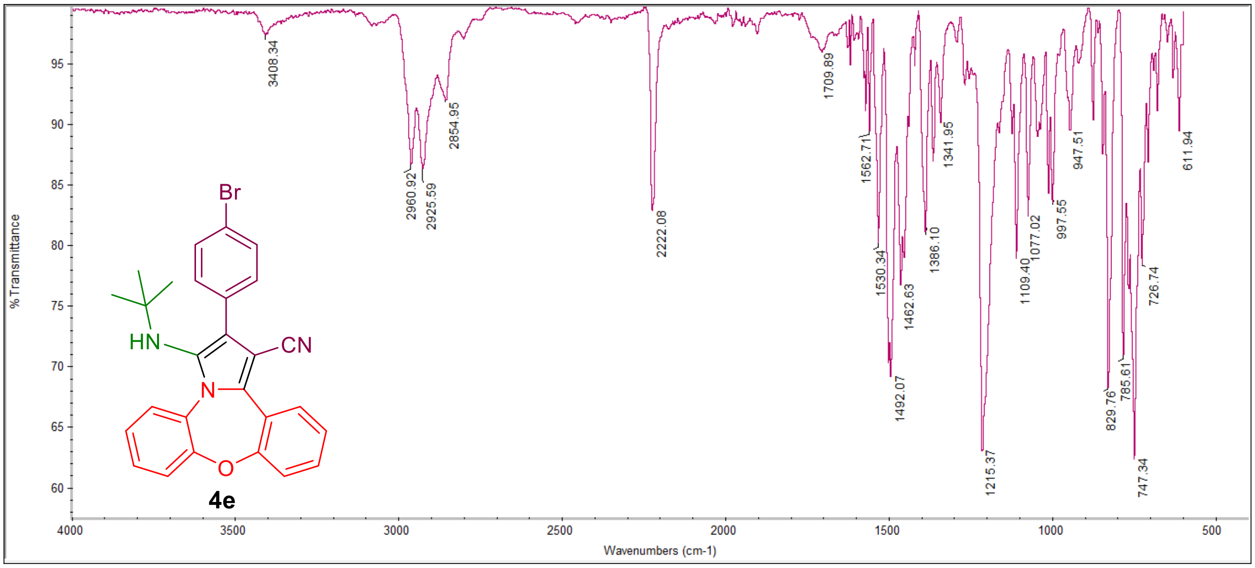
% Transmittance
Wavenumbers (cm-1)
3408.34
2960.92
2925.59
2854.95
2222.08
1709.89
1562.71
1530.34
1492.07
1462.63
1386.10
1341.95
1215.37
1109.40
1077.02
997.55
947.51
829.76
785.61
747.34
726.74
611.94
Br
HN
CN
N
O
4e

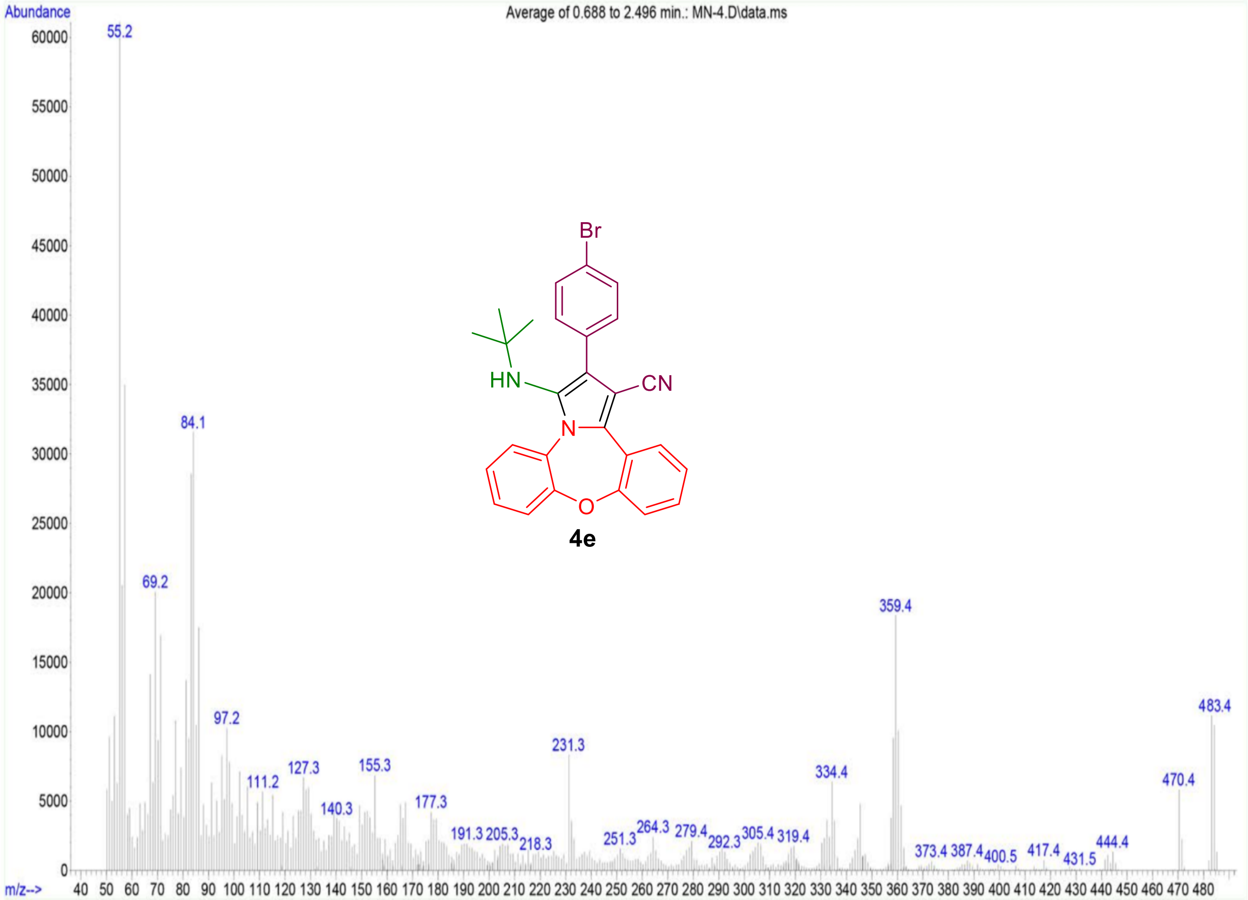
Abundance
Average of 0.688 to 2.496 min.: MN-4.D\data.ms
m/z-->
55.2
84.1
69.2
97.2
111.2
127.3
140.3
155.3
177.3
191.3
205.3
218.3
231.3
251.3
264.3
279.4
292.3
305.4
319.4
334.4
359.4
373.4
387.4
400.5
417.4
431.5
444.4
470.4
483.4
Br
HN
CN
N
O
4e

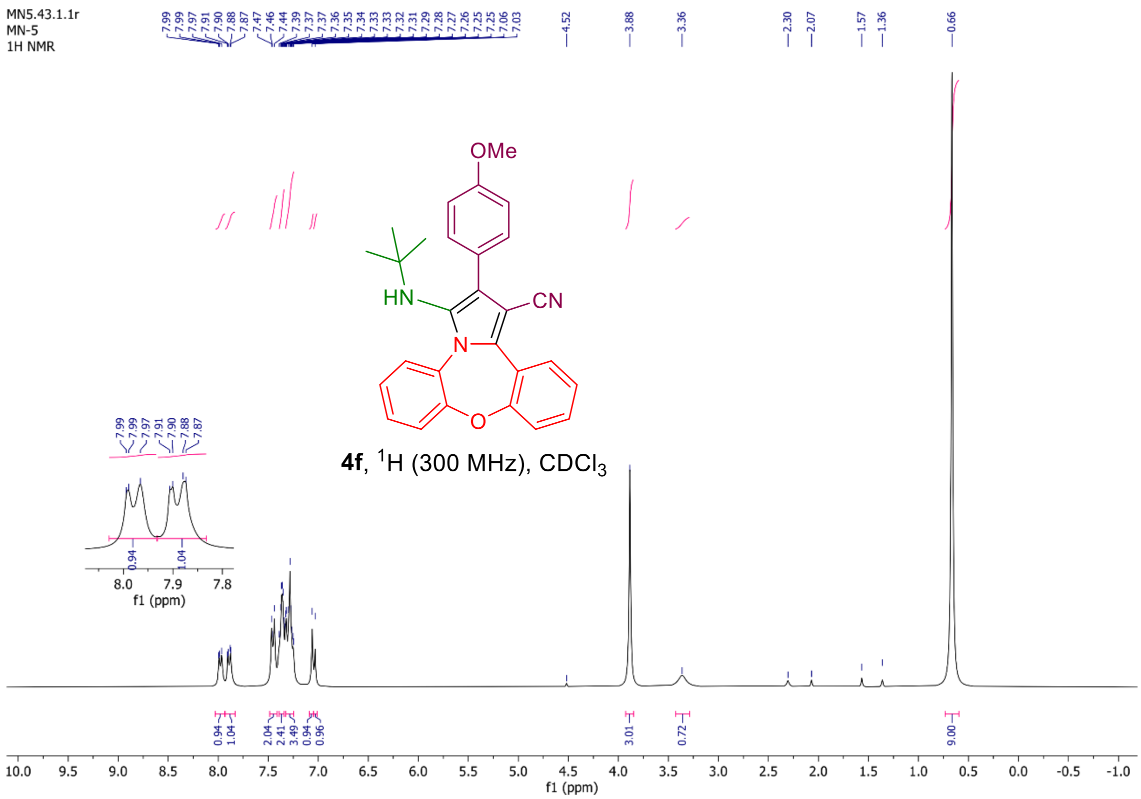
MN5.43.1.1r
MN-5
1H NMR
OMe
HN
CN
N
O
4f, ¹H (300 MHz), CDCl₃
f1 (ppm)

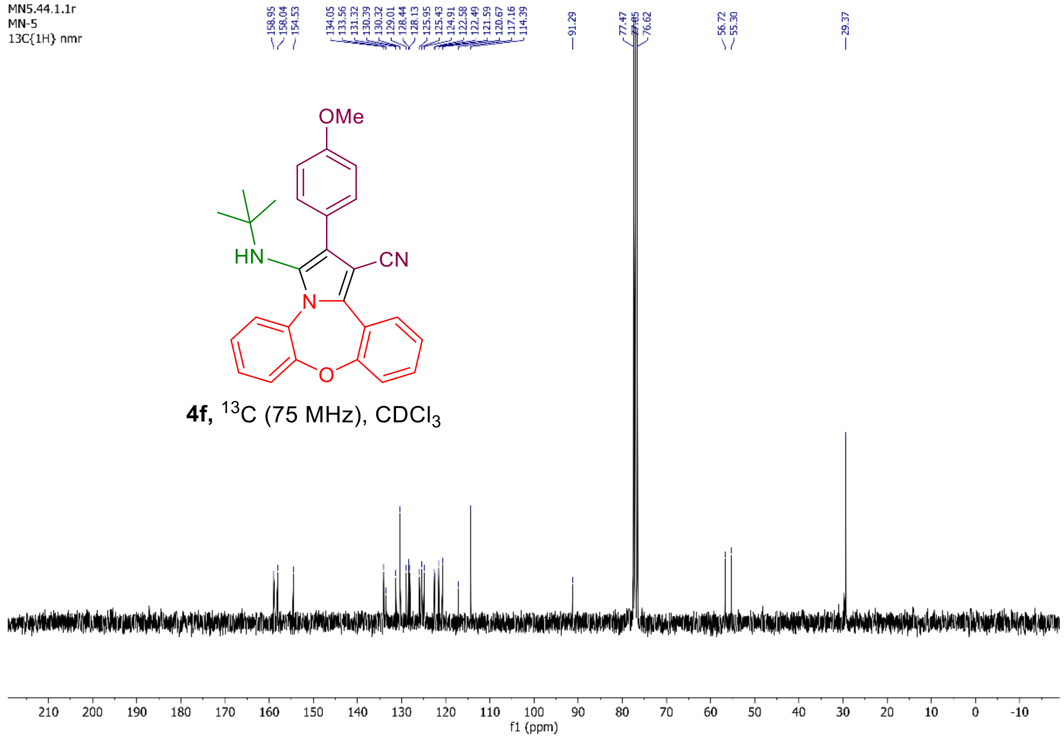
MN5.44.1.1r
MN-5
13C{1H} nmr
OMe
HN
CN
N
O
4f, ¹³C (75 MHz), CDCl₃
f1 (ppm)

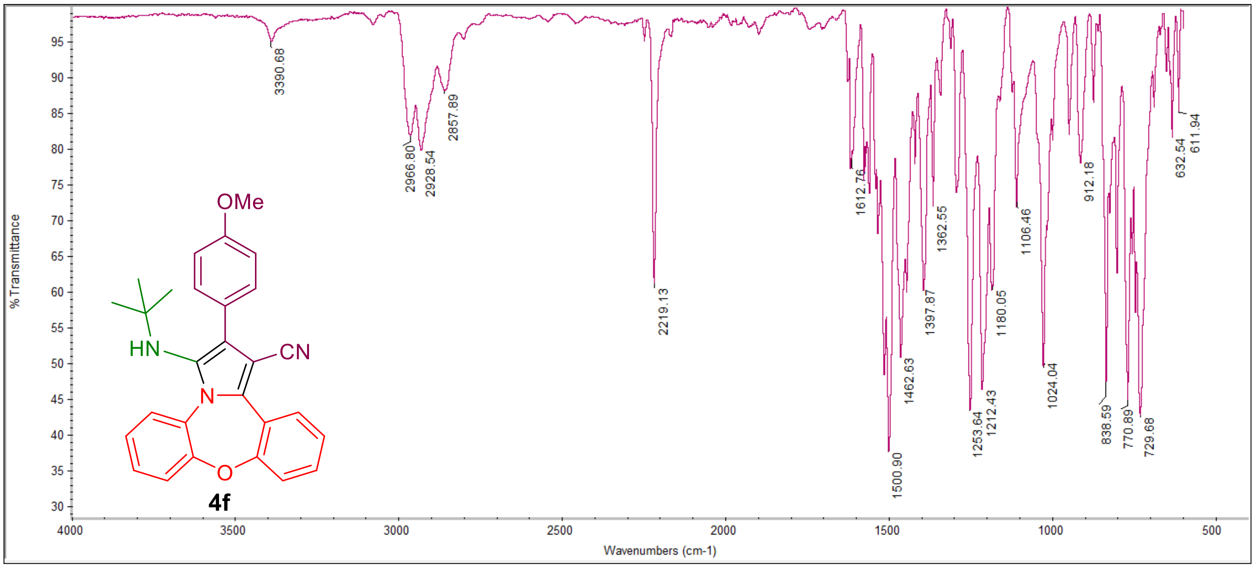
% Transmittance
Wavenumbers (cm-1)
3390.68
2966.80
2928.54
2857.89
2219.13
1612.76
1500.90
1462.63
1397.87
1362.55
1253.64
1212.43
1180.05
1106.46
1024.04
912.18
838.59
770.89
729.68
632.54
611.94
OMe
HN
CN
N
O
4f

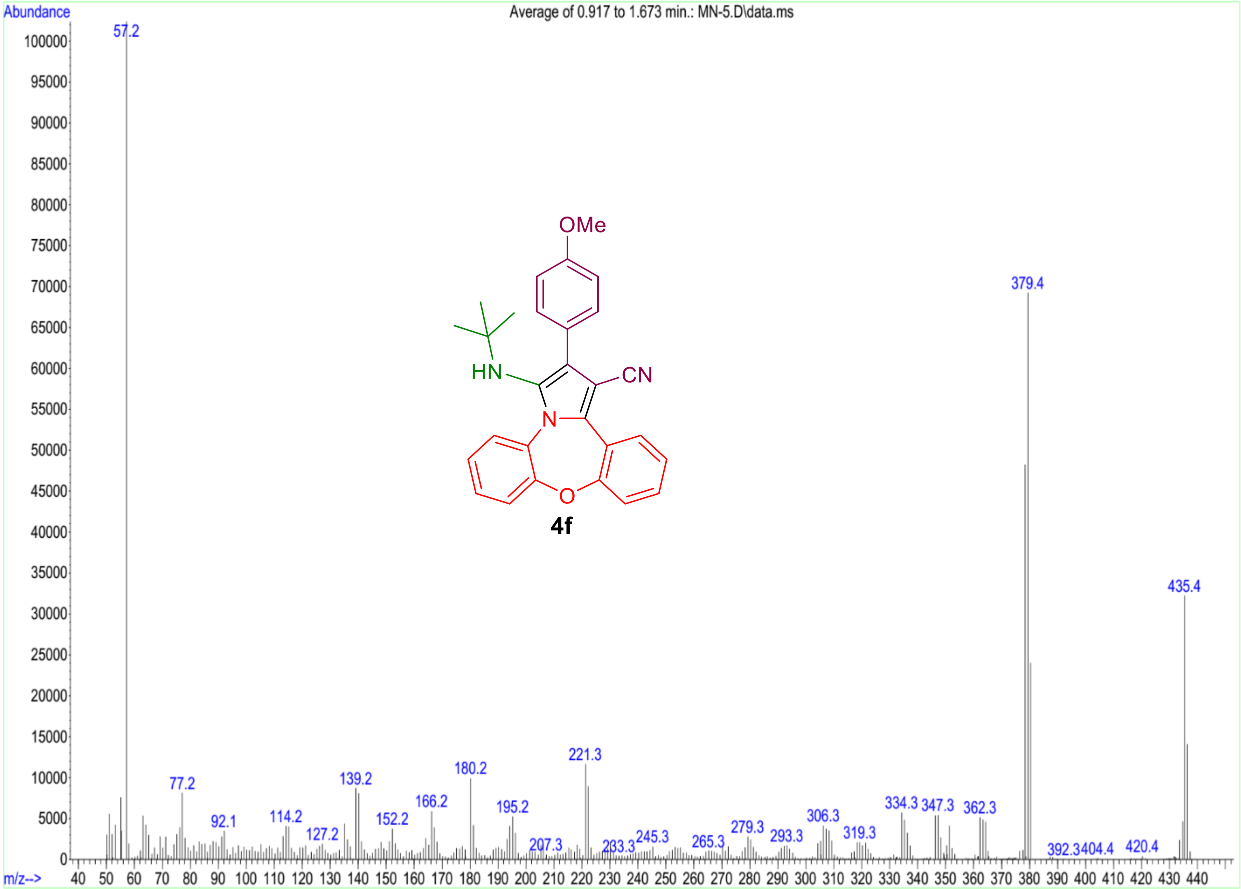
Abundance
Average of 0.917 to 1.673 min.: MN-5.D\data.ms
m/z-->
57.2
77.2
92.1
114.2
127.2
139.2
152.2
166.2
180.2
195.2
207.3
221.3
233.3
245.3
265.3
279.3
293.3
306.3
319.3
334.3
347.3
362.3
379.4
392.3
404.4
420.4
435.4
OMe
HN
CN
N
O
4f

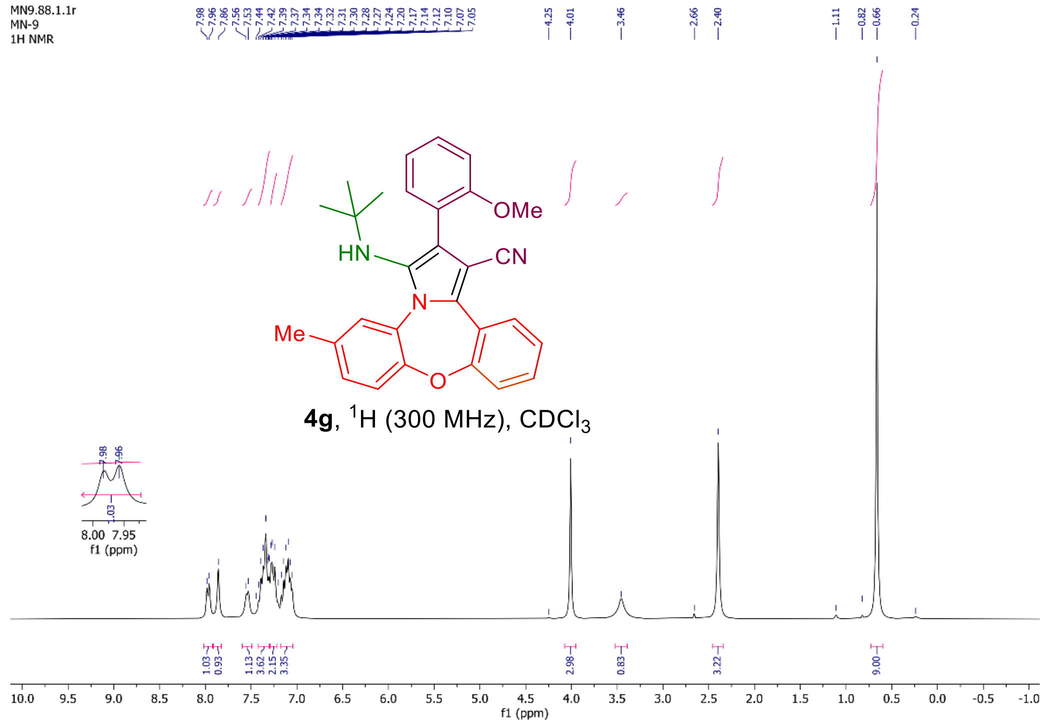
MN9.88.1.1r
MN-9
1H NMR
OMe
HN
CN
N
Me
O
4g, ¹H (300 MHz), CDCl₃
f1 (ppm)

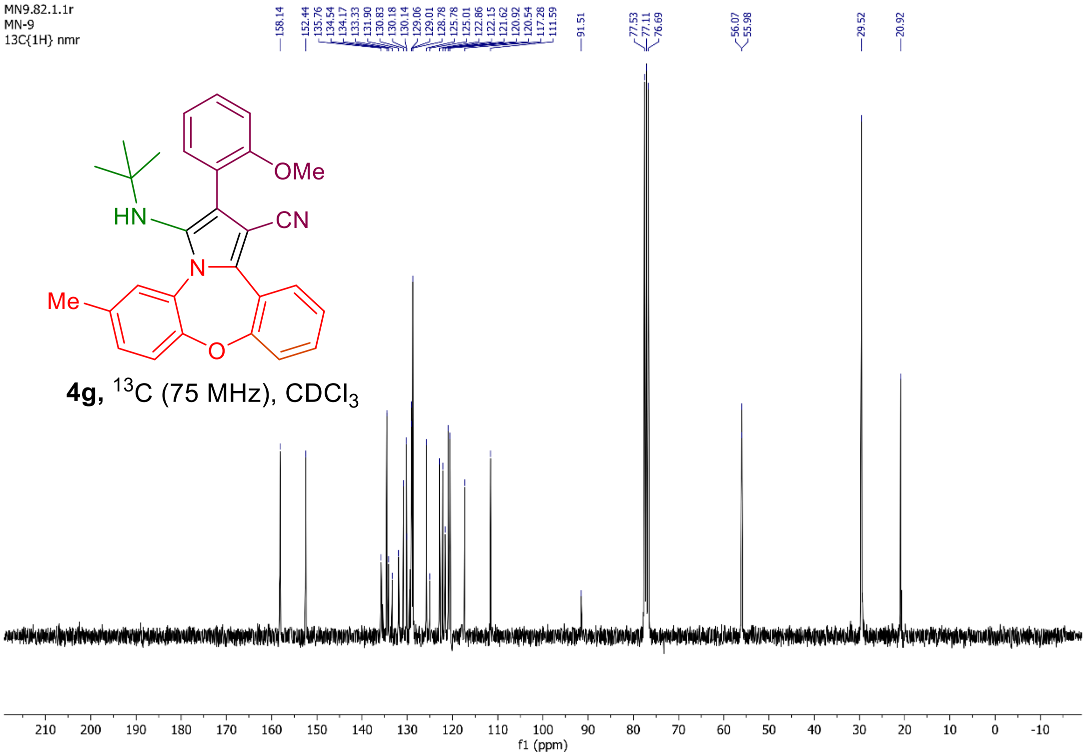
MN9.82.1.1r
MN-9
13C{1H} nmr
OMe
HN
CN
N
Me
O
4g, ¹³C (75 MHz), CDCl₃
f1 (ppm)

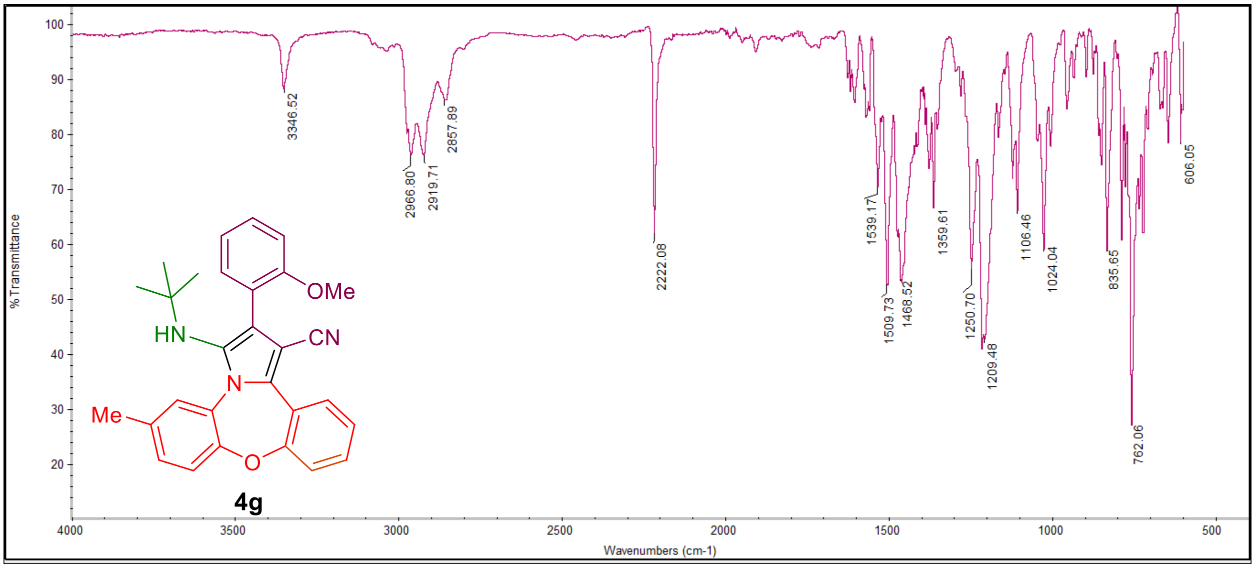

% Transmittance
Wavenumbers (cm-1)
3346.52
2966.80
2919.71
2857.89
2222.08
1539.17
1509.73
1468.52
1359.61
1250.70
1209.48
1106.46
1024.04
835.65
762.06
606.05
OMe
HN
CN
N
Me
O
4g

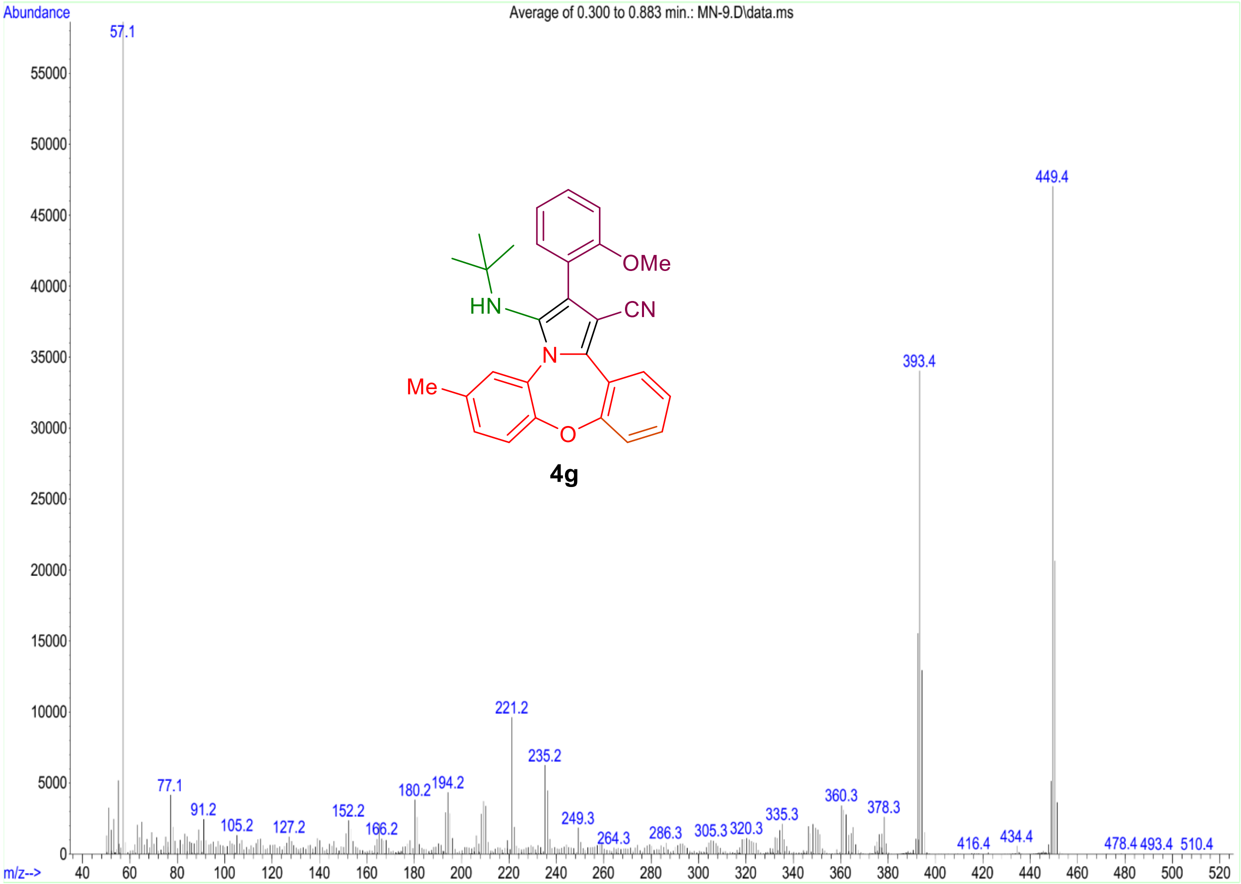

Abundance
Average of 0.300 to 0.883 min.: MN-9.D\data.ms
m/z-->
57.1
449.4
393.4
221.2
235.2
77.1
91.2
105.2
127.2
152.2
166.2
180.2
194.2
249.3
264.3
286.3
305.3
320.3
335.3
360.3
378.3
416.4
434.4
478.4
493.4
510.4
OMe
HN
CN
N
Me
O
4g

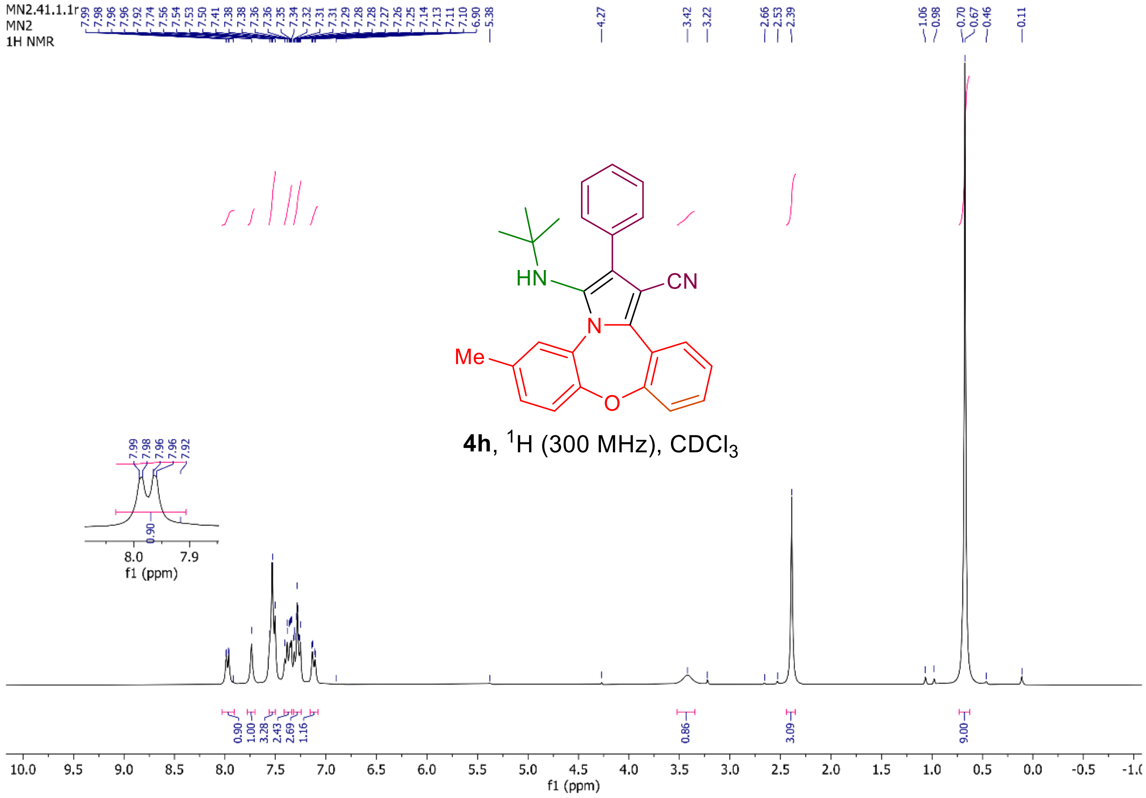
MN2.41.1.1r
MN2
1H NMR
4h, ^{1}H (300 MHz), CDCl$_3$
f1 (ppm)

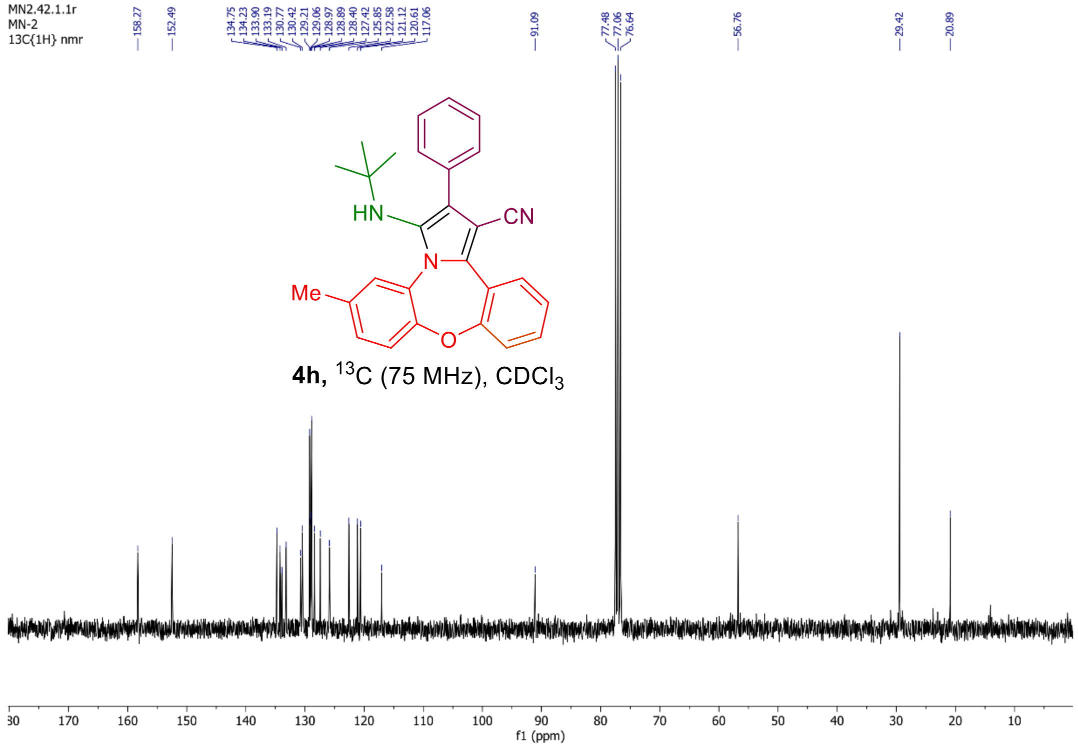
MN2.42.1.1r
MN-2
13C{1H} nmr
4h, ^{13}C (75 MHz), CDCl$_3$
f1 (ppm)

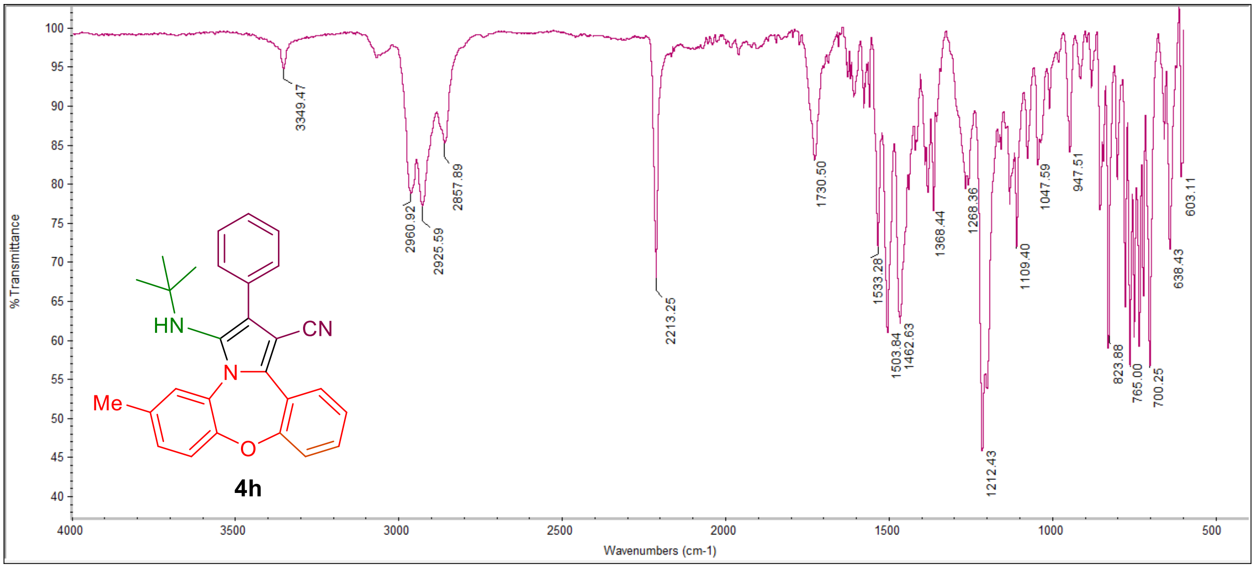
% Transmittance
Wavenumbers (cm-1)
3349.47
2960.92
2925.59
2857.89
2213.25
1730.50
1533.28
1503.84
1462.63
1368.44
1268.36
1212.43
1109.40
1047.59
947.51
823.88
765.00
700.25
638.43
603.11
HN
CN
N
Me
O
4h

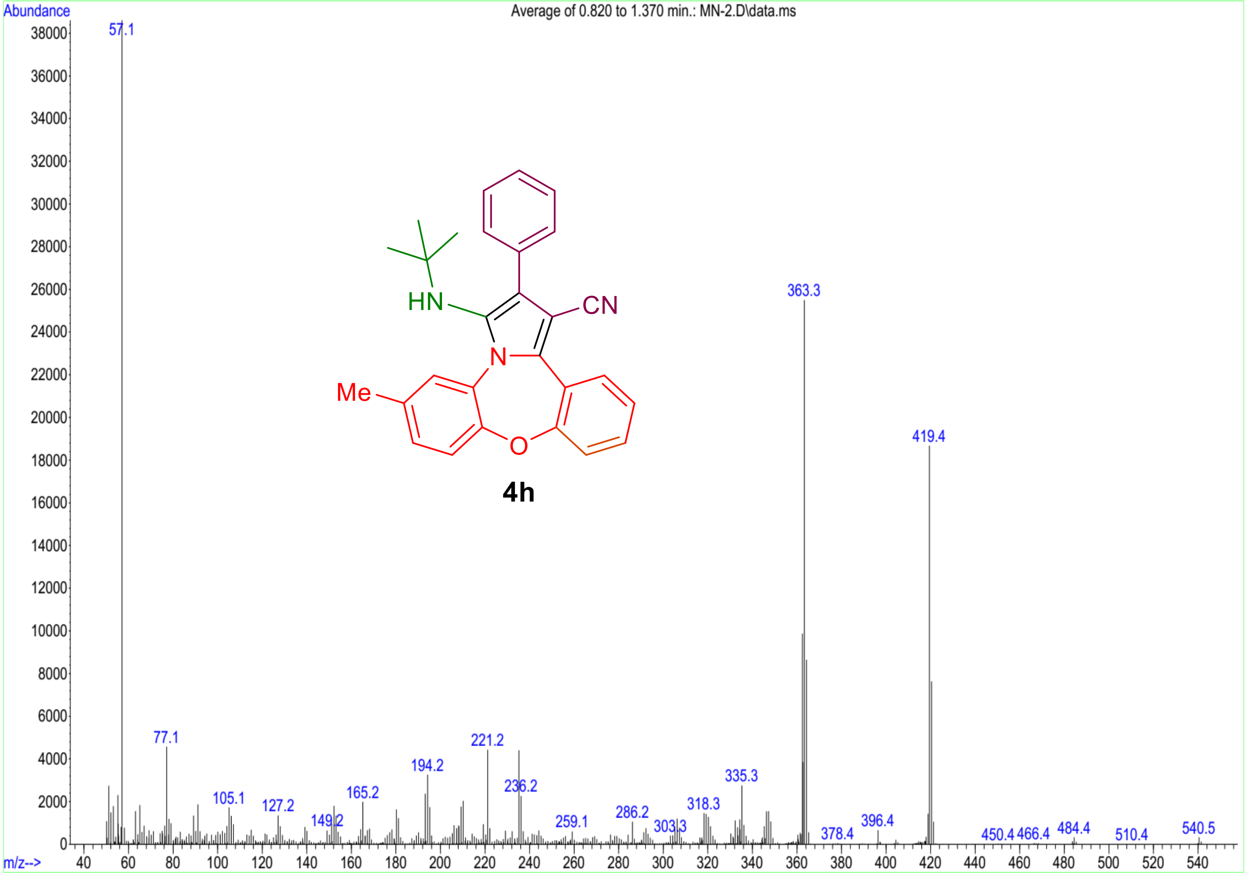
Abundance
Average of 0.820 to 1.370 min.: MN-2.D\data.ms
m/z-->
57.1
77.1
105.1
127.2
149.2
165.2
194.2
221.2
236.2
259.1
286.2
303.3
318.3
335.3
363.3
378.4
396.4
419.4
450.4
466.4
484.4
510.4
540.5
HN
CN
N
Me
O
4h

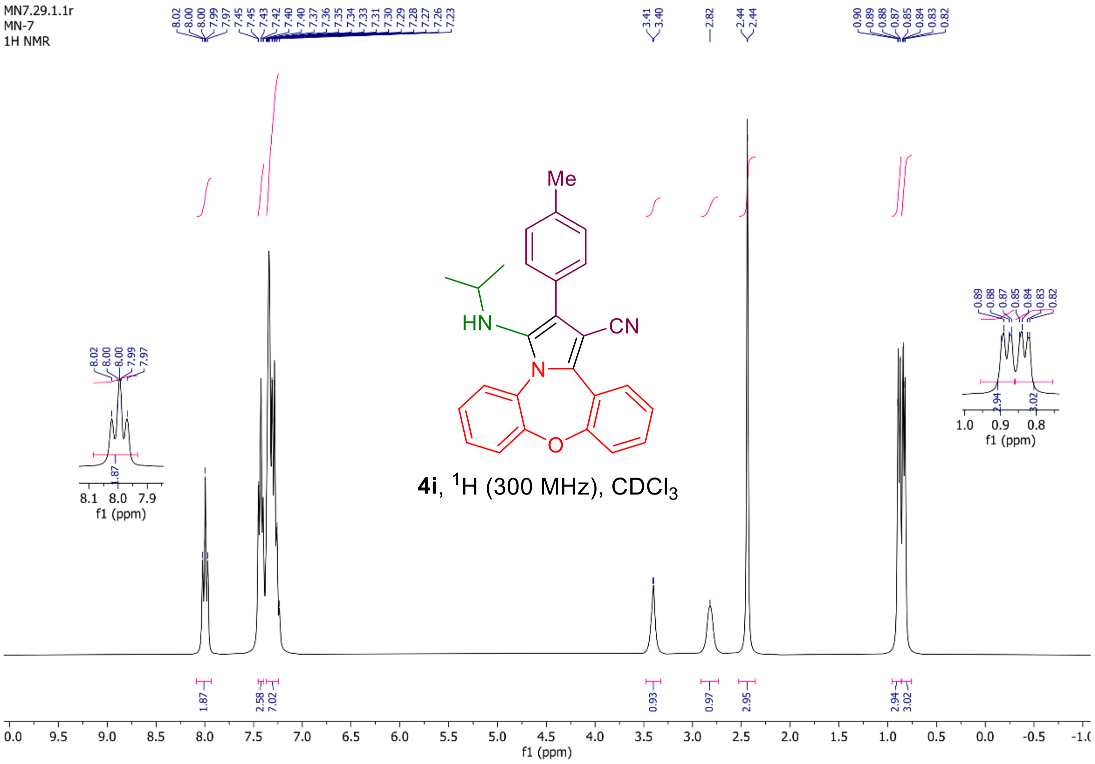
MN7.29.1.1r
MN-7
1H NMR
4i, ¹H (300 MHz), CDCl₃
f1 (ppm)

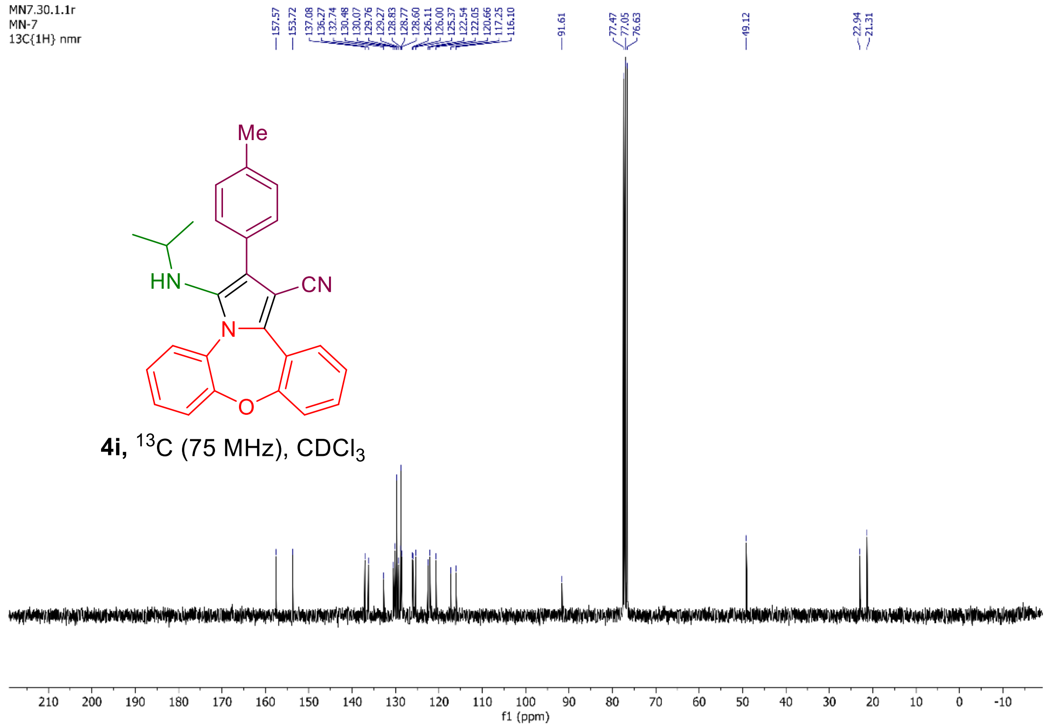
MN7.30.1.1r
MN-7
13C{1H} nmr
4i, ¹³C (75 MHz), CDCl₃
f1 (ppm)

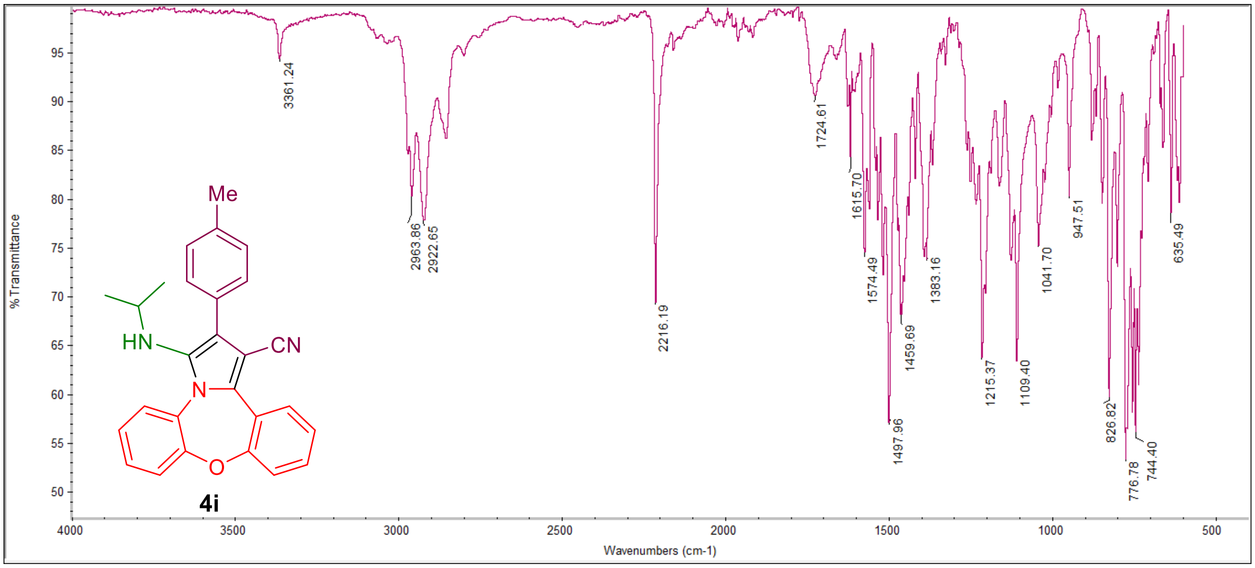
% Transmittance
Wavenumbers (cm-1)
3361.24
2963.86
2922.65
2216.19
1724.61
1615.70
1574.49
1497.96
1459.69
1383.16
1215.37
1109.40
1041.70
947.51
826.82
776.78
744.40
635.49
Me
HN
CN
N
O
4i

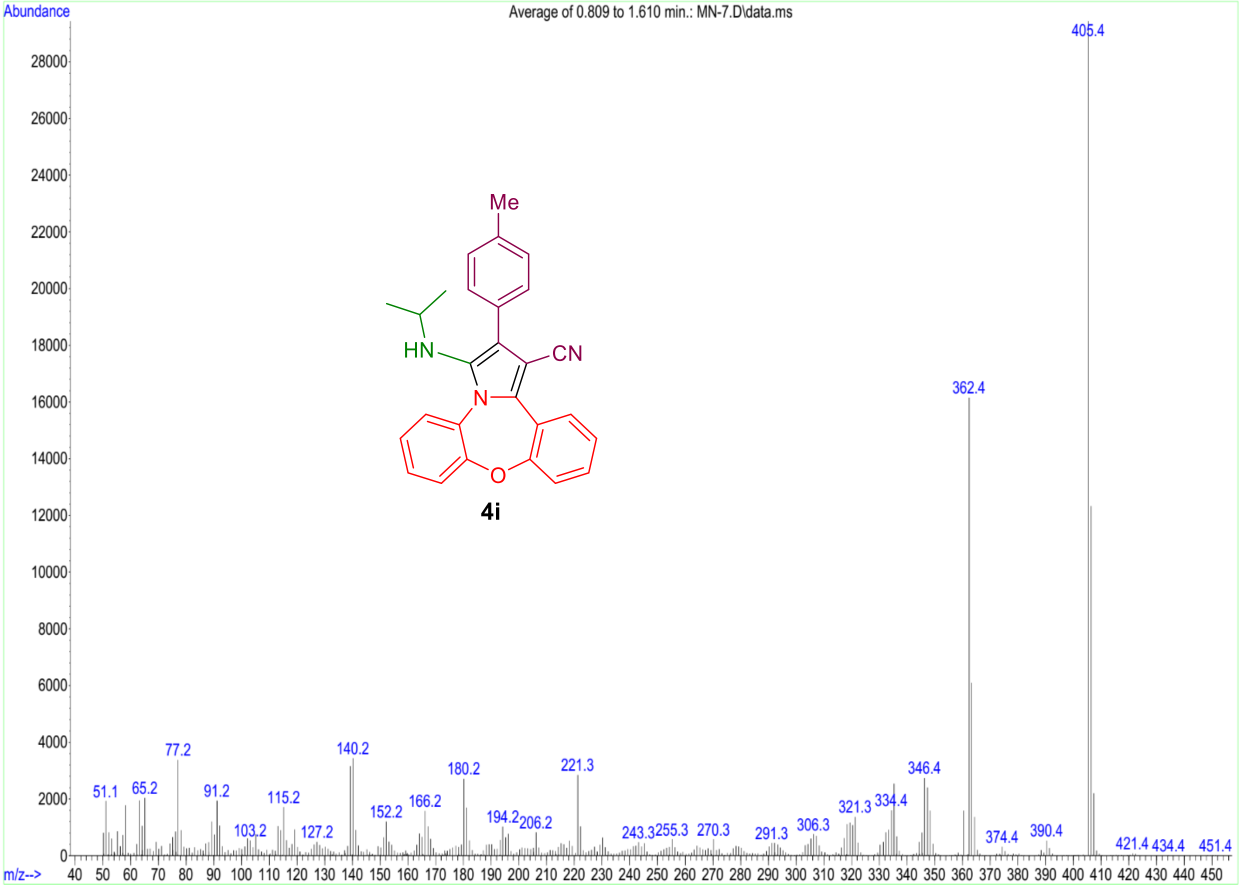
Abundance
Average of 0.809 to 1.610 min.: MN-7.D\data.ms
m/z-->
405.4
362.4
346.4
334.4
321.3
306.3
291.3
270.3
255.3
243.3
221.3
206.2
194.2
180.2
166.2
152.2
140.2
127.2
115.2
103.2
91.2
77.2
65.2
51.1
374.4
390.4
421.4
434.4
451.4
Me
HN
CN
N
O
4i

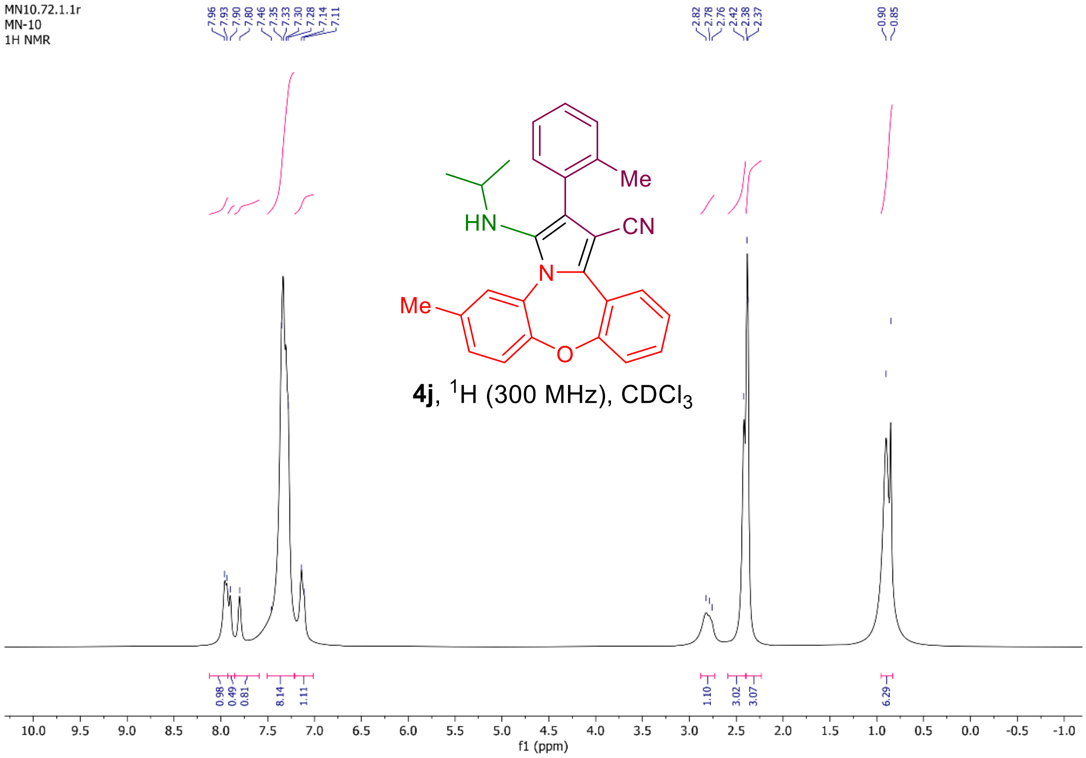
MN10.72.1.1r
MN-10
1H NMR
7.96 7.93 7.90 7.80 7.46 7.35 7.33 7.30 7.28 7.14 7.11
2.82 2.78 2.76 2.42 2.38 2.37
0.90 0.85
Me
HN
CN
N
Me
O
4j, 1H (300 MHz), CDCl3
0.98 0.49 0.81 8.14 1.11 1.10 3.02 3.07 6.29
f1 (ppm)

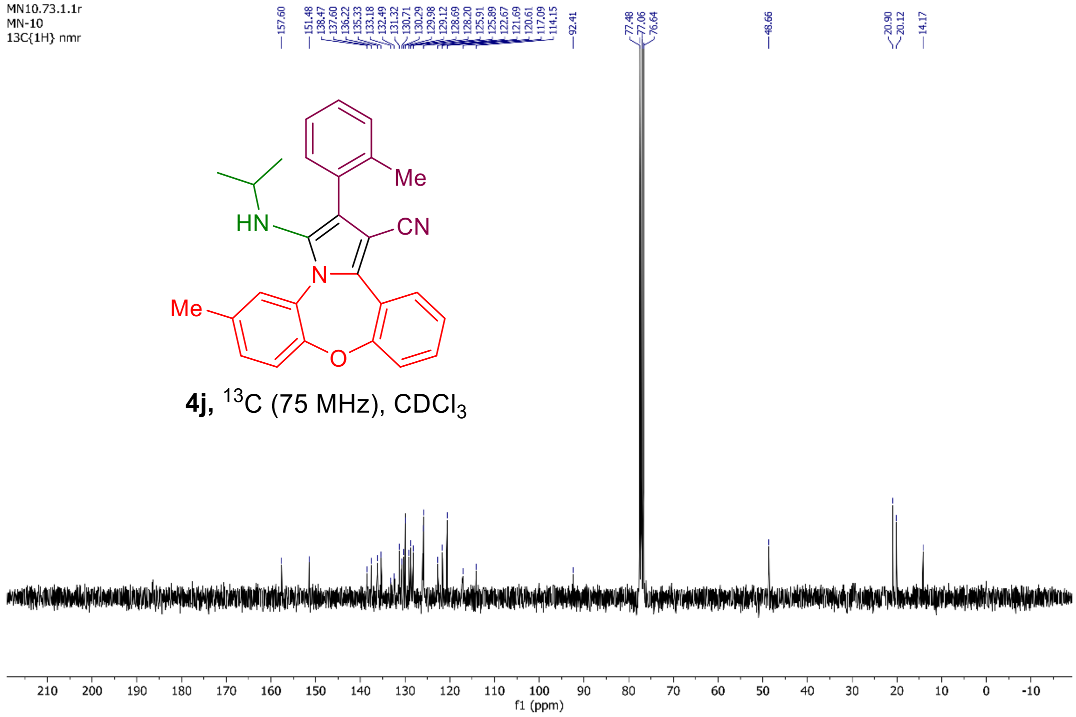
MN10.73.1.1r
MN-10
13C{1H} nmr
157.60
151.48 138.47 137.60 136.22 135.33 133.18 132.49 131.32 130.71 130.29 129.98 129.12 128.69 128.20 125.91 125.89 122.67 121.69 120.61 117.09 114.15
92.41
77.48 77.06 76.64
48.66
20.90 20.12
14.17
Me
HN
CN
N
Me
O
4j, 13C (75 MHz), CDCl3
f1 (ppm)

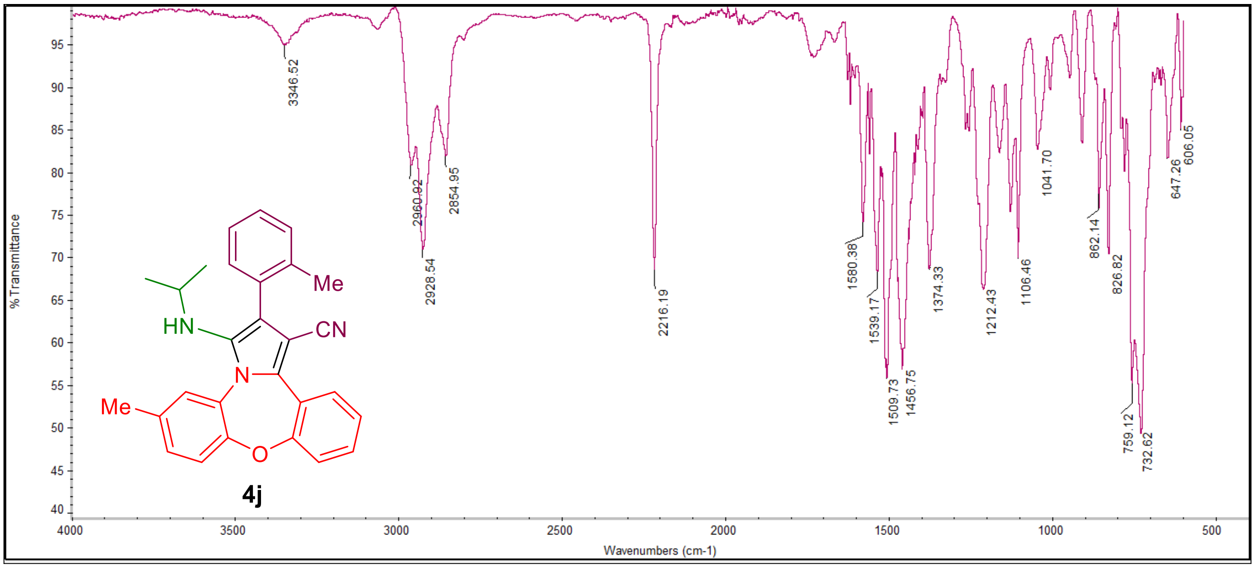

% Transmittance
Wavenumbers (cm-1)
3346.52
2960.92
2928.54
2854.95
2216.19
1580.38
1539.17
1509.73
1456.75
1374.33
1212.43
1106.46
1041.70
862.14
826.82
759.12
732.62
647.26
606.05
HN
Me
CN
N
O
4j

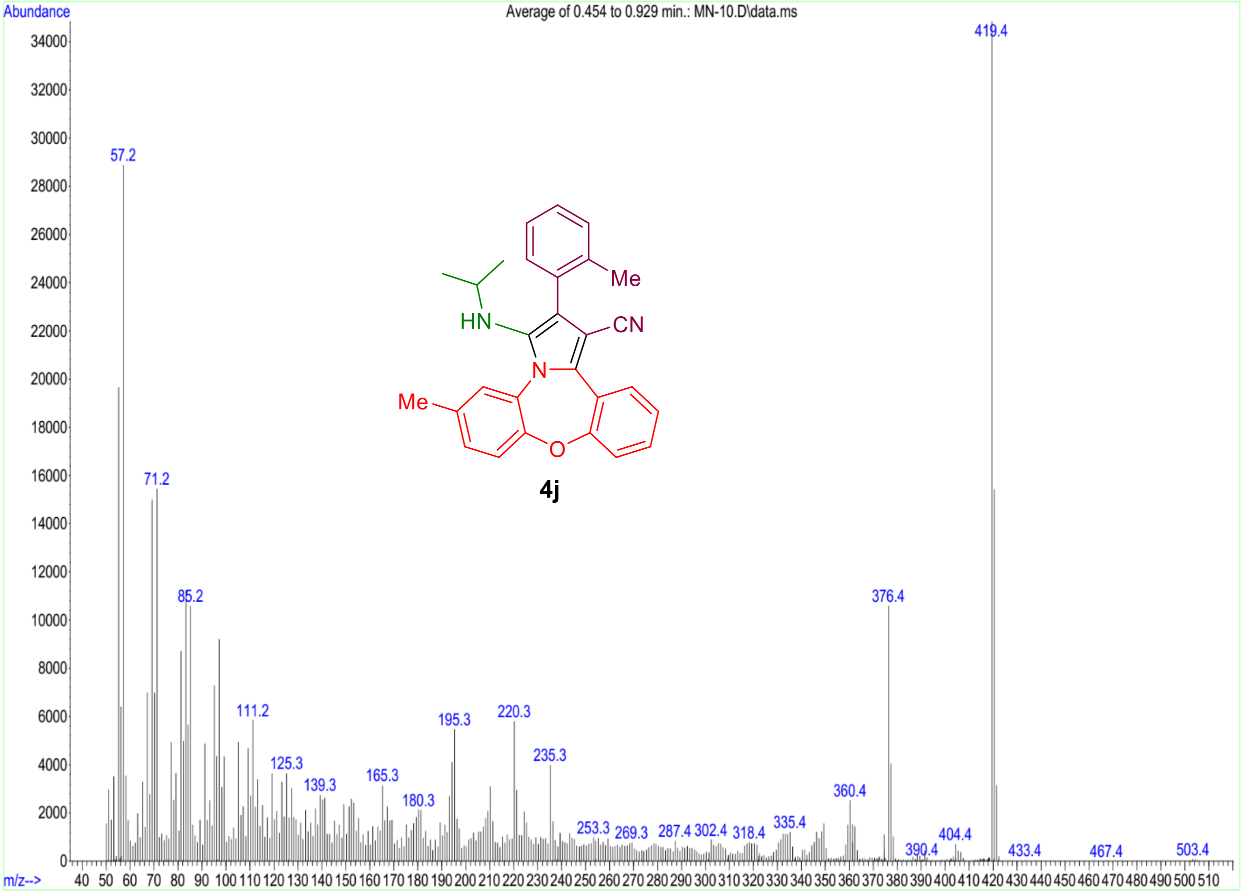

Abundance
Average of 0.454 to 0.929 min.: MN-10.D\data.ms
m/z-->
419.4
57.2
71.2
85.2
111.2
125.3
139.3
165.3
180.3
195.3
220.3
235.3
253.3
269.3
287.4
302.4
318.4
335.4
360.4
376.4
390.4
404.4
433.4
467.4
503.4
HN
Me
CN
N
O
4j

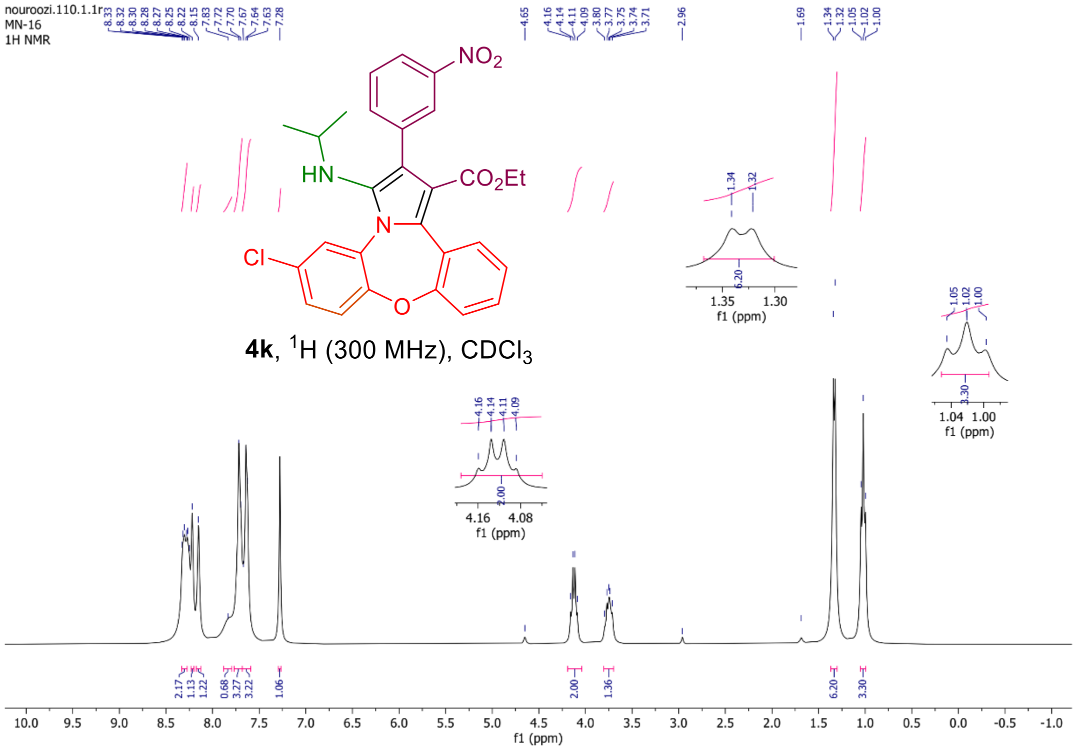
nouroozi.110.1.1r
MN-16
1H NMR
4k, ^{1}H (300 MHz), CDCl$_3$
f1 (ppm)

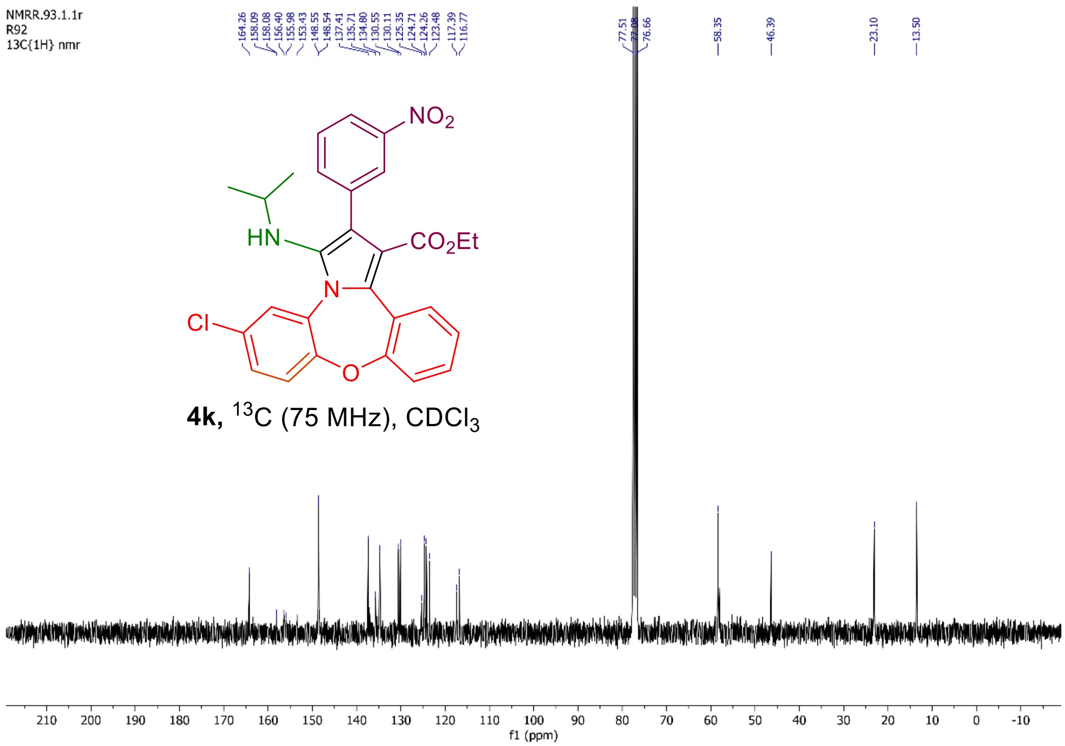
NMRR.93.1.1r
R92
13C{1H} nmr
4k, ^{13}C (75 MHz), CDCl$_3$
f1 (ppm)

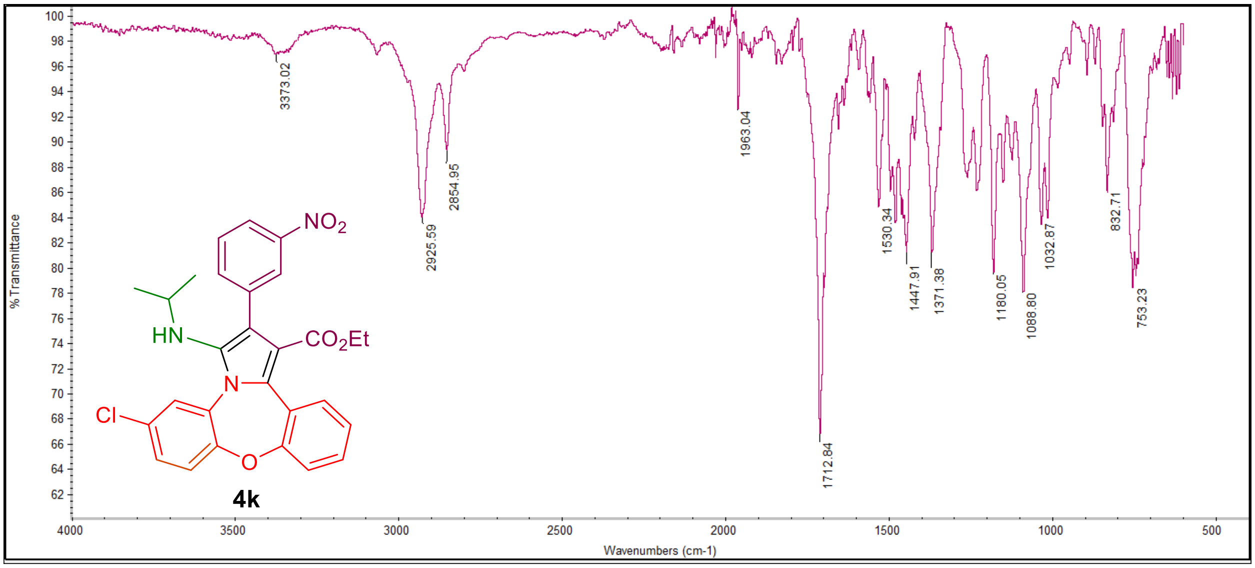

% Transmittance
Wavenumbers (cm-1)
3373.02
2925.59
2854.95
1963.04
1712.84
1530.34
1447.91
1371.38
1180.05
1088.80
1032.87
832.71
753.23
NO2
HN
CO2Et
N
Cl
O
4k

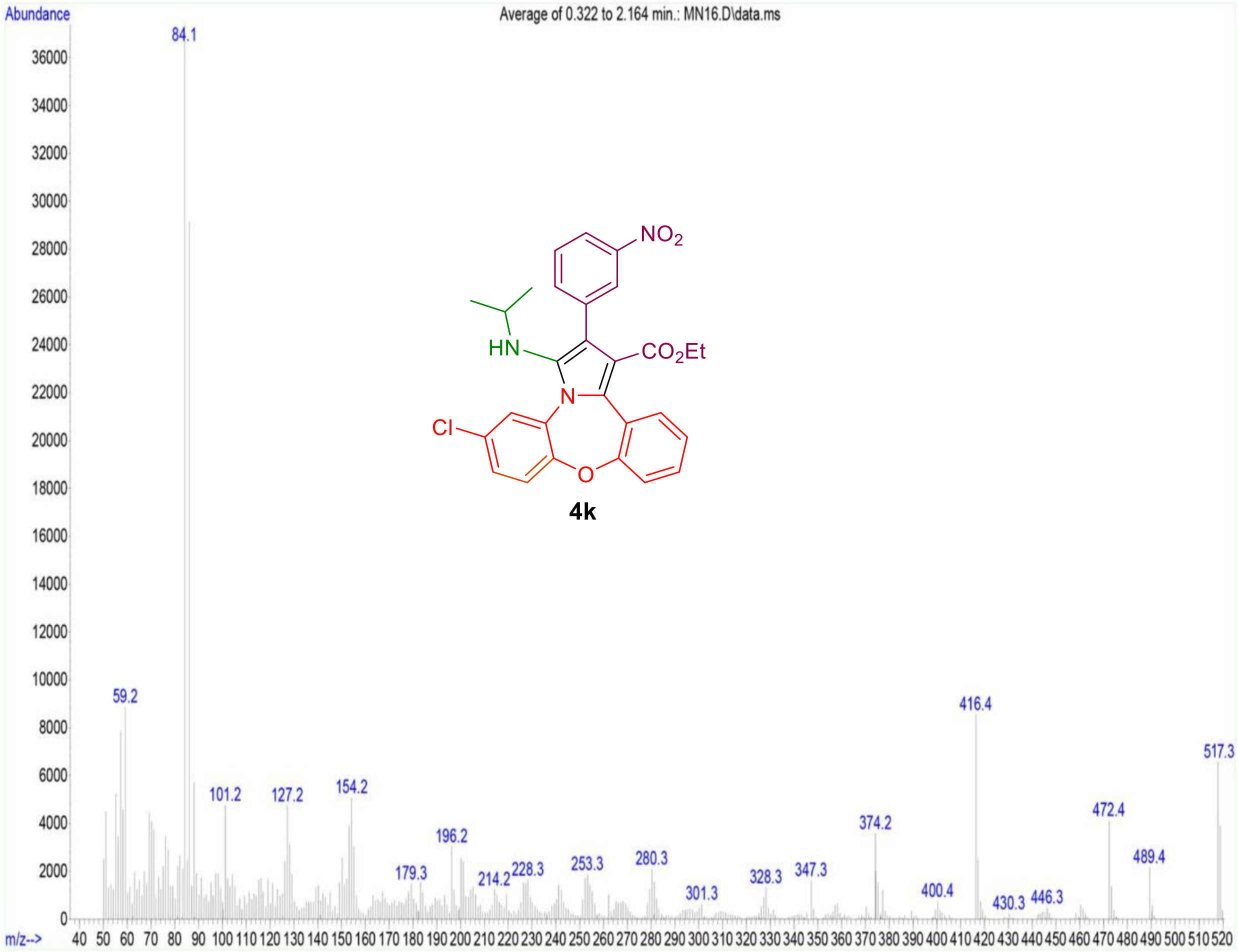

Abundance
Average of 0.322 to 2.164 min.: MN16.D\data.ms
m/z-->
84.1
59.2
101.2
127.2
154.2
179.3
196.2
214.2
228.3
253.3
280.3
301.3
328.3
347.3
374.2
400.4
416.4
430.3
446.3
472.4
489.4
517.3
NO2
HN
CO2Et
N
Cl
O
4k

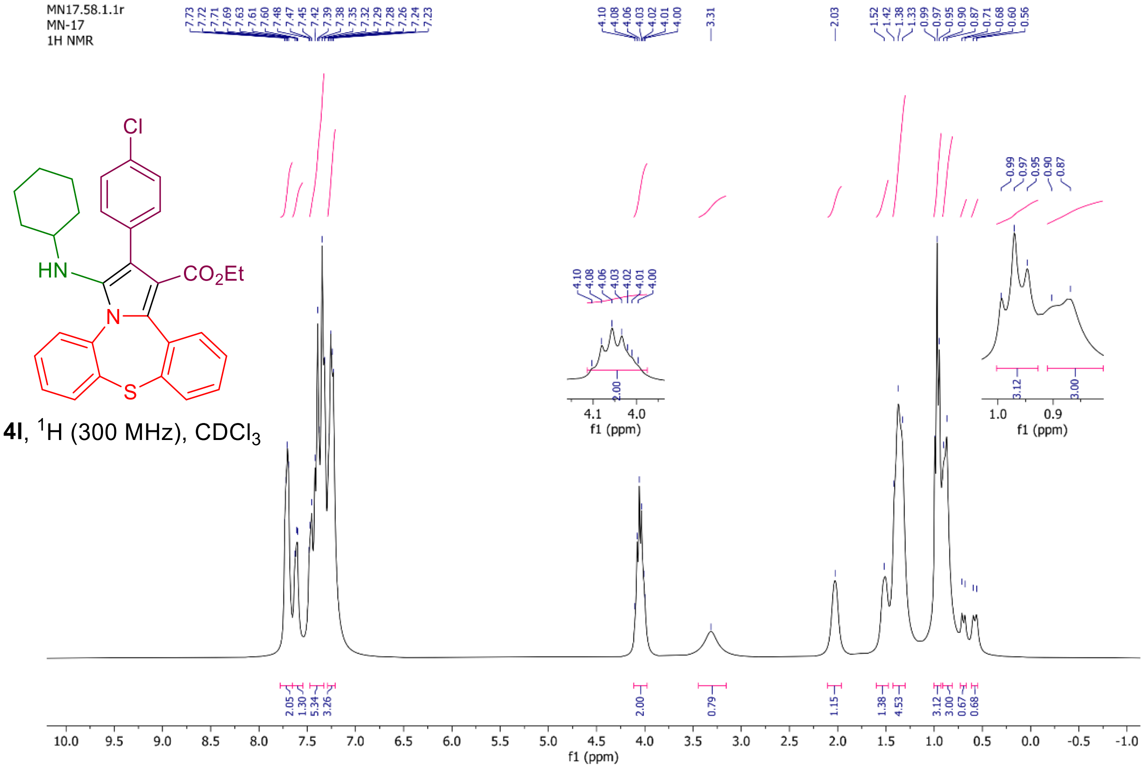
MN17.58.1.1r
MN-17
1H NMR
Cl
HN
CO_2Et
N
S
4l, ^1H (300 MHz), $CDCl_3$
f1 (ppm)

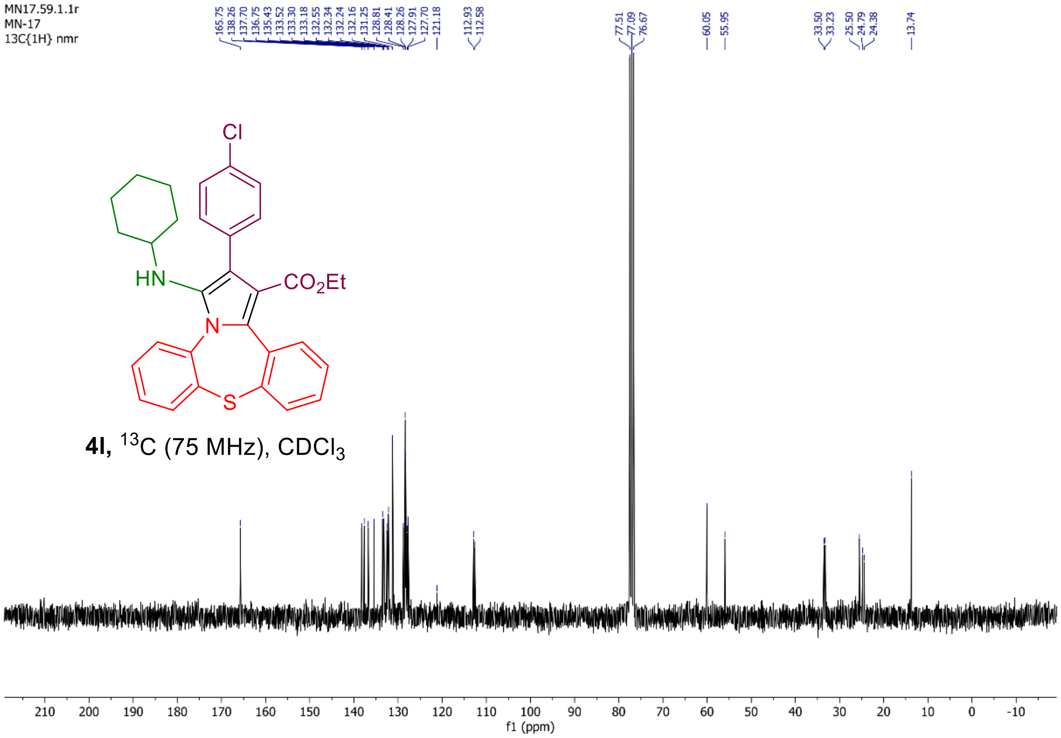
MN17.59.1.1r
MN-17
13C{1H} nmr
Cl
HN
CO_2Et
N
S
4l, ^{13}C (75 MHz), $CDCl_3$
f1 (ppm)

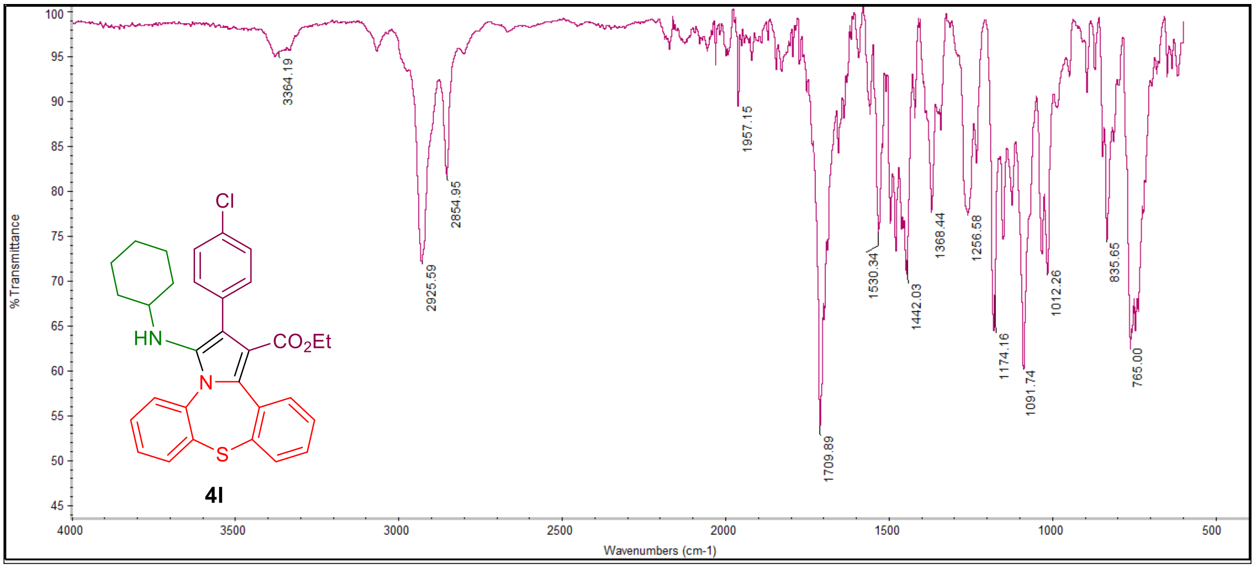
% Transmittance
Wavenumbers (cm-1)
3364.19
2925.59
2854.95
1957.15
1709.89
1530.34
1442.03
1368.44
1256.58
1174.16
1091.74
1012.26
835.65
765.00
Cl
HN
CO2Et
N
S
4l

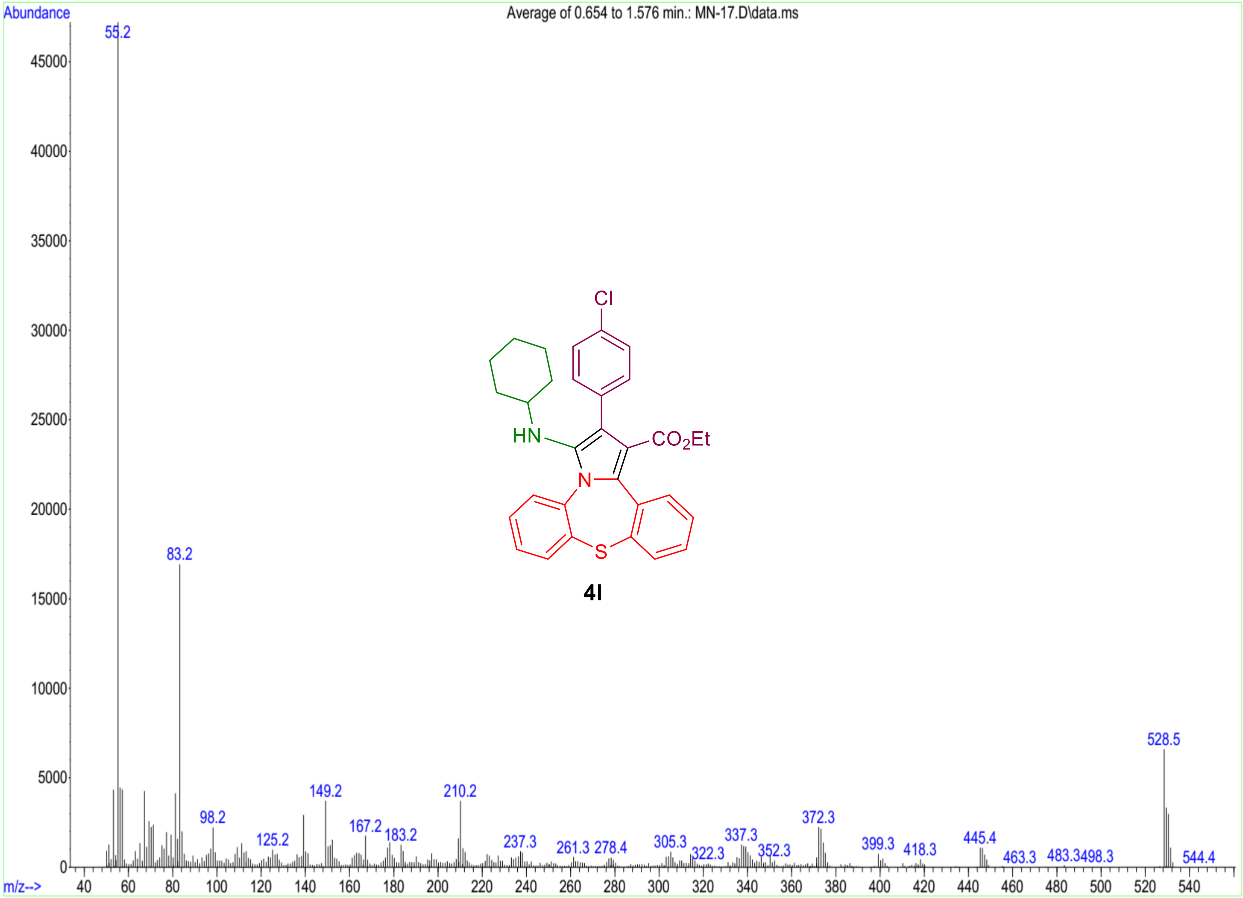
Abundance
Average of 0.654 to 1.576 min.: MN-17.D\data.ms
55.2
83.2
98.2
125.2
149.2
167.2
183.2
210.2
237.3
261.3
278.4
305.3
322.3
337.3
352.3
372.3
399.3
418.3
445.4
463.3
483.3
498.3
528.5
544.4
m/z-->
Cl
HN
CO2Et
N
S
4l

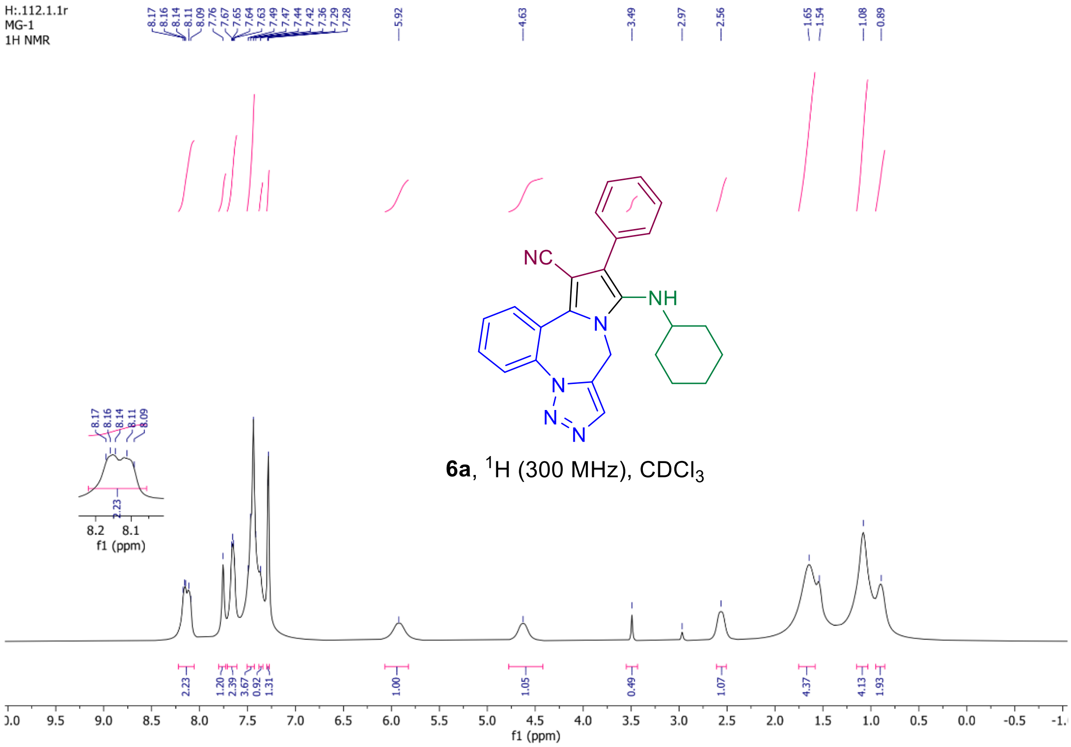

6a, ^{1}H (300 MHz), $CDCl_3$

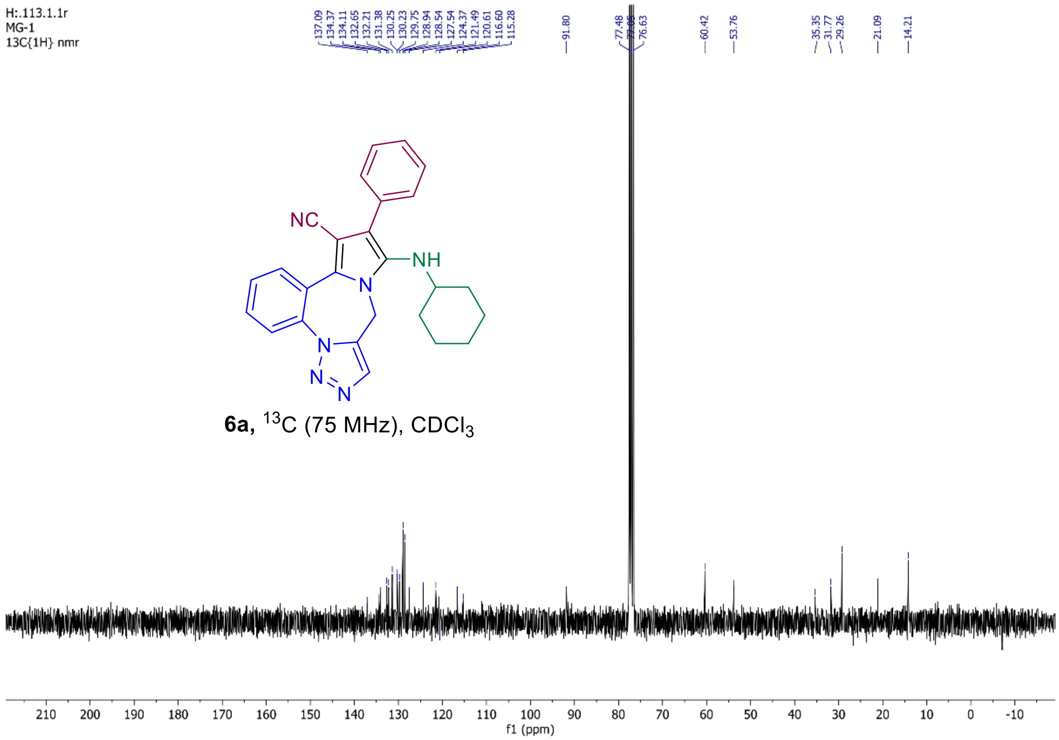

6a, ^{13}C (75 MHz), $CDCl_3$

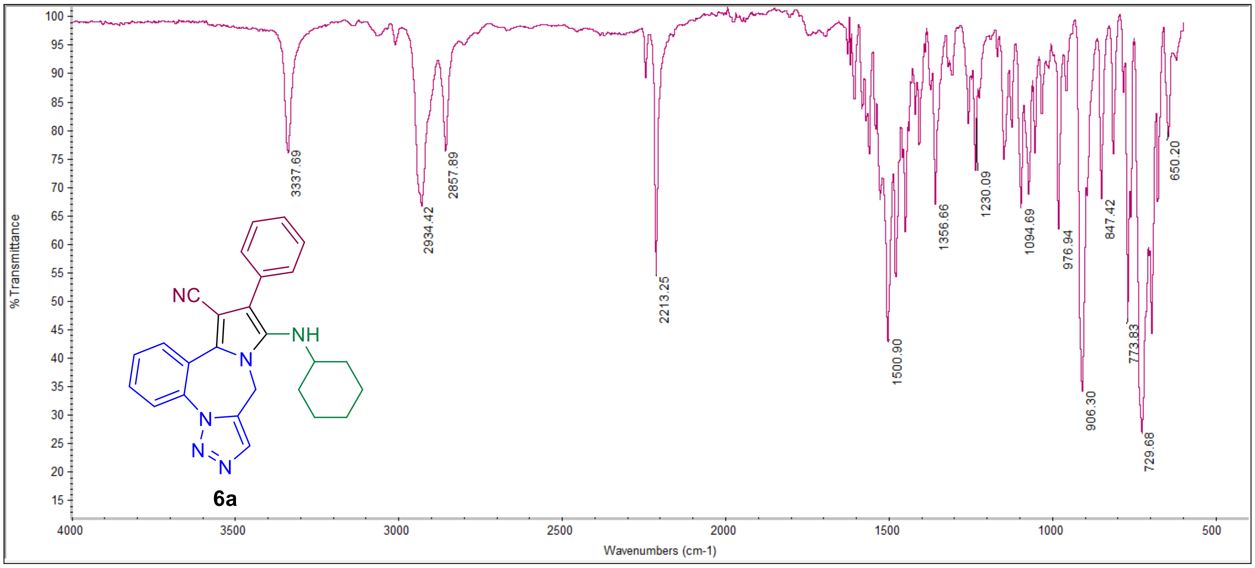
% Transmittance
Wavenumbers (cm-1)
3337.69
2934.42
2857.89
2213.25
1500.90
1356.66
1230.09
1094.69
976.94
906.30
847.42
773.83
729.68
650.20
NC
NH
N
N
N
N
6a

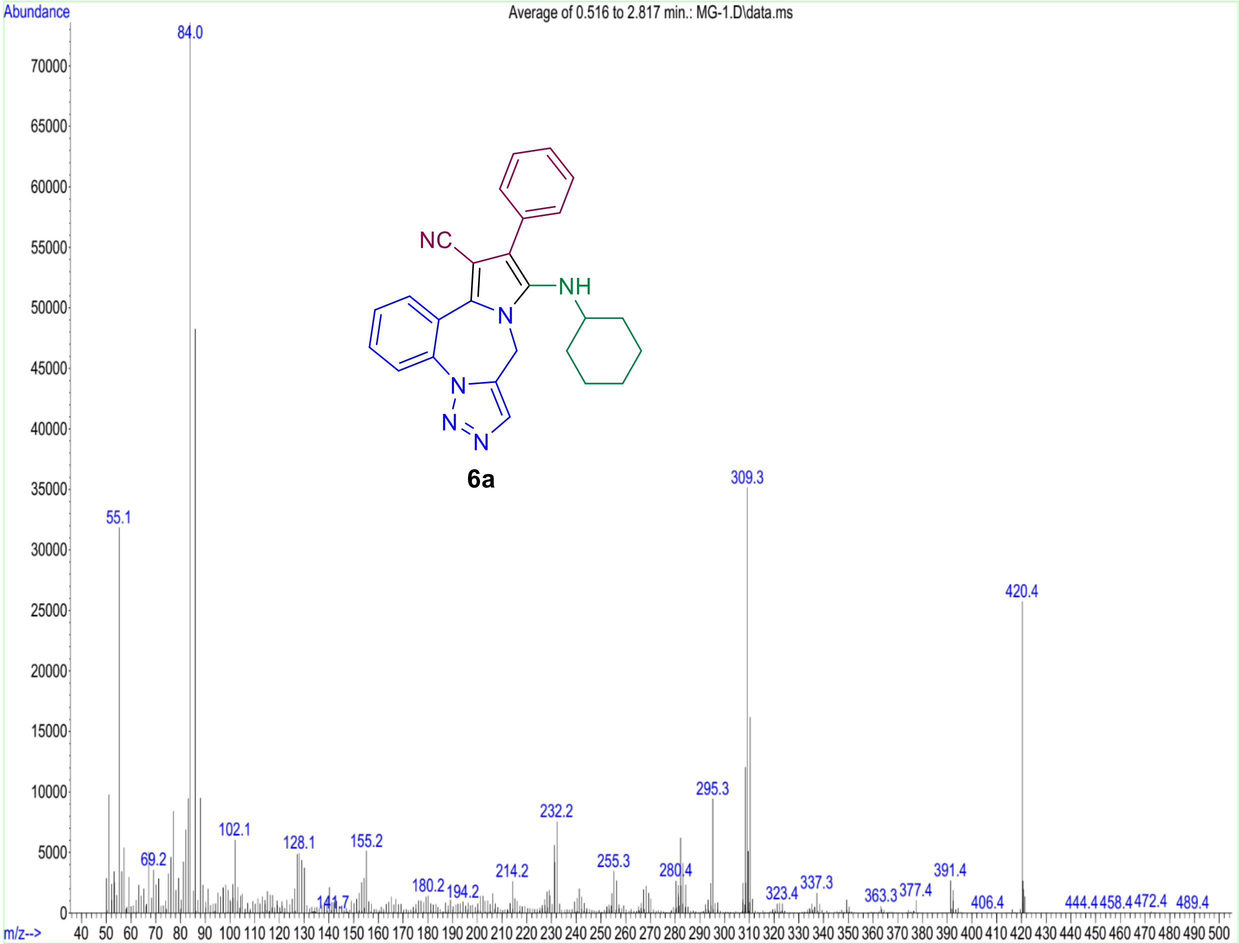
Abundance
Average of 0.516 to 2.817 min.: MG-1.D\data.ms
m/z-->
84.0
55.1
69.2
102.1
128.1
141.7
155.2
180.2
194.2
214.2
232.2
255.3
280.4
295.3
309.3
323.4
337.3
363.3
377.4
391.4
406.4
420.4
444.4
458.4
472.4
489.4
NC
NH
N
N
N
N
6a

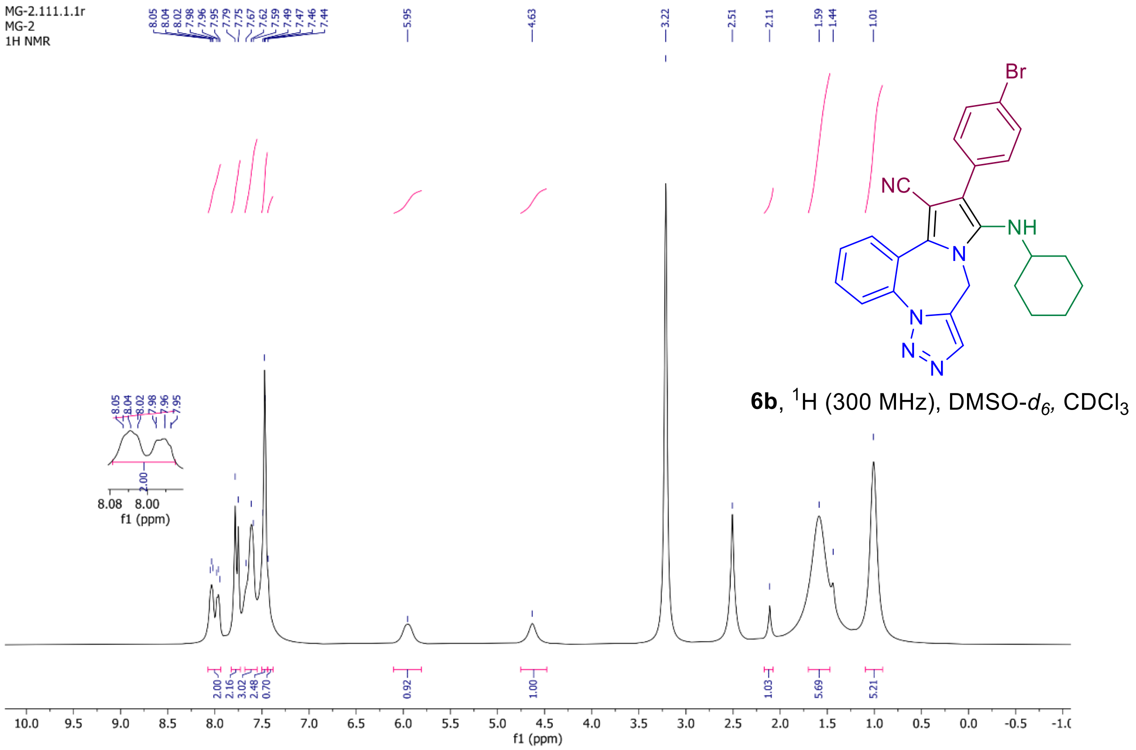
MG-2.111.1.1r
MG-2
1H NMR
8.05 8.04 8.02 7.98 7.96 7.95 7.79 7.75 7.67 7.62 7.59 7.49 7.47 7.46 7.44
5.95
4.63
3.22
2.51
2.11
1.59
1.44
1.01
6b, ¹H (300 MHz), DMSO-d6, CDCl3
f1 (ppm)

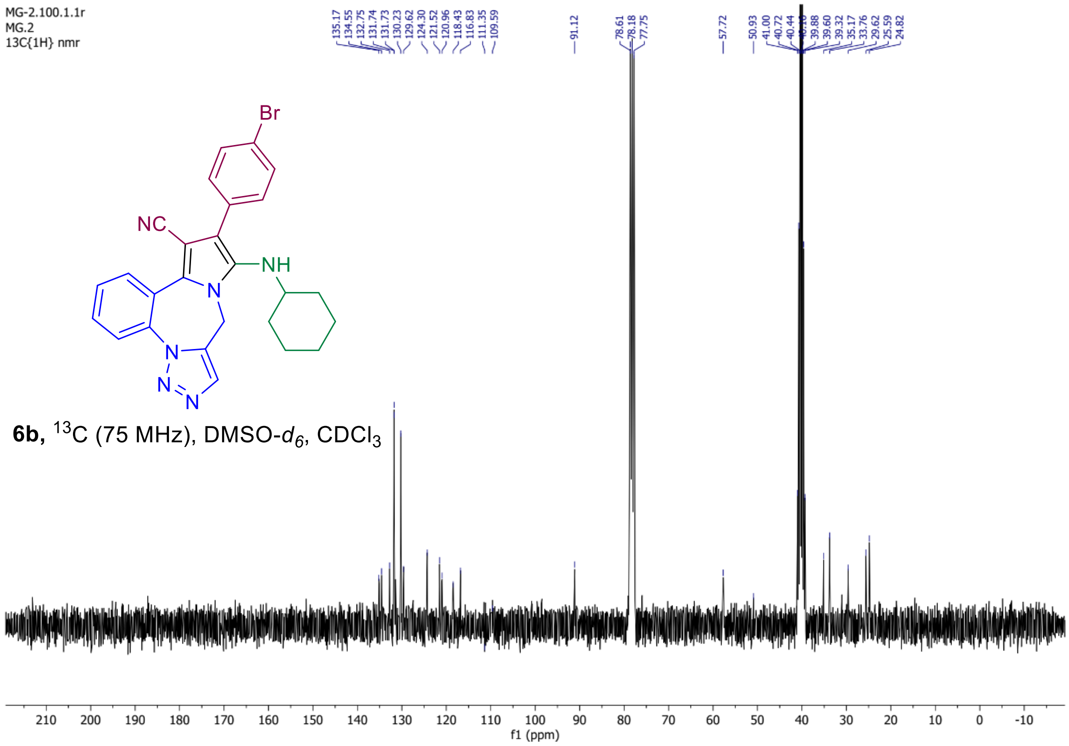
MG-2.100.1.1r
MG.2
13C{1H} nmr
135.17 134.55 132.75 131.74 131.73 130.23 129.62 124.30 121.52 120.96 118.43 116.83 111.35 109.59
91.12
78.61 78.18 77.75
57.72
50.93 41.00 40.72 40.44
39.88 39.60 39.32 35.17 33.76 29.62 25.59 24.82
6b, ¹³C (75 MHz), DMSO-d6, CDCl3
f1 (ppm)

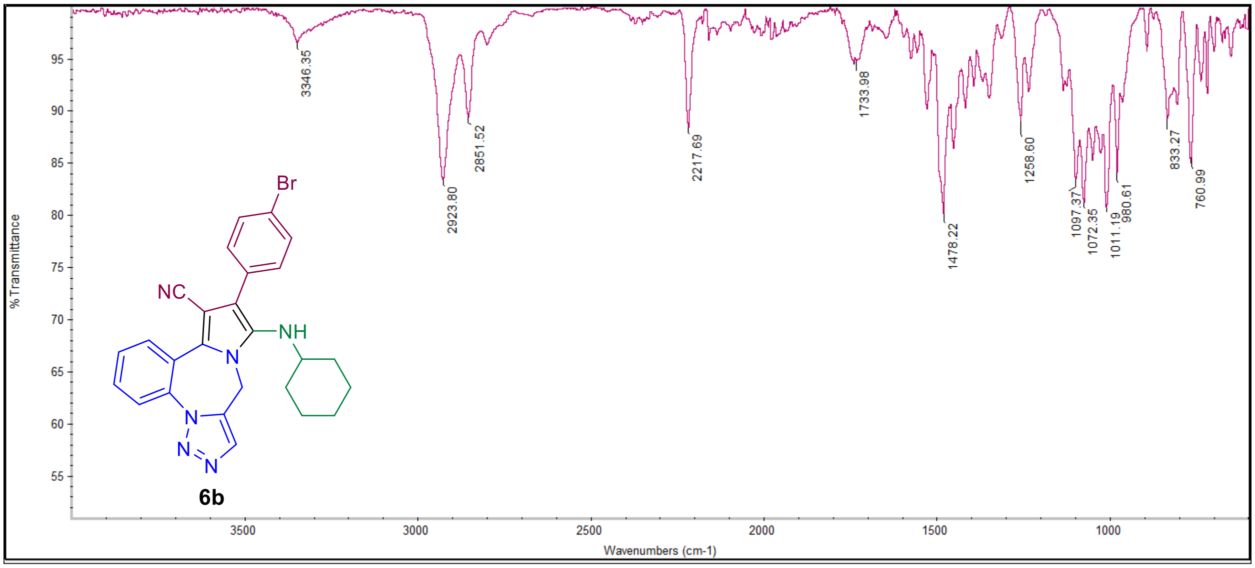

% Transmittance
Wavenumbers (cm-1)
3346.35
2923.80
2851.52
2217.69
1733.98
1478.22
1258.60
1097.37
1072.35
1011.19
980.61
833.27
760.99
Br
NC
NH
N
N
N
N
6b

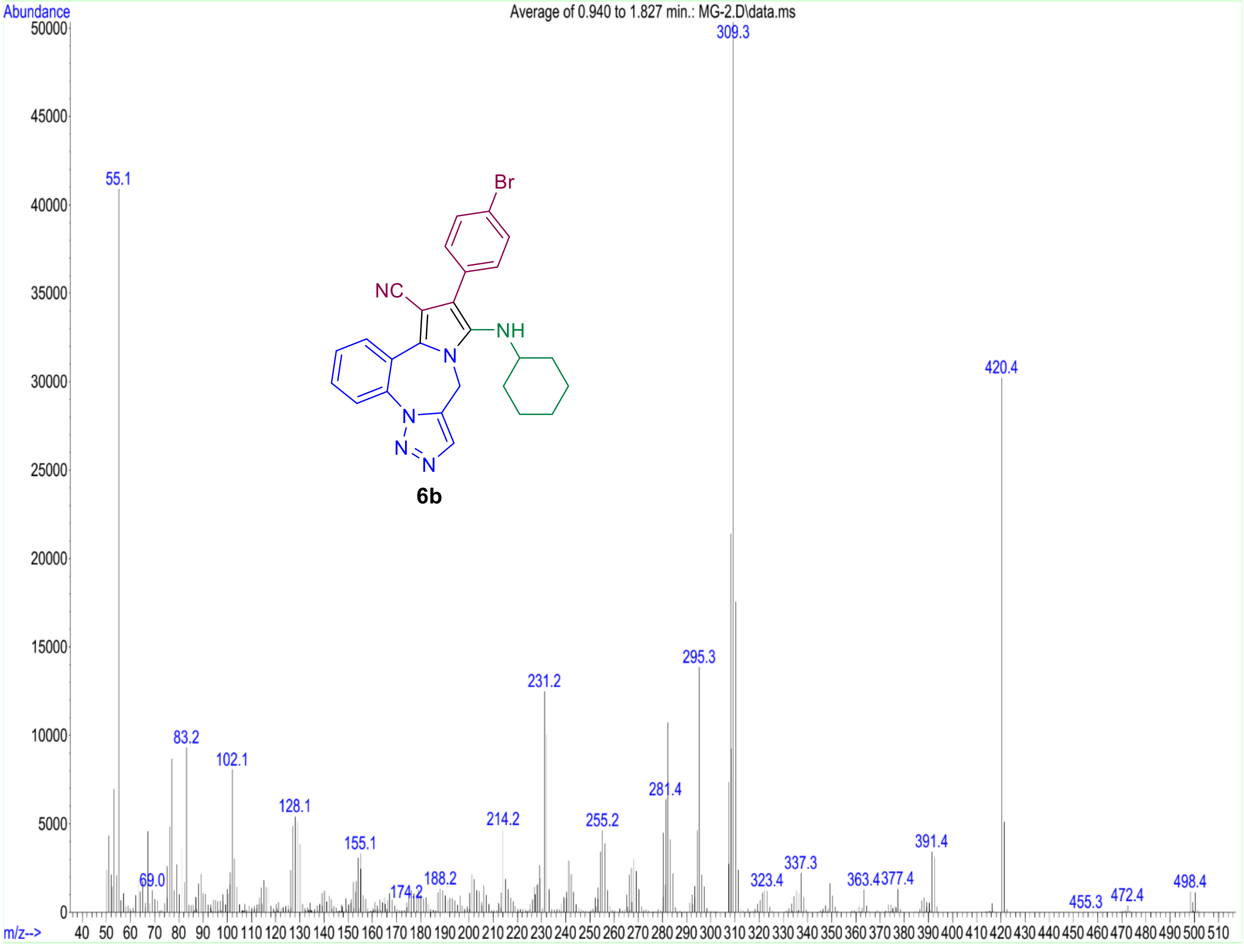

Abundance
Average of 0.940 to 1.827 min.: MG-2.D\data.ms
m/z-->
309.3
55.1
420.4
295.3
231.2
83.2
102.1
128.1
281.4
214.2
255.2
155.1
69.0
174.2
188.2
323.4
337.3
363.4
377.4
391.4
455.3
472.4
498.4
Br
NC
NH
N
N
N
N
6b

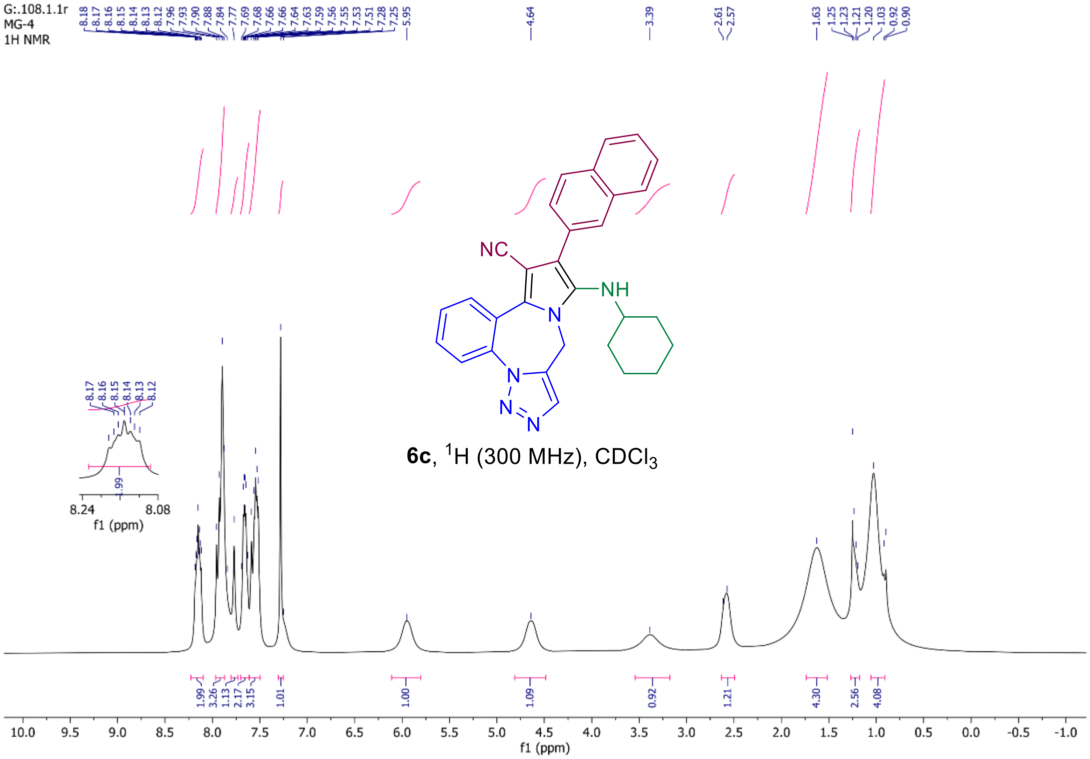
G:.108.1.1r
MG-4
1H NMR
NC
NH
N
N
N
N
6c, ¹H (300 MHz), CDCl₃
f1 (ppm)

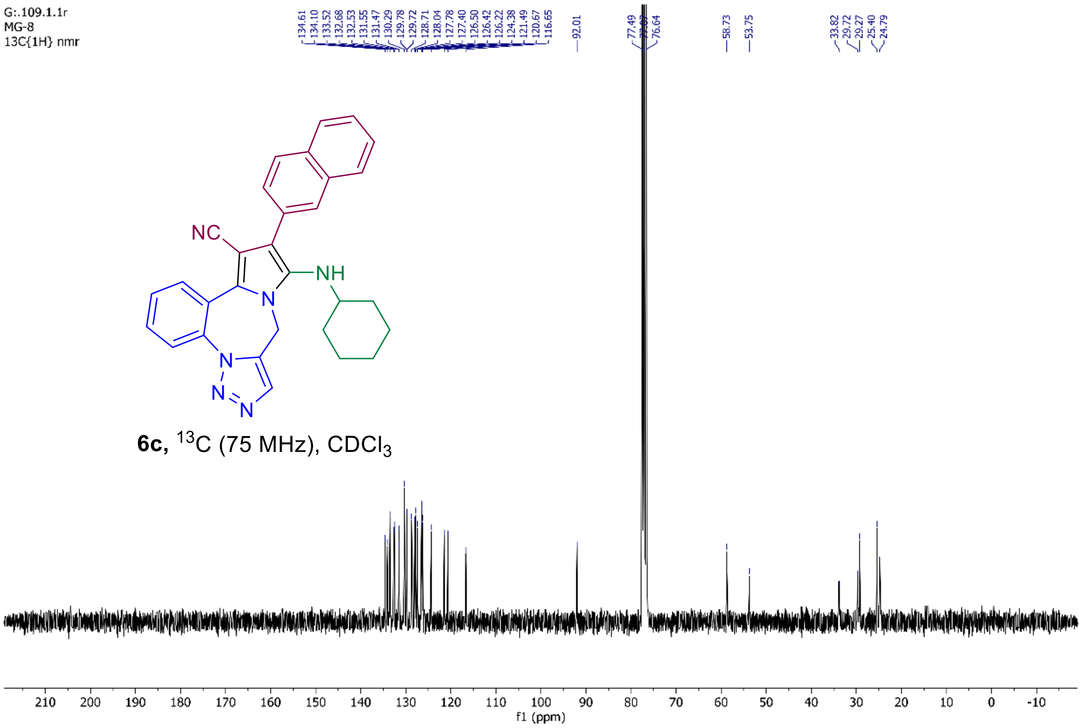
G:.109.1.1r
MG-8
13C{1H} nmr
NC
NH
N
N
N
N
6c, ¹³C (75 MHz), CDCl₃
f1 (ppm)

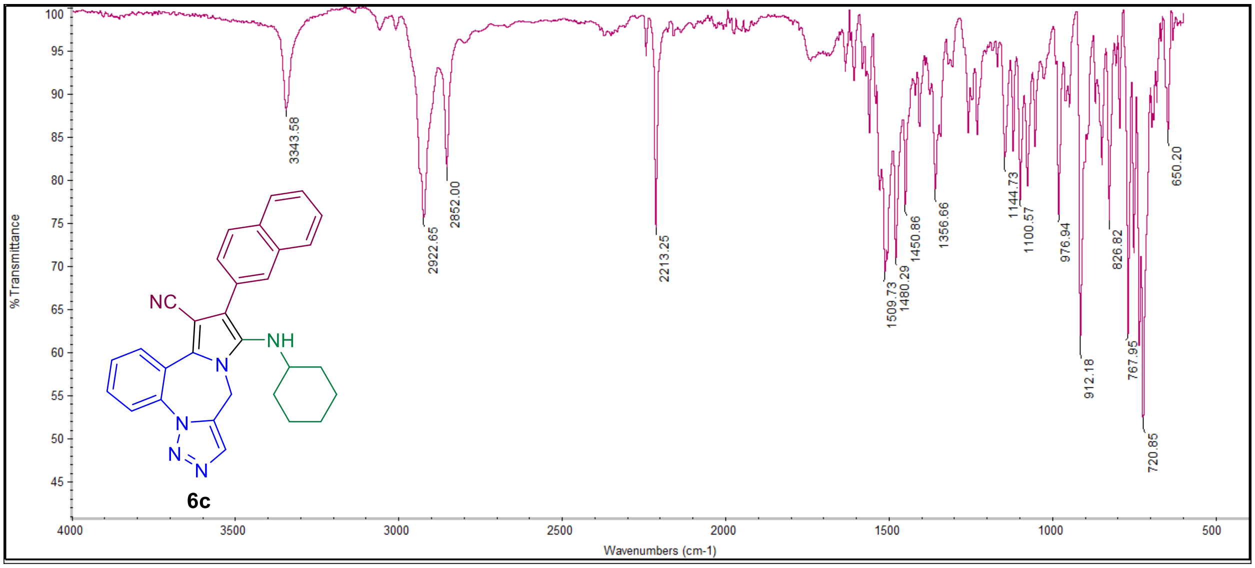
% Transmittance
Wavenumbers (cm-1)
3343.58
2922.65
2852.00
2213.25
1509.73
1480.29
1450.86
1356.66
1144.73
1100.57
976.94
912.18
826.82
767.95
720.85
650.20
NC
NH
N
N
N
N
6c

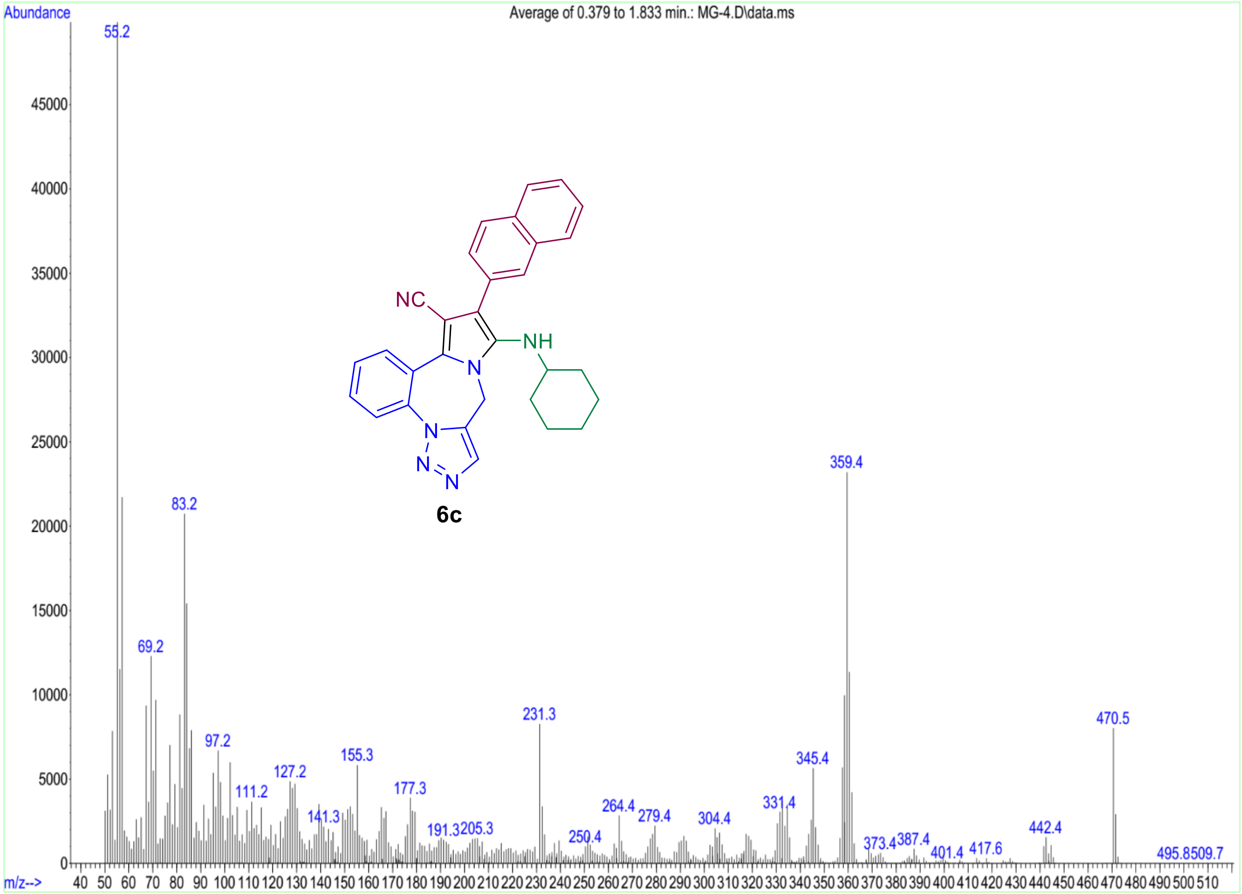
Abundance
Average of 0.379 to 1.833 min.: MG-4.D\data.ms
m/z-->
55.2
83.2
69.2
97.2
111.2
127.2
141.3
155.3
177.3
191.3
205.3
231.3
250.4
264.4
279.4
304.4
331.4
345.4
359.4
373.4
387.4
401.4
417.6
442.4
470.5
495.8
509.7
NC
NH
N
N
N
N
6c

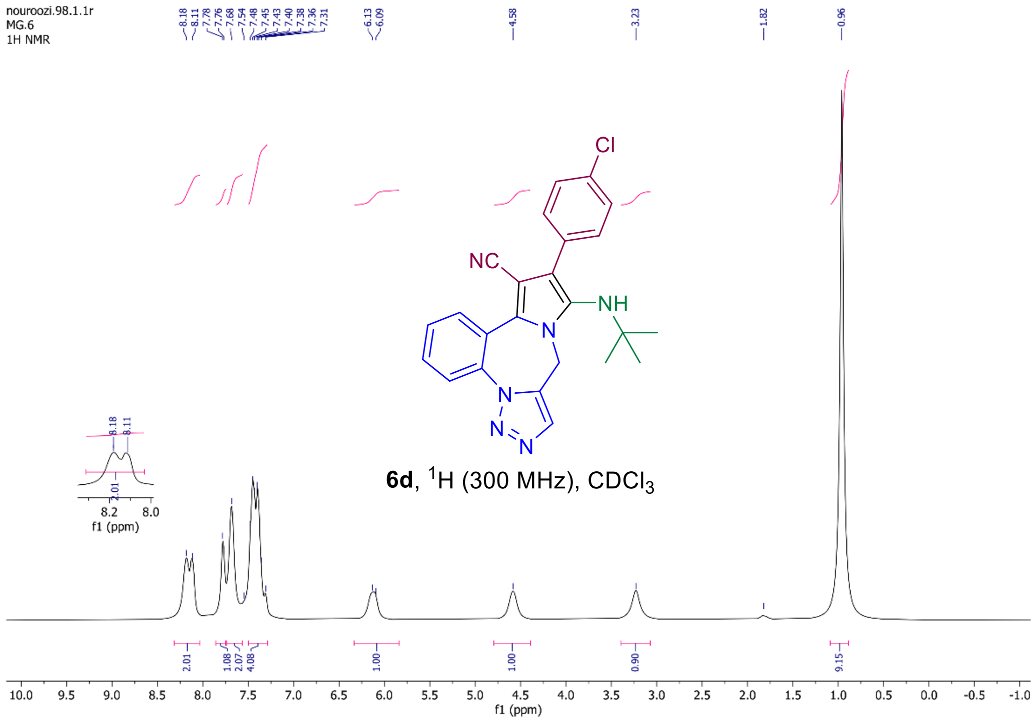
nouroozi.98.1.1r
MG.6
1H NMR
8.18
8.11
7.78
7.76
7.68
7.54
7.48
7.45
7.43
7.40
7.38
7.36
7.31
6.13
6.09
4.58
3.23
1.82
0.96
Cl
NC
NH
N
N
N
N
6d, ^{1}H (300 MHz), $CDCl_3$
2.01
1.08
2.07
4.08
1.00
1.00
0.90
9.15
f1 (ppm)

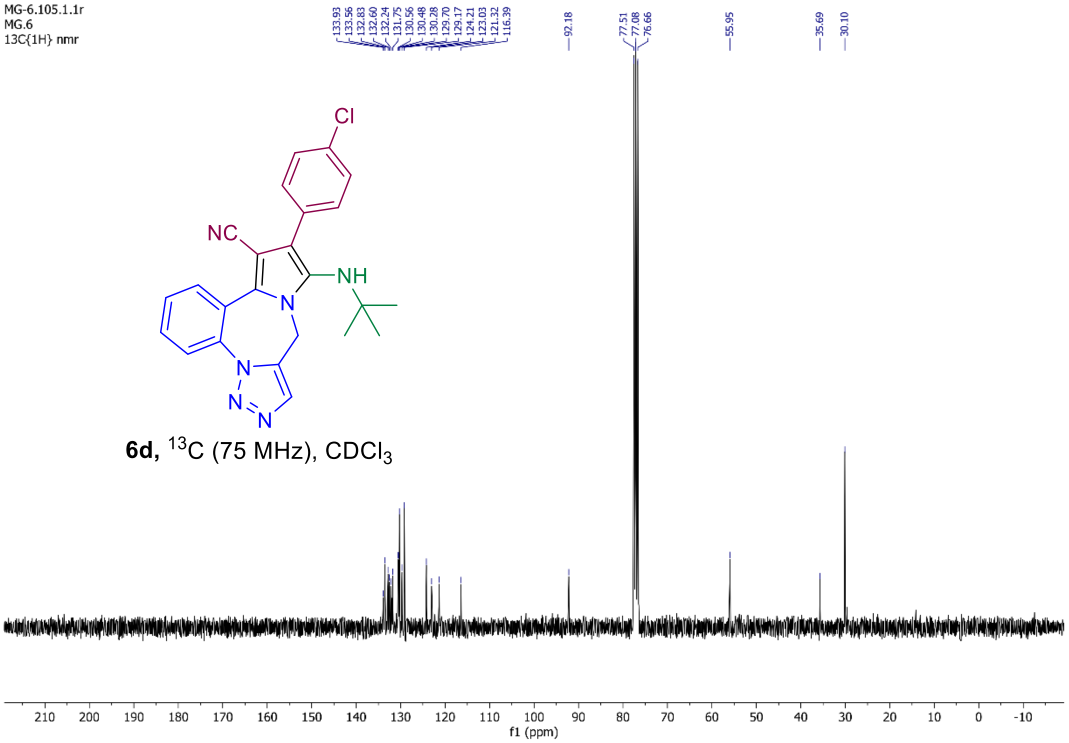
MG-6.105.1.1r
MG.6
13C{1H} nmr
133.93
133.56
132.83
132.60
132.24
131.75
130.56
130.48
130.28
129.70
129.17
124.21
123.03
121.32
116.39
92.18
77.51
77.08
76.66
55.95
35.69
30.10
Cl
NC
NH
N
N
N
N
6d, ^{13}C (75 MHz), $CDCl_3$
f1 (ppm)

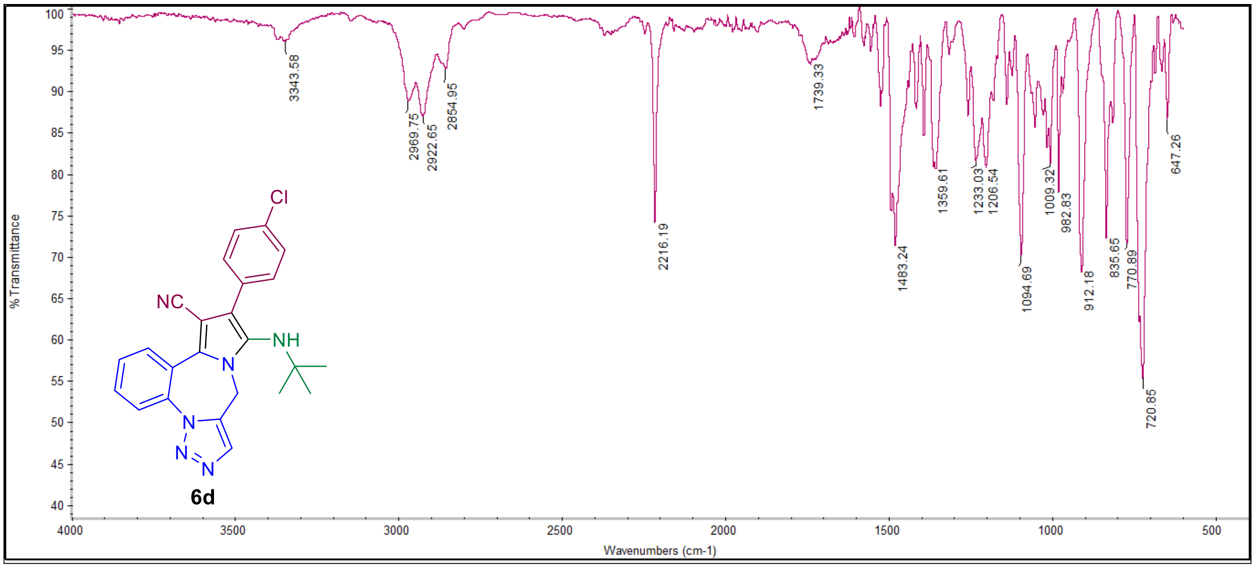
% Transmittance
Wavenumbers (cm-1)
3343.58
2969.75
2922.65
2854.95
2216.19
1739.33
1483.24
1359.61
1233.03
1206.54
1094.69
1009.32
982.83
912.18
835.65
770.89
720.85
647.26
Cl
NC
NH
N
N
N
N
6d

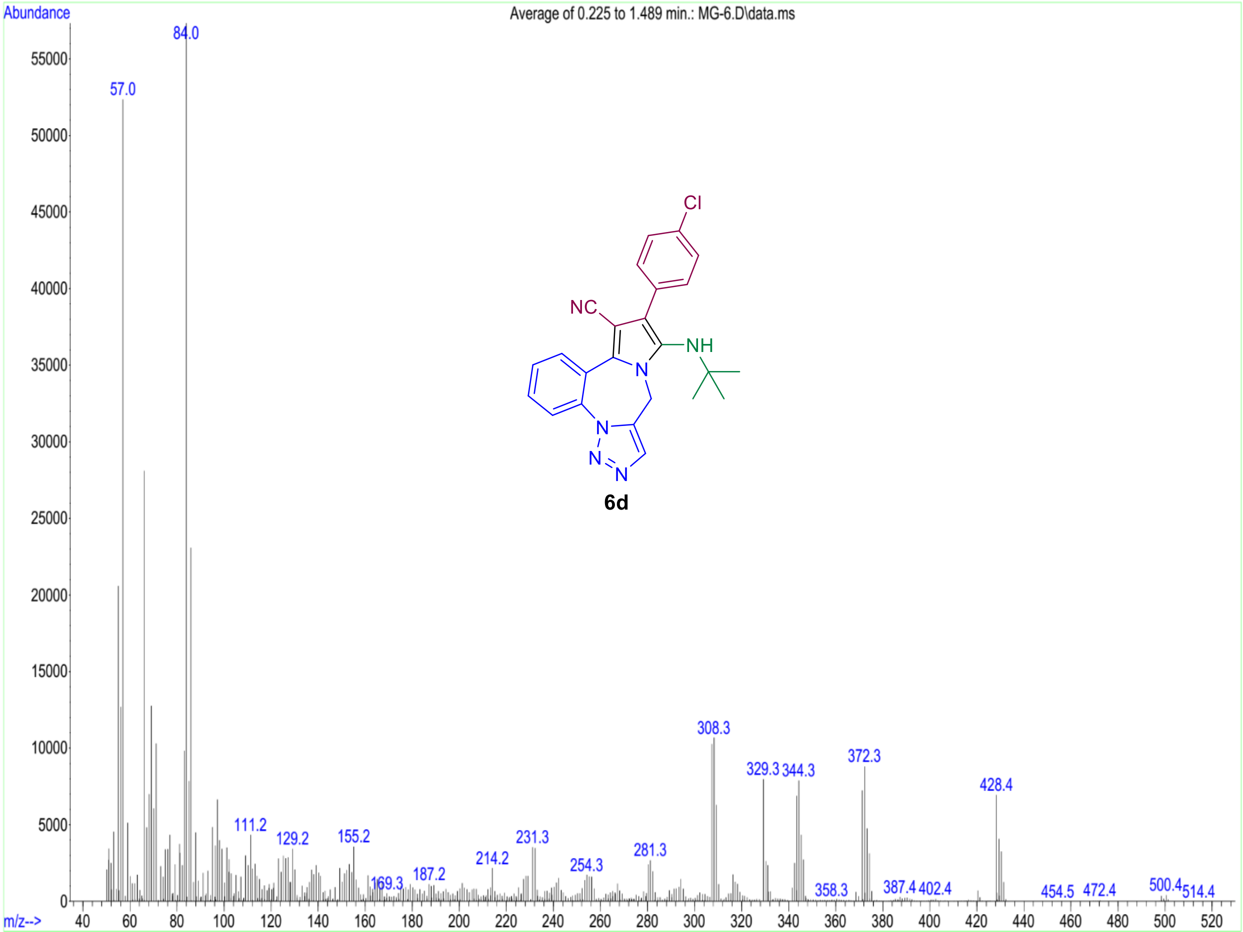
Abundance
Average of 0.225 to 1.489 min.: MG-6.D\data.ms
m/z-->
84.0
57.0
111.2
129.2
155.2
169.3
187.2
214.2
231.3
254.3
281.3
308.3
329.3
344.3
358.3
372.3
387.4
402.4
428.4
454.5
472.4
500.4
514.4
Cl
NC
NH
N
N
N
N
6d

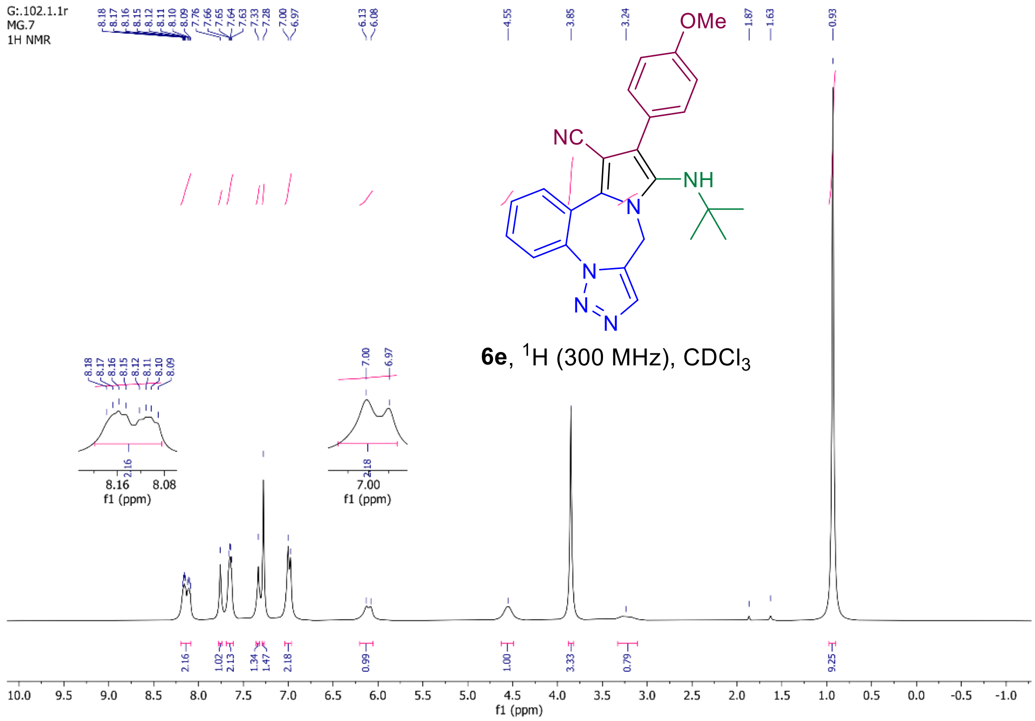

6e, ^{1}H (300 MHz), $CDCl_3$

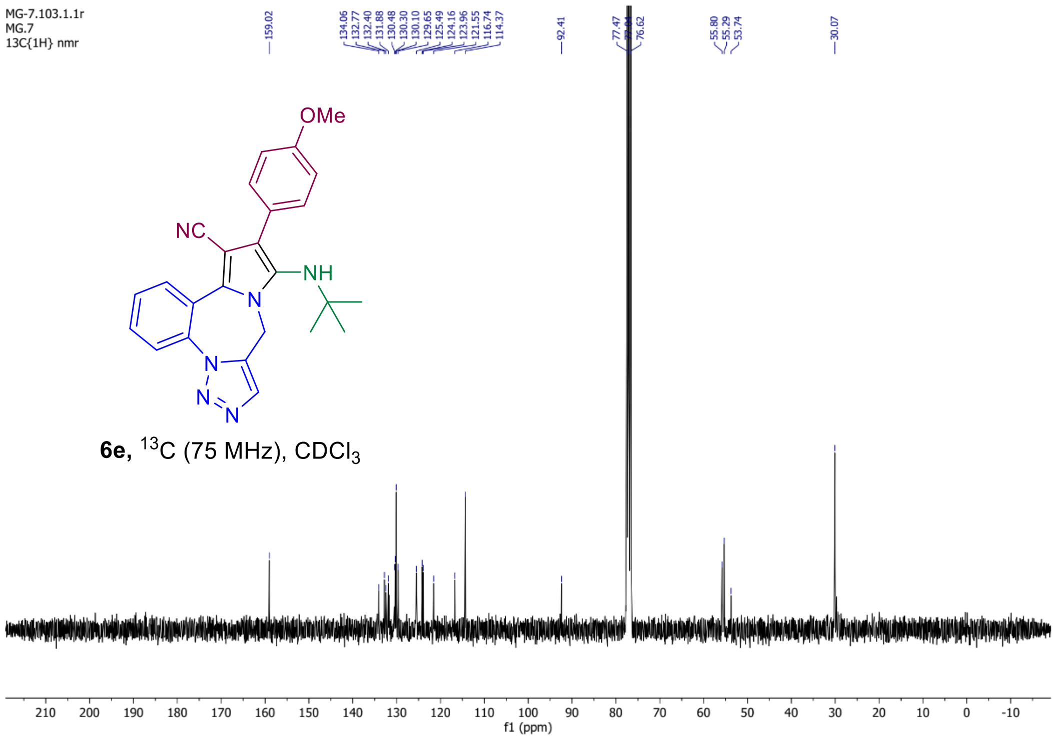

6e, ^{13}C (75 MHz), $CDCl_3$

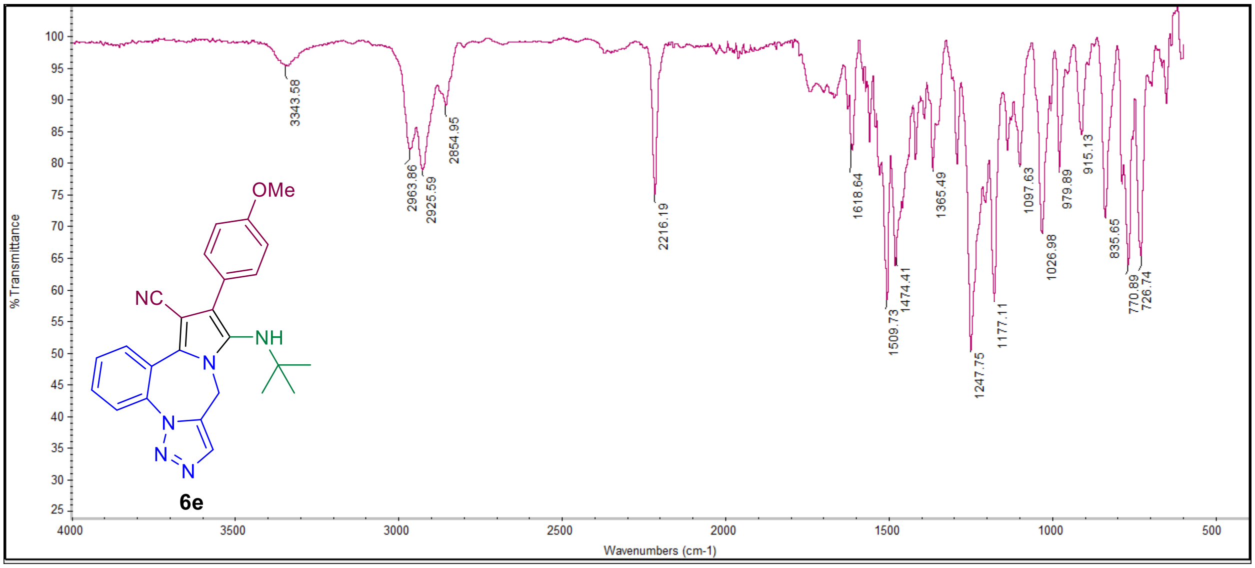
% Transmittance
Wavenumbers (cm-1)
3343.58
2963.86
2925.59
2854.95
2216.19
1618.64
1509.73
1474.41
1365.49
1247.75
1177.11
1097.63
1026.98
979.89
915.13
835.65
770.89
726.74
OMe
NC
NH
N
N
N
N
6e

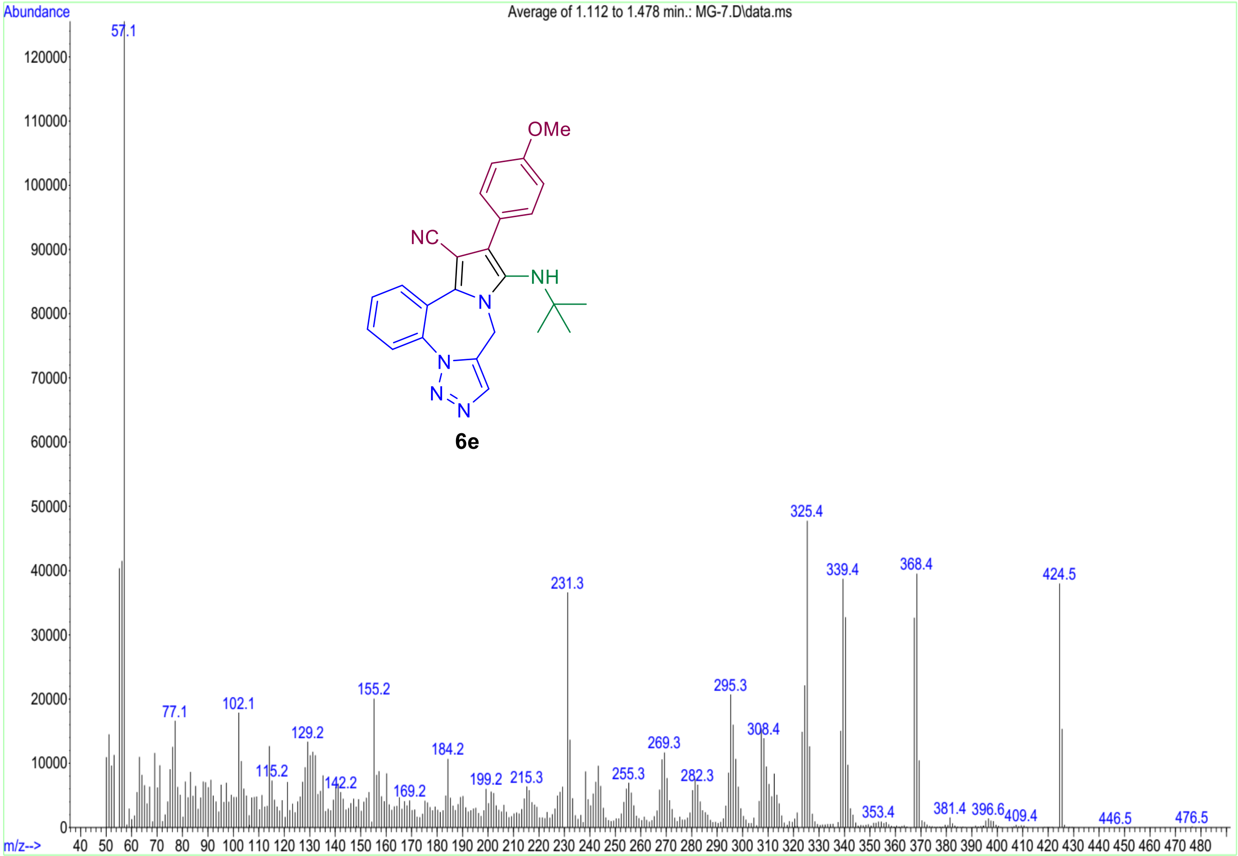
Abundance
Average of 1.112 to 1.478 min.: MG-7.D\data.ms
m/z-->
57.1
77.1
102.1
115.2
129.2
142.2
155.2
169.2
184.2
199.2
215.3
231.3
255.3
269.3
282.3
295.3
308.4
325.4
339.4
353.4
368.4
381.4
396.6
409.4
424.5
446.5
476.5
OMe
NC
NH
N
N
N
N
6e

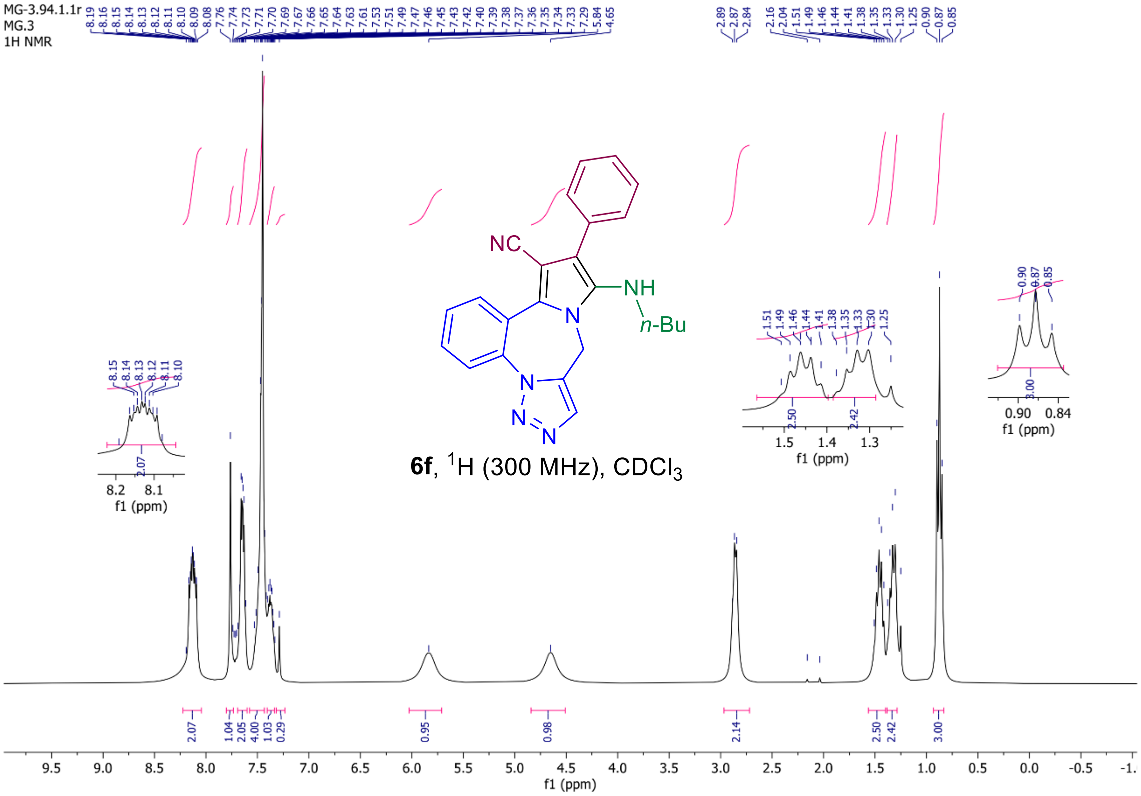

6f, ^{1}H (300 MHz), $CDCl_3$

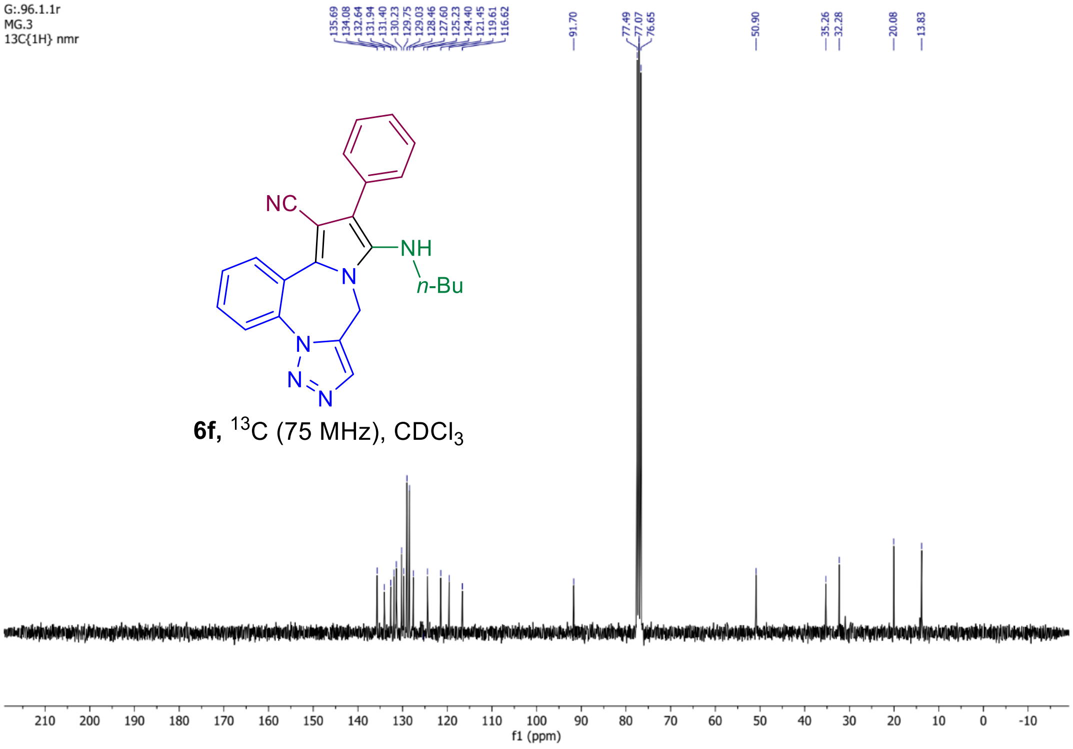

6f, ^{13}C (75 MHz), $CDCl_3$

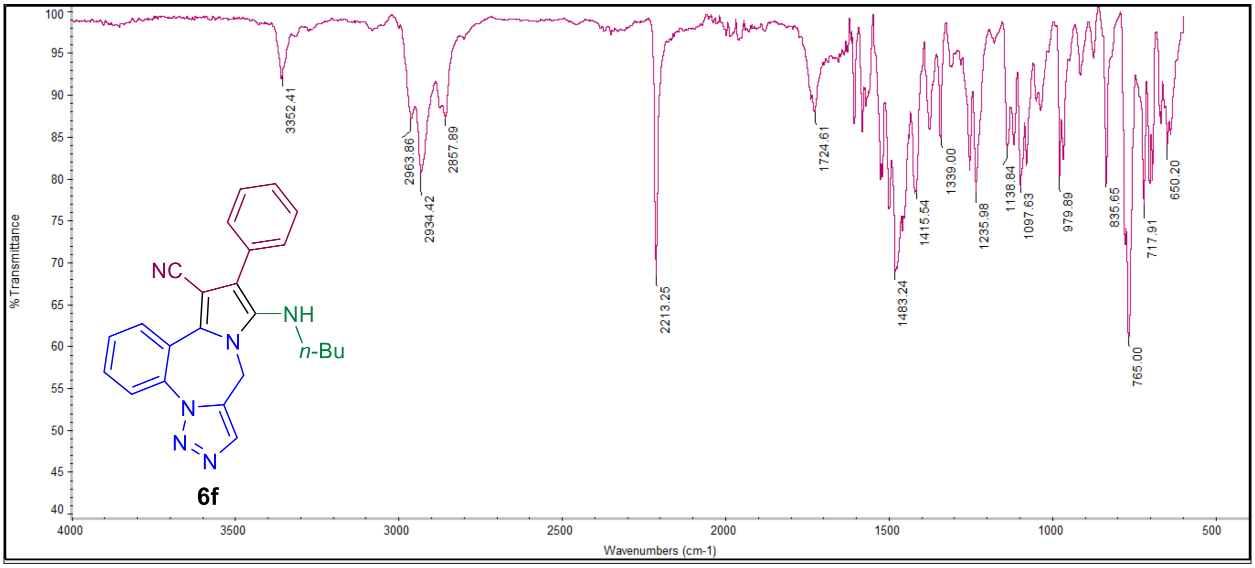

% Transmittance
Wavenumbers (cm-1)
3352.41
2963.86
2934.42
2857.89
2213.25
1724.61
1483.24
1415.54
1339.00
1235.98
1138.84
1097.63
979.89
835.65
765.00
717.91
650.20
NC
NH
n-Bu
N
6f

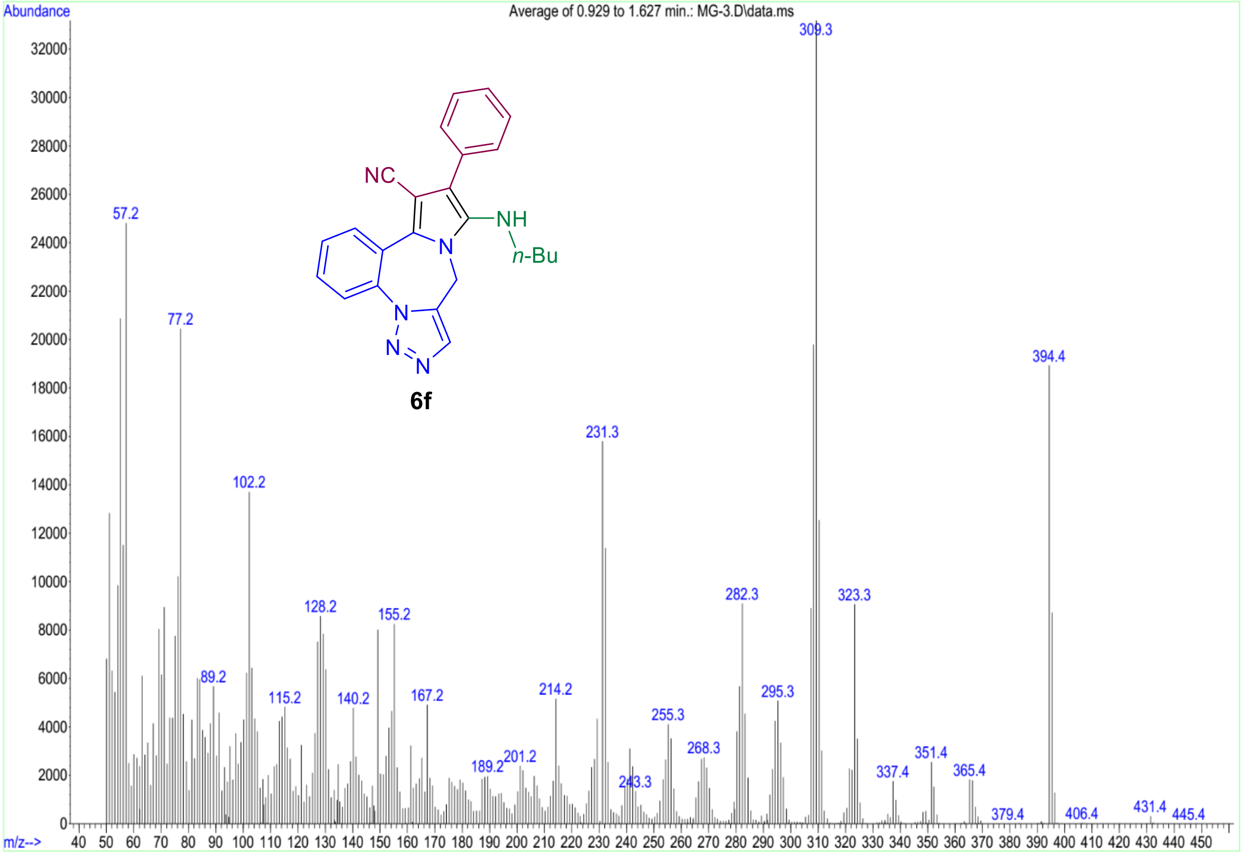

Abundance
Average of 0.929 to 1.627 min.: MG-3.D\data.ms
m/z-->
309.3
57.2
77.2
394.4
231.3
102.2
128.2
155.2
282.3
323.3
89.2
115.2
140.2
167.2
214.2
255.3
295.3
189.2
201.2
243.3
268.3
337.4
351.4
365.4
379.4
406.4
431.4
445.4
NC
NH
n-Bu
6f

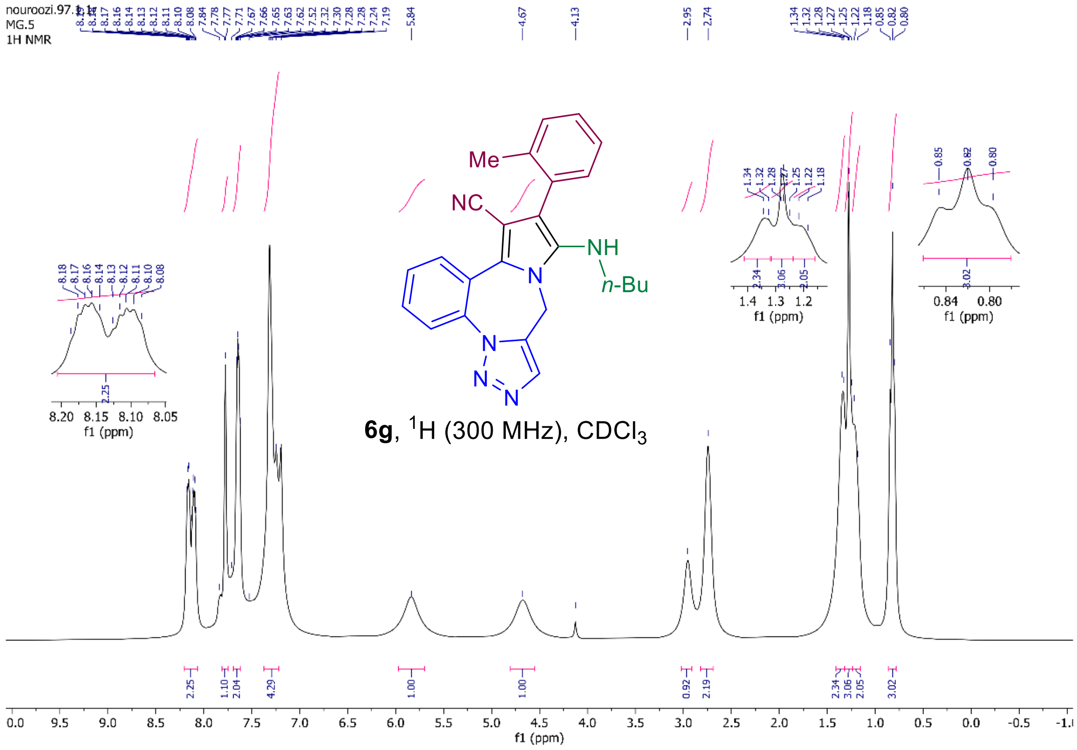

6g, ^{1}H (300 MHz), $CDCl_3$

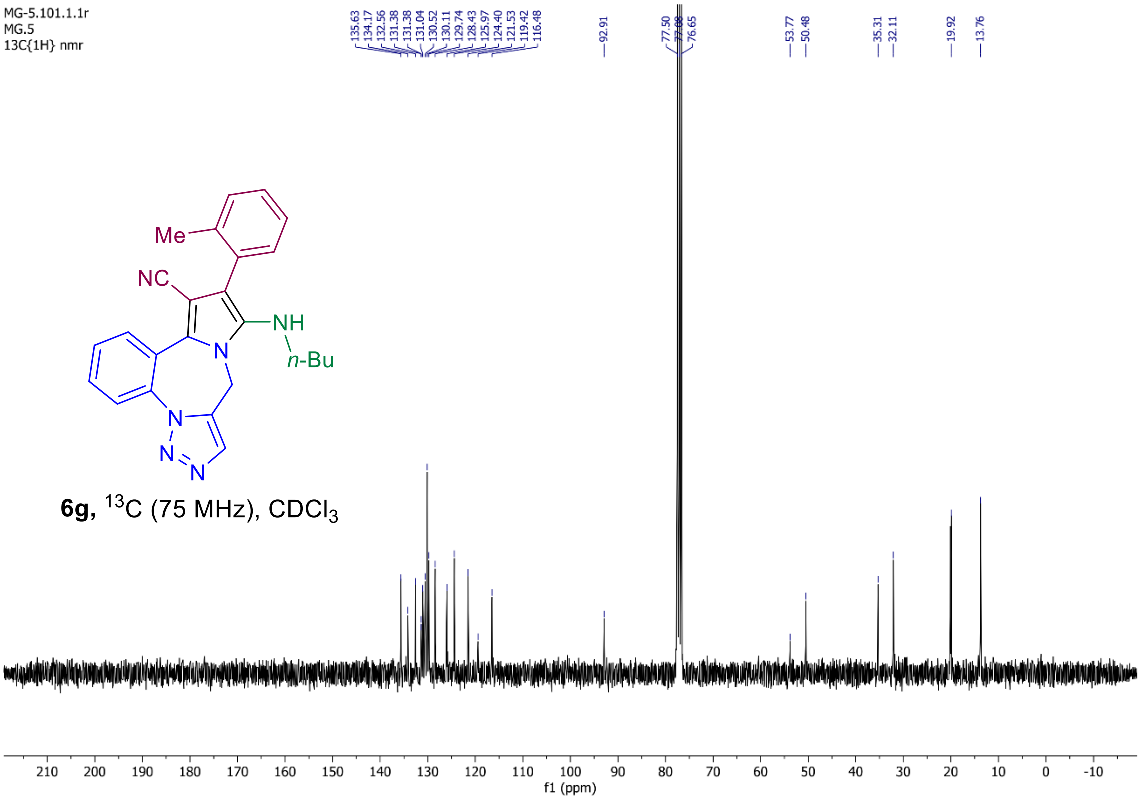

6g, ^{13}C (75 MHz), $CDCl_3$

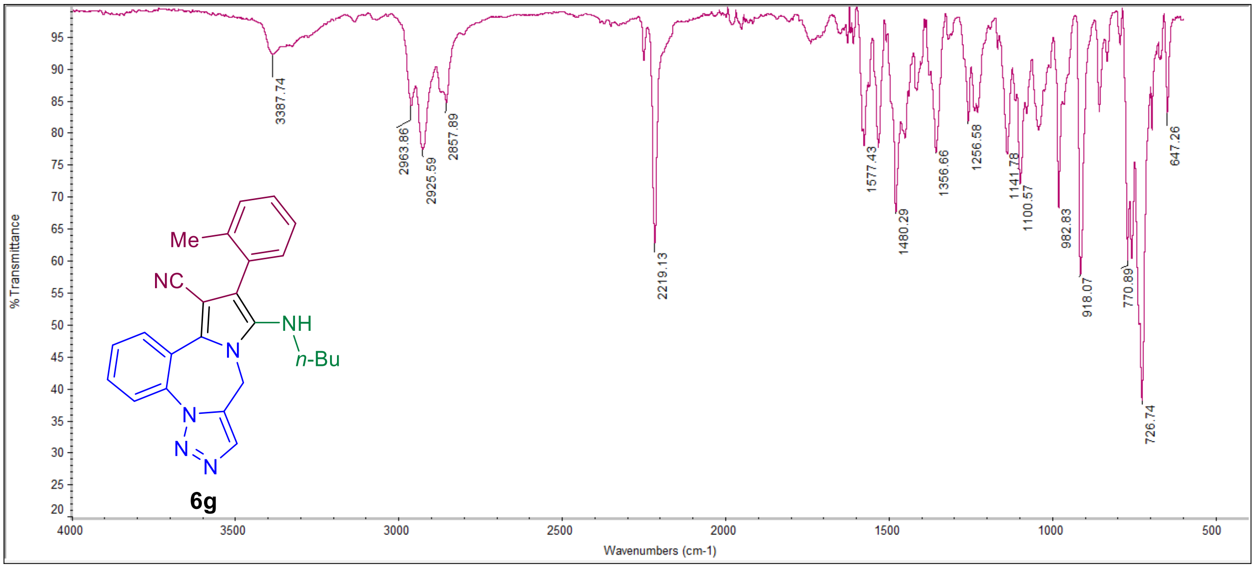
% Transmittance
Wavenumbers (cm-1)
3387.74
2963.86
2925.59
2857.89
2219.13
1577.43
1480.29
1356.66
1256.58
1141.78
1100.57
982.83
918.07
770.89
726.74
647.26
Me
NC
NH
n-Bu
N
N
N
N
6g

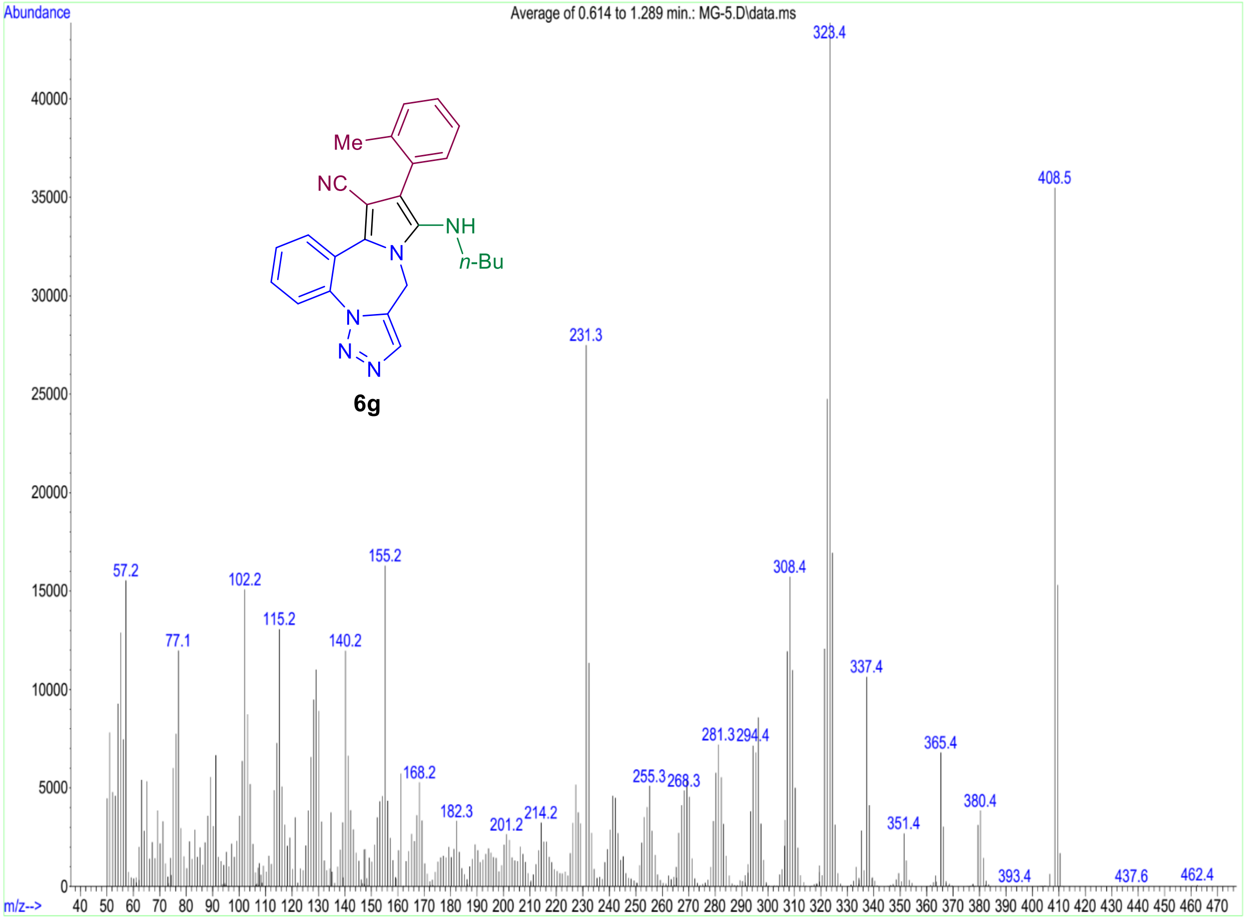
Abundance
Average of 0.614 to 1.289 min.: MG-5.D\data.ms
m/z-->
323.4
408.5
231.3
57.2
77.1
102.2
115.2
140.2
155.2
168.2
182.3
201.2
214.2
255.3
268.3
281.3
294.4
308.4
337.4
351.4
365.4
380.4
393.4
437.6
462.4
Me
NC
NH
n-Bu
6g

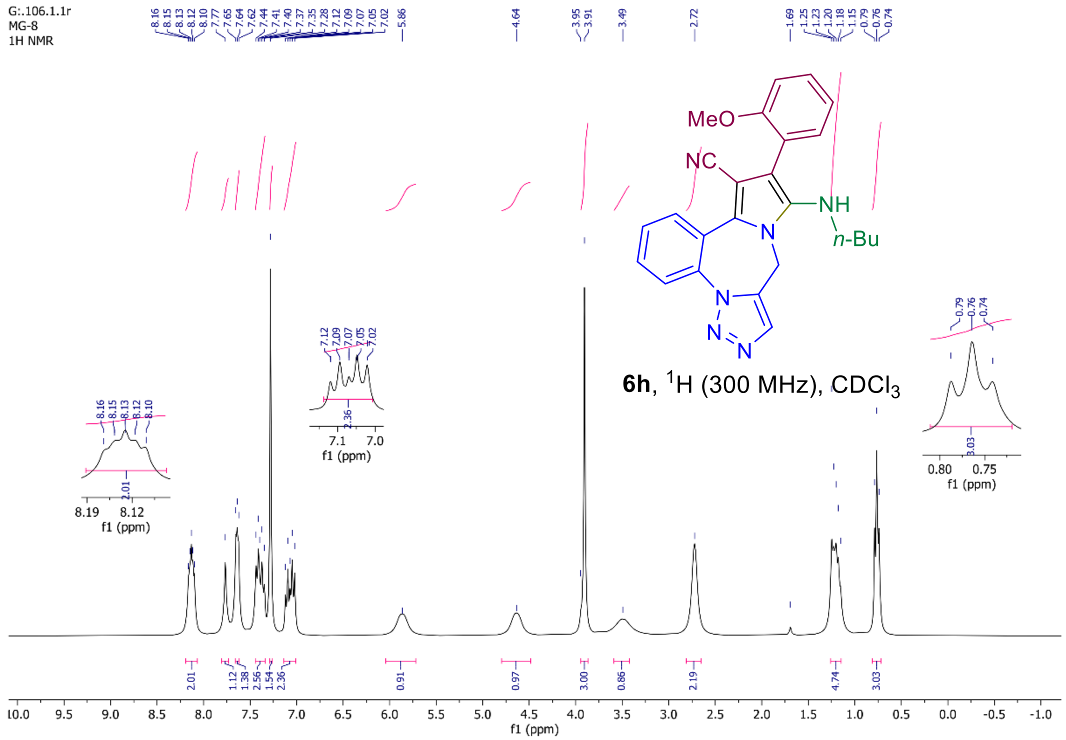

6h, ^{1}H (300 MHz), $CDCl_3$

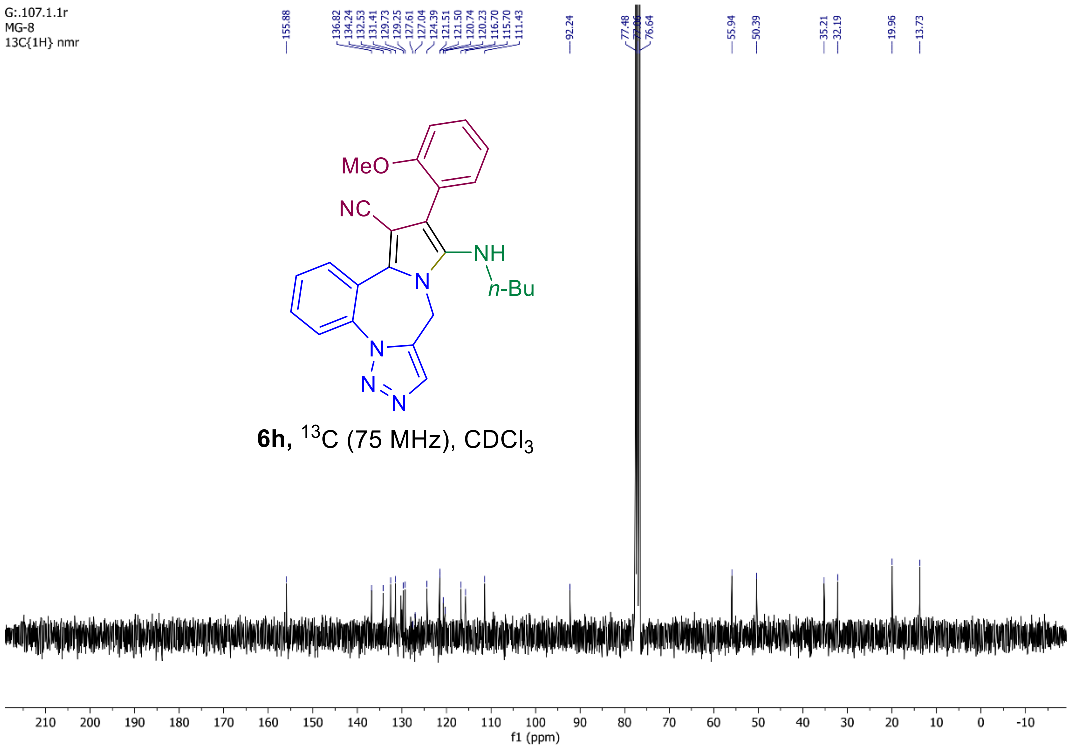

6h, ^{13}C (75 MHz), $CDCl_3$

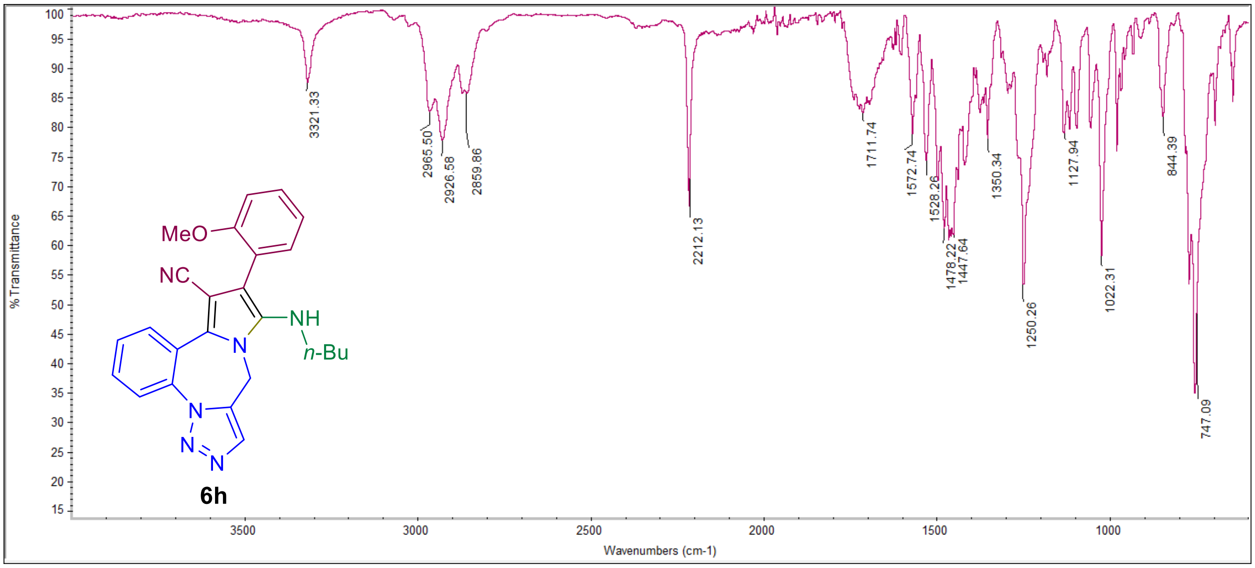
% Transmittance
Wavenumbers (cm-1)
3321.33
2965.50
2926.58
2859.86
2212.13
1711.74
1572.74
1528.26
1478.22
1447.64
1350.34
1250.26
1127.94
1022.31
844.39
747.09
MeO
NC
NH
n-Bu
N
N
N
N
6h

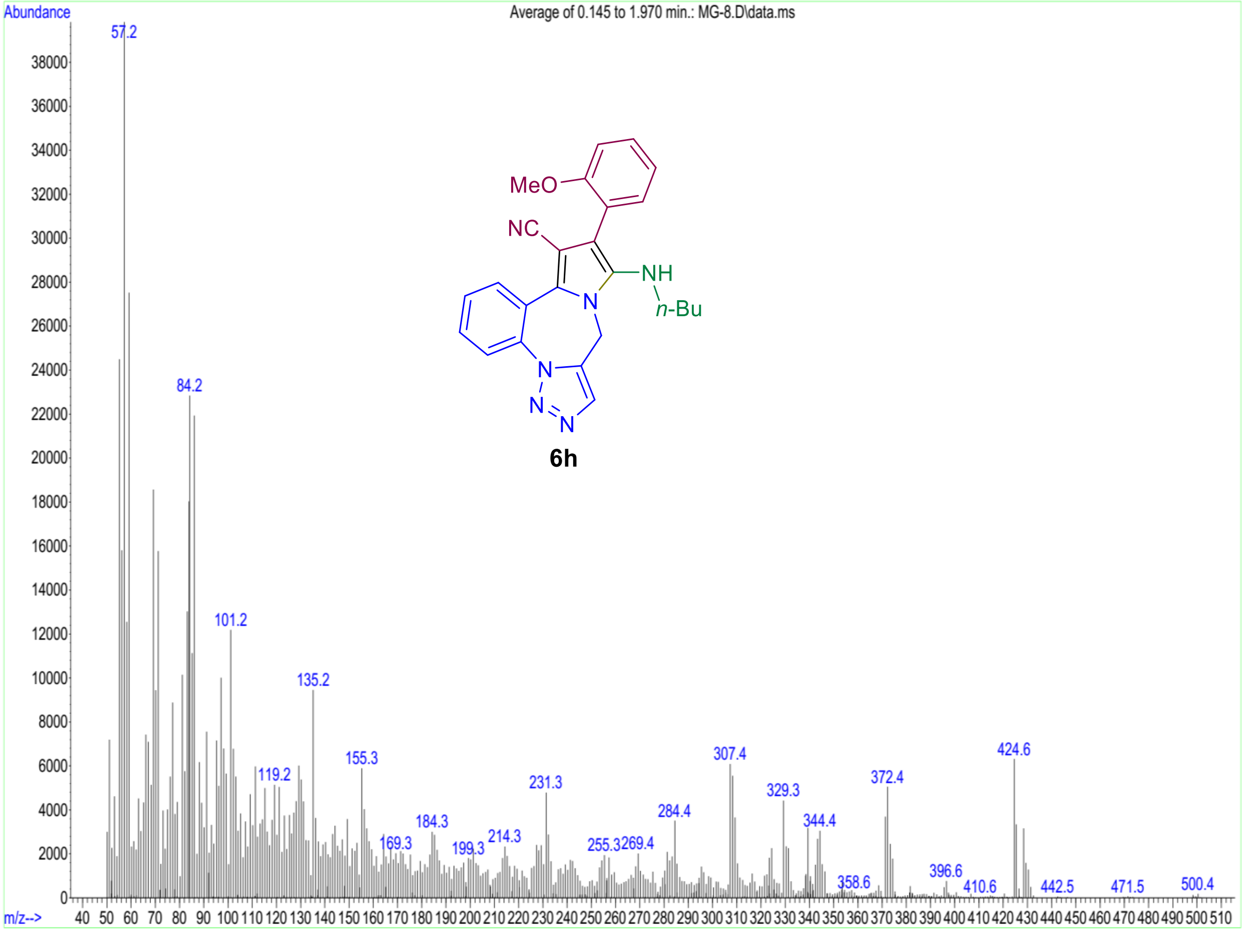
Abundance
Average of 0.145 to 1.970 min.: MG-8.D\data.ms
m/z-->
57.2
84.2
101.2
119.2
135.2
155.3
169.3
184.3
199.3
214.3
231.3
255.3
269.4
284.4
307.4
329.3
344.4
358.6
372.4
396.6
410.6
424.6
442.5
471.5
500.4
MeO
NC
NH
n-Bu
N
N
N
N
6h

Reference

1. S. Sreejith, K. P. Divya and A. Ajayaghosh, *Chem. Commun.*, 2008, 25, 2903-2905.
